# Monodisperse Molecular Models for the sp Carbon Allotrope Carbyne; Syntheses, Structures, and Properties of Diplatinum Polyynediyl Complexes with **PtC_20_Pt** to **PtC_52_Pt** Linkages

**DOI:** 10.1021/acscentsci.3c01090

**Published:** 2023-11-16

**Authors:** Aayushi Arora, Sourajit Dey Baksi, Nancy Weisbach, Hashem Amini, Nattamai Bhuvanesh, John A. Gladysz

**Affiliations:** Department of Chemistry, Texas A&M University, P.O. Box 30012, College Station, Texas 77842-3012, United States

## Abstract

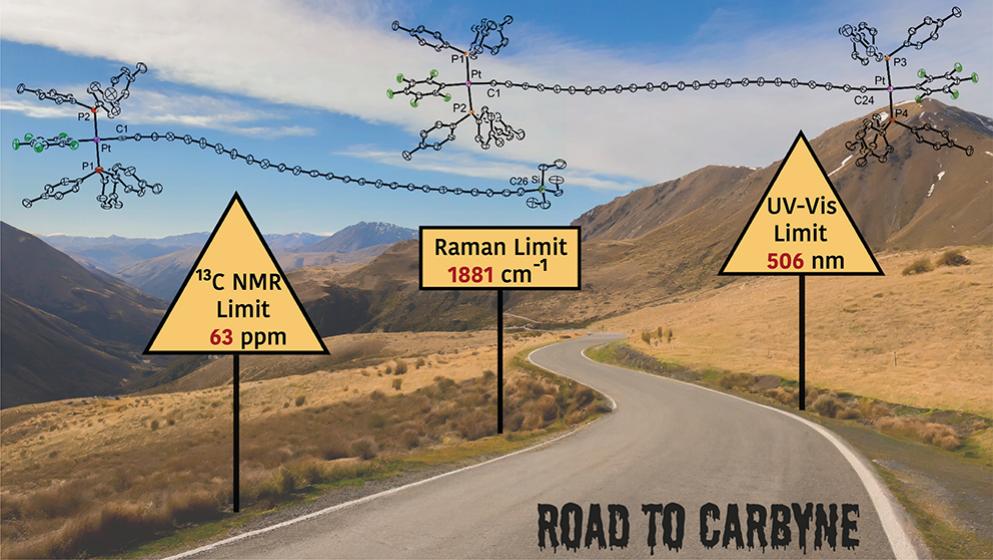

Extended conjugated polyynes provide models for the elusive sp carbon polymer carbyne, but progress has been hampered by numerous synthetic challenges. Stabilities appear to be enhanced by bulky, electropositive transition-metal endgroups. Reactions of *trans*-(C_6_F_5_)(*p*-tol_3_P)_2_Pt(C≡C)_*n*_SiEt_3_ (*n* = 4–6, **PtC**_***x***_**Si** (*x* = 2*n*)) with *n*-Bu_4_N^+^F^–^/Me_3_SiCl followed by excess tetrayne H(C≡C)_4_SiEt_3_ (**HC**_**8**_**Si**) and then CuCl/TMEDA and O_2_ give the heterocoupling products **PtC**_*x***+8**_**Si**, **PtC**_*x***+16**_**Si**, and sometimes higher homologues. The **PtC**_*x***+16**_**Si** species presumably arise via protodesilylation of **PtC**_*x***+8**_**Si** under the reaction conditions. Chromatography allows the separation of **PtC**_**16**_**Si**, **PtC**_**24**_**Si**, and **PtC**_**32**_**Si** (from *n* = 4), **PtC**_**18**_**Si** and **PtC**_**26**_**Si** (*n* = 5), or **PtC**_**20**_**Si** and **PtC**_**28**_**Si** (*n* = 6). These and previously reported species are applied in similar oxidative homocouplings, affording the family of diplatinum polyynediyl complexes **PtC**_***x***_**Pt** (*x* = 20, 24, 28, 32, 36, 40 in 96–34% yields and *x* = 44, 48, 52 in 22–7% yields). These are carefully characterized by ^13^C NMR, UV–visible, and Raman spectroscopy and other techniques, with particular attention to behavior as the C_*x*_ chain approaches the macromolecular limit and endgroup effects diminish. The crystal structures of solvates of **PtC**_**20**_**Pt**, **PtC**_**24**_**Pt**, and **PtC**_**26**_**Si**, which feature the longest sp chains structurally characterized to date, are analyzed in detail. All data support a polyyne electronic structure with a nonzero optical band gap and bond length alternation for carbyne.

## Abstract 2

It has been a long road to the sp carbon allotrope carbyne, littered with many explosions (literal) and car wrecks (figurative), but synthetic chemists are closing in on the end phase of this journey.

## Introduction

Carbon is the second most abundant element in the human body on a mass basis and holds fourth place in interstellar space.^1^ It exhibits extraordinarily versatile bonding properties, manifested in three limiting hybridization states (sp^3^, sp^2^, and sp). Although phenomena involving carbon and other elements are of immense fundamental importance, there remain major challenges in understanding many systems composed of carbon alone.^2^ Over the last three decades, the classical and extensively studied polymeric sp^3^ and sp^2^ allotropes, diamond^3^ and graphite,^4^ have been augmented by a plethora of new molecular and polymeric forms of carbon. These include, among many others,^2^ the fullerenes,^5^ nanotubes,^6^ and graphene.^7^

The work reported herein relates to the polymeric sp allotrope of carbon, commonly termed “carbyne” (**C**_**∞**_). There is extensive literature on this material,^8^ for which both polyyne (−(C≡C−)_∞_) and cumulated (=(C=C=)_∞_) electronic structures have received consideration. The former is distinguished by significant bond order or bond length alternation (BLA)^9^ and is generally thought to be more stable.^10^ However, to date there is no undisputed^11^ confirmation that carbyne can exist as a pure bulk material.

For more than 60 years, chemists have approached the study of carbyne by preparing monodisperse conjugated polyynes of ever-increasing lengths.^12−32^ Many of these efforts have sought to define asymptotic limits for physical properties capable of providing insight into the macromolecule. In addition, there is the purely synthetic challenge of the length of an sp carbon chain that can be accessed. Are limitations more a function of the methodologies developed to date or the intrinsic stabilities of the targets?

**Figure 1:**
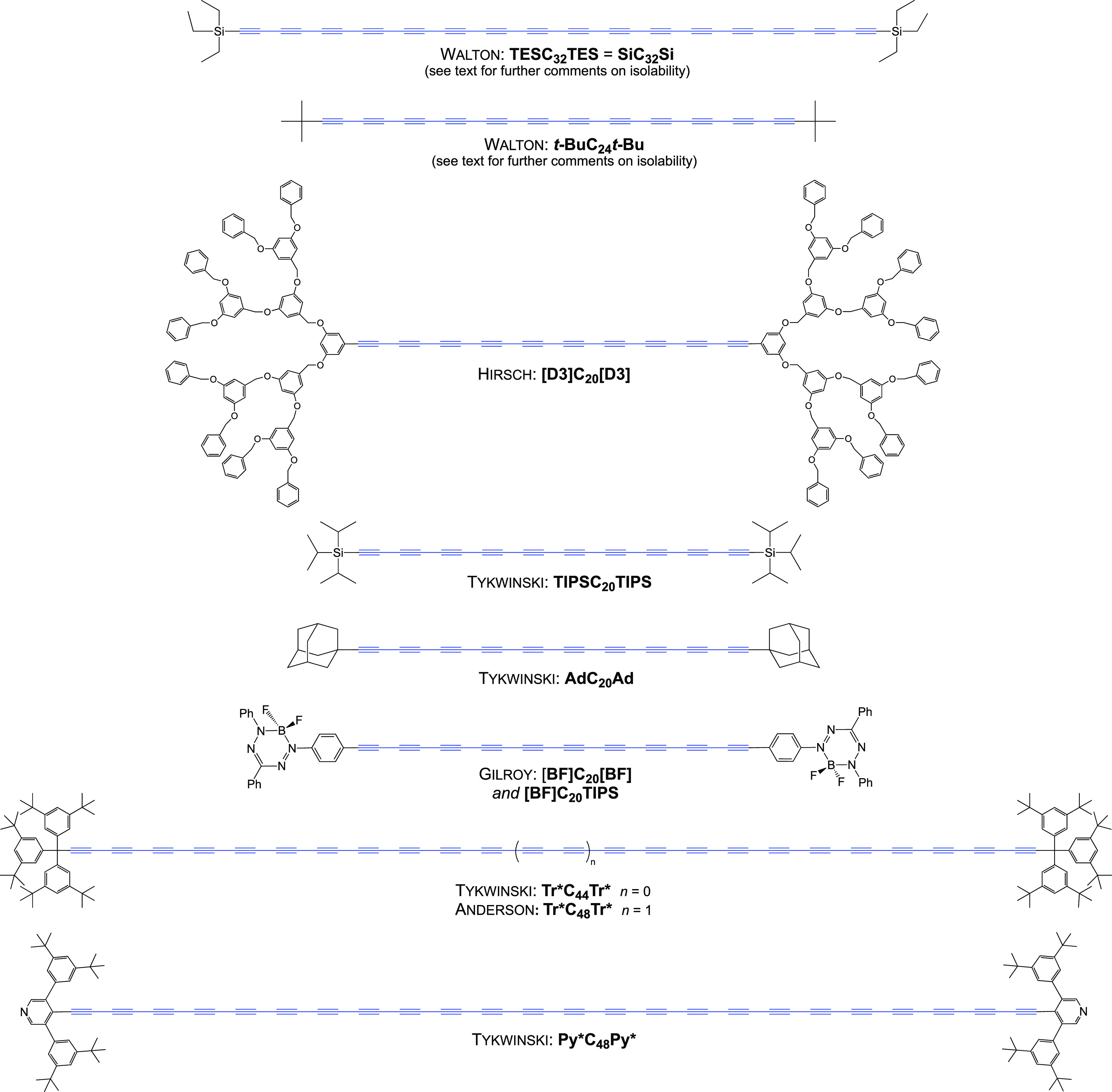
Polyynes with 10 or more triple bonds bearing stabilizing organic or trialkylsilyl endgroups that have been isolated in pure form except where noted.

**Figure 2:**
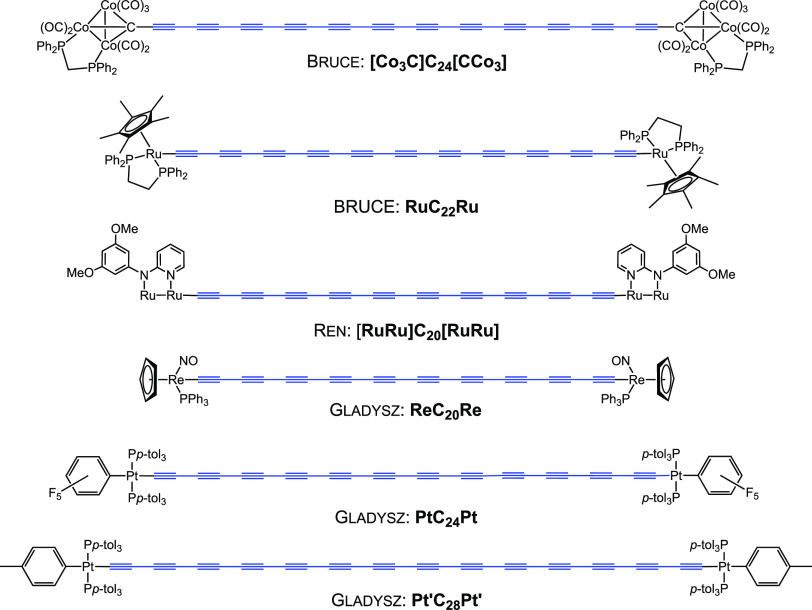
Polyynes with 10 or more triple bonds bearing stabilizing metal endgroups that have been isolated in pure form.

Figures 1 and 2 depict the longest members of series of conjugated polyynes that reach at least 20 sp carbon atoms (eicosadecaynes or higher), have been extensively characterized, and are in nearly all cases isolable in pure form. The first features carbon or silicon endgroups, and the second features transition-metal endgroups. The latter can also be viewed as one of several types of metalated carbynes, a growing class of materials.^33^ There has been a school of thought that bulkier and more electropositive endgroups enhance stabilities, and the former design element is particularly evident in **[D3]C**_**20**_**[D3]**, Tr*C_***x***_Tr* (*x* = 44, 48), and Py*C_**48**_Py*. Steric protection is likely a stabilizing factor in the very long sp carbon chains that have been sequestered within carbon nanotubes^34^ as well as in supramolecular systems in which C_68_ segments are shielded in rotaxanes.^30^

The systems of Walton in Figure 1 are emblematic of the “bronze age” of polyyne synthesis.^13,14^ In the triethylsilyl series, **SiC**_**20**_**Si**, **SiC**_**24**_**Si**, and **SiC**_**32**_**Si** were not fully purified and only characterized by UV–visible spectroscopy.^13^ Samples of *t***-BuC**_**24**_*t***-Bu**, which can be isolated as red needles that decompose within minutes at room temperature, have been similarly characterized,^14^ but pure samples of *t***-BuC**_**20**_*t***-Bu** have been reported.^15,16^ The remaining compounds in Figures 1 and 2 were isolated in pure form and characterized by a full array of modern spectroscopies. The longest carbyne models realized to date have been reported by Tykwinski and Anderson and include (1) C_44_ and C_48_ systems with “supertrityl” endgroups (Tr*) featuring two *meta* disposed *t*-butyl groups on each aryl ring^21,30^ and (2) a C_48_ system with a diarylated *C*-pyridyl endgroup (Py*) with *meta* disposed *t*-butyl groups on each aryl ring.^22^

In this article, we present a full account of our results with compounds with pentafluorophenylplatinum endgroups, *trans*,*trans*-(C_6_F_5_)(*p*-tol_3_P)_2_Pt(C≡C)_*n*_Pt(P*p*-tol_3_)_2_(C_6_F_5_) (**PtC**_***x***_**Pt**, *x* = 2*n*). As shown by **Pt′C**_**28**_**Pt′** in Figure 2, we have also investigated analogs with *p*-tolylplatinum endgroups.^28^ However, this thrust has been paused due to the perception of incrementally higher crystallinities in the pentafluorophenyl complexes, which are capable of attractive C_6_H_4_CH_3_/C_6_F_5_^35^ π-stacking interactions.

**Scheme 1:**
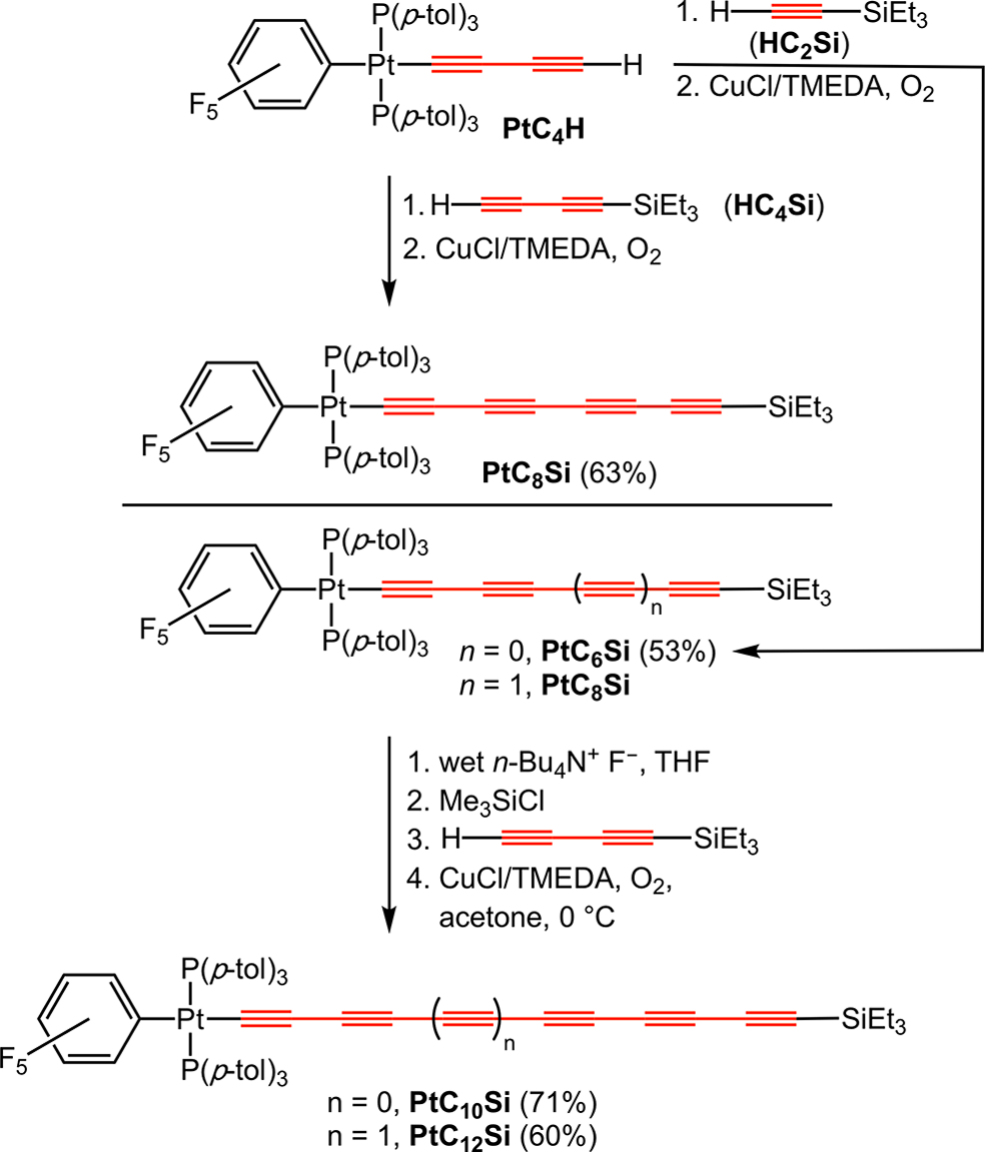
Syntheses of the Key Starting Materials **PtC**_**8**_**Si**, **PtC**_**10**_**Si**, and **PtC**_**12**_**Si**

The triethylsilylpolyynyl building blocks *trans*-(C_6_F_5_)(*p*-tol_3_P)_2_Pt(C≡C)_4_SiEt_3_ (**PtC**_**8**_**Si**),^36−38^**PtC**_**10**_**Si**,^38^ and **PtC**_**12**_**Si**(38) provide the starting point for this study. As reviewed in Scheme 1, their syntheses entail oxidative heterocouplings of **PtC**_**4**_**H**, **PtC**_**6**_**H**, or **PtC**_**8**_**H** (the last two generated *in situ* from **PtC**_***x***_**Si**) with excess HC≡CSiEt_3_ (**HC**_**2**_**Si**) or **HC**_**4**_**Si**.^28^ We commonly employ Hay conditions (O_2_, cat. CuCl/TMEDA),^39^ although many different alkyne coupling recipes have been applied in the syntheses underlying Figures 1 and 2. Some of the sequences in Scheme 1 have also been conducted with the “TIPS” analog, H(C≡C)_2_Si(*i*-Pr)_3_.^36^ While this functions well, no advantages have been found.

Analogous four-carbon sp chain extensions lead to **PtC**_**14**_**Si**, **PtC**_**16**_**Si**, **PtC**_**18**_**Si**, and **PtC**_**20**_**Si**,^38^ and a previously unreported conversion of **PtC**_**18**_**Si** to **PtC**_**22**_**Si** is described in the Supporting Information (SI). Importantly, coupling becomes more rapid as the sp chains are extended, as both initial protodesilylation and the oxidation itself are accelerated.^28,29^ These trends are likely connected to the well-documented increases in the Brønsted acidities of terminal polyynes R(C≡C)_*n*_H as the sp carbon chains lengthen.^40^

With the aid of new building blocks as described below, it has proved possible to extend our earlier report of **PtC**_**24**_**Pt**(36) in four-carbon increments to the dopentacontahexacosayne **PtC**_**52**_**Pt**. This series of complexes has been characterized by all classical spectroscopies, with **PtC**_**52**_**Pt** representing, at least for the moment, the longest monodisperse polyyne with respect to the prior art in Figures 1 and 2. Importantly, these materials continue to show impressive stabilities when purified, suggesting that homologues with still longer sp chains will be accessible.

## Results

### Synthesis of H(C≡C)_4_SiEt_3_ (HC_8_Si)

**Scheme 2:**
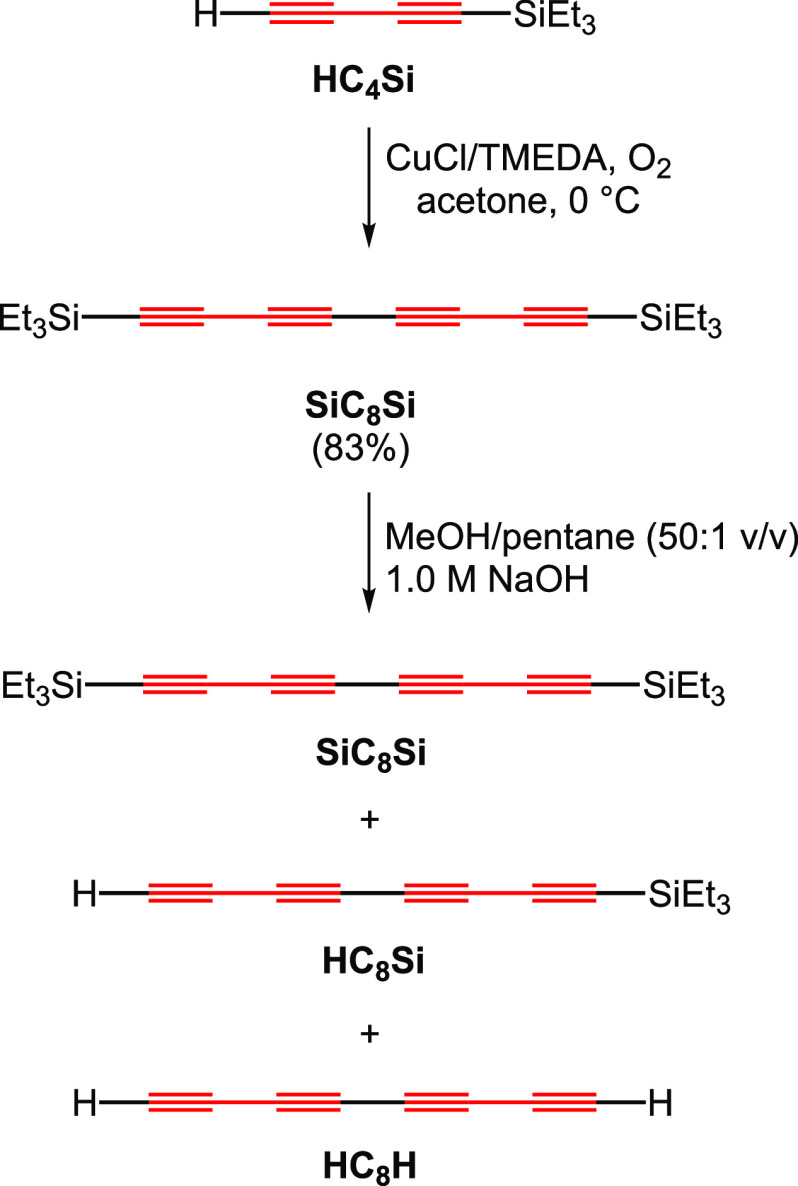
Synthesis of **HC**_**8**_**Si**

In previous syntheses of diplatinum polyynediyl complexes **PtC**_***x***_**Pt**, we employed the two- and four-carbon building blocks **HC**_**2**_**Si** and **HC**_**4**_**Si** for sp chain extensions of monoplatinum precursors **PtC**_***x***_**H** (Scheme 1). For this new effort, the corresponding eight-carbon synthon **HC**_**8**_**Si**, which Walton reported earlier and characterized by UV–visible spectroscopy,^13,14,41^ was generated as depicted in Scheme 2.

First, the Hay homocoupling of **HC**_**4**_**Si** gave **SiC**_**8**_**Si** in 83% yield after workup.^39^ Then a methanol/pentane solution was treated with aqueous NaOH (ca. 11 mol % vs **SiC**_**8**_**Si**). Protodesilylation was not very selective, but **SiC**_**8**_**Si**, **HC**_**8**_**Si**, and **HC**_**8**_**H** could largely be separated by column chromatography (silica gel and hexane) if desired. However, the mixture (designated as “crude **HC**_**8**_**Si**”) was found to give equally good preparative results and was employed in most cases. Pentane or hexane solutions readily decomposed at room temperature or when concentrated and were stored at −78 to −35 °C and used as promptly as possible.

### Oxidative Heterocouplings of **HC_8_Si** and **PtC_8_Si**, **PtC_10_Si**, or **PtC_12_Si**

**Scheme 3:**
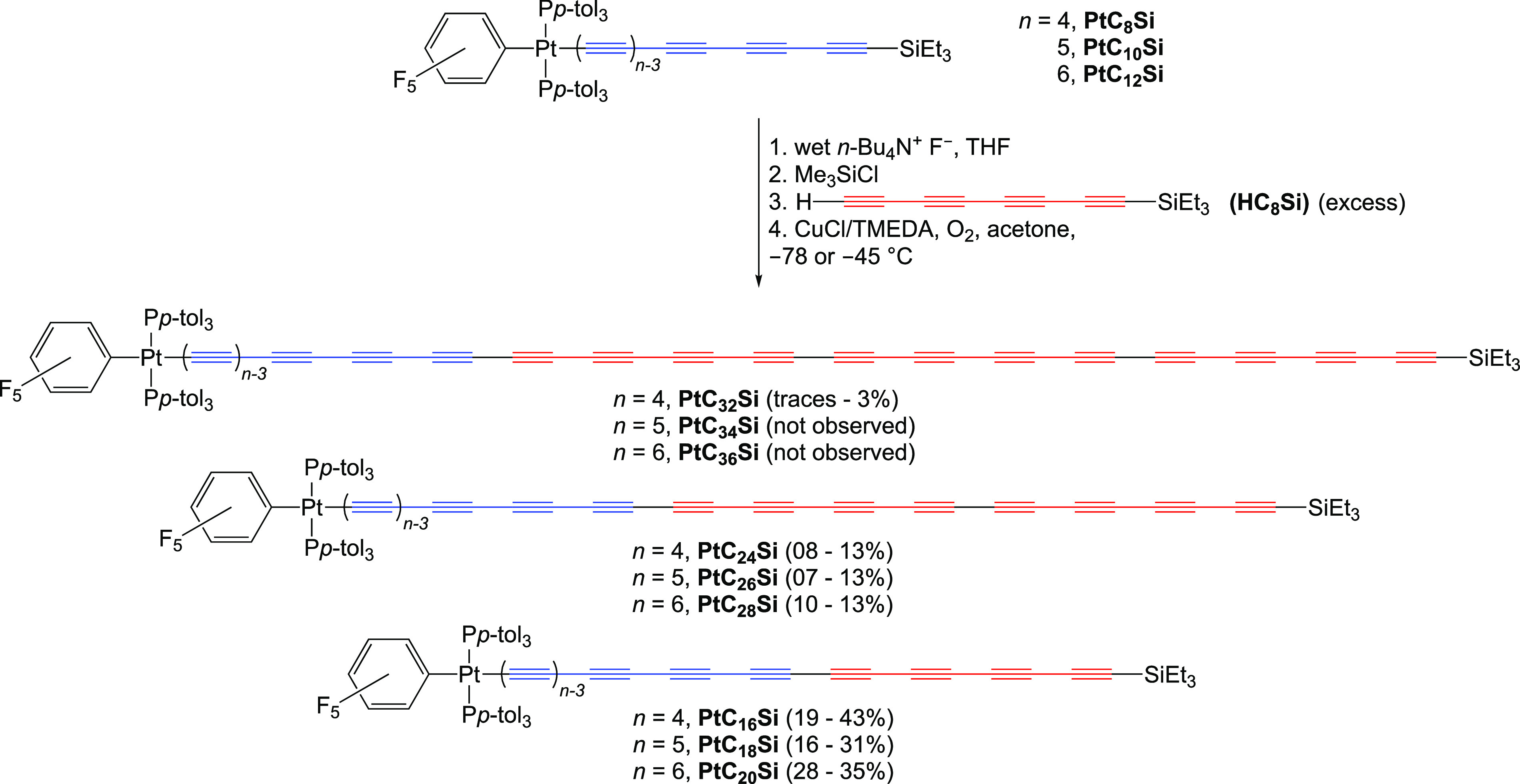
Oxidative Heterocouplings of **PtC**_**8**_**Si**, **PtC**_**10**_**Si**, and **PtC**_**12**_**Si** with **HC**_**8**_**Si**

As shown in Scheme 3, **PtC**_**10**_**Si** and wet *n*-Bu_4_N^+^F^–^ were combined at −78 °C to generate **PtC**_**10**_**H**, as verified by “click” trapping reactions with azides.^38^ Then Me_3_SiCl was added, a step that has often enhanced yields in the **Pt′C**_***x***_**Pt′** series (Figure 2)^29^ and is thought to scavenge fluoride ion. Control experiments without Me_3_SiCl are described below. An excess of crude **HC**_**8**_**Si** was added at −35 °C, followed by Hay conditions. Silica gel chromatography gave the known complex **PtC**_**18**_**Si**(38) and a new faster eluting complex **PtC**_**26**_**Si** in 16–31% and 7–13% yields, respectively.

“Double addition” products analogous to **PtC**_**26**_**Si** have occasionally been observed with the shorter building block **HC**_**4**_**Si**.^29^ The present example likely involves the *in situ* protodesilylation of **PtC**_**18**_**Si** and coupling with another equivalent of **HC**_**8**_**Si**. Protodesilylation becomes progressively more facile as the sp chain is lengthened. However, some participation of **HC**_**8**_**H** cannot be excluded. Continued elution of the column gave lesser amounts of diplatinum complexes **PtC**_***x***_**Pt** (*x* = 20, 28, 36 via **PtC**_**10**_**H**/**PtC**_**18**_**H** homo/heterocoupling), as assayed by MS and HPLC.

Analogous heterocouplings of **PtC**_**12**_**Si** and crude **HC**_**8**_**Si** led to **PtC**_**20**_**Si**(38) and the new complex **PtC**_**28**_**Si** in 28–35% and 10–13% yields, respectively (Scheme 3). In the case of **PtC**_**8**_**Si** and **HC**_**8**_**Si**, three monoplatinum complexes were isolated, **PtC**_**16**_**Si** (19%),^38^**PtC**_**24**_**Si** (8%), and the “triple addition” product **PtC**_**32**_**Si** (traces–3%). Small amounts of diplatinum byproducts formed in all cases.

The complexes **PtC**_**24**_**Si**, **PtC**_**26**_**Si**, **PtC**_**28**_**Si**, and **PtC**_**32**_**Si** were dark-brown-orange to red-violet solids that tolerated brief exposures to air but were strongly discolored after several days. DSC measurements with **PtC**_**24**_**Si** and **PtC**_**26**_**Si** showed exotherms with T_i_ values of 79–93 °C (Table s2).^42^ These and other new complexes below were characterized by microanalyses, NMR (^1^H, ^13^C{^1^H}, ^31^P{^1^H}), IR, UV–visible spectroscopy, and MS. Key ^13^C{^1^H} NMR and UV–visible properties are summarized in Tables 1–4, and other data are collected in the SI. Molecular ions were always observed by MS.

**Table 1:** ^13^C{^1^H} NMR Data (δ/ppm, *J*/Hz, 126 MHz/CDCl_3_) for **PtC**_***x***_**Si**

complex	Pt*C*≡C [^1^*J*_CPt_]	PtC≡*C* [^2^*J*_CPt_]	*C*≡CSi	C≡*C*Si [^1^*J*_CSi_]	others
**PtC**_**6**_**Si**^a^^,^^b^	104.2	95.4	91.2^c^	80.3^c^	66.1, 55.9
**PtC**_**8**_**Si**^a^^,^^b^	106.3 [1000]	95.2 [264]	90.2^c^	82.9^c^ [75.9]	66.6, 64.1, 59.3, 56.3
**PtC**_**10**_**Si**^b^^,^^d^	108.2	95.2 [262]	89.8	84.9 [73.1]	66.8, 65.2, 63.3, 60.5, 59.7, 56.7
**PtC**_**12**_**Si**^b^^,^^d^	109.4	95.1 [261]	89.4	86.2 [73.0]	67.2, 65.5, 64.2, 62.7, 61.4, 60.8, 59.8, 56.7
**PtC**_**14**_**Si**^b^^,^^d^	110.2	95.0	89.2	87.0	67.4, 65.8, 64.4, 63.5, 62.3, 62.0, 61.7, 60.8, 59.8, 56.7
**PtC**_**16**_**Si**^b^^,^^d^	110.7	95.0	89.0	87.6	67.6, 66.2, 64.8, 63.8, 63.2, 62.5, 62.4, 62.0, 61.7, 60.8, 59.7, 56.7
**PtC**_**18**_**Si**^d^	111.0	94.9	88.9	87.9	67.6, 66.4, 65.0, 64.1, 63.4, 62.9, 62.8, 62.4, 61.9, 61.7, 60.7, 59.7, 56.7^e^
**PtC**_**20**_**Si**^d^	111.3	94.9	88.9	88.2	67.8, 66.5, 65.3, 64.4, 63.7, 63.2, 63.1, 63.0, 62.9, 62.7, 62.3, 61.7, 61.6, 60.7, 59.7, 56.7
**PtC**_**22**_**Si**	111.3^f^	94.9	88.8	88.4	67.9, 66.6, 65.4, 64.6, 63.9, 63.43, 63.37, 63.19, 63.17, 62.9, 62.7, 62.6, 62.2, 61.65, 61.56, 60.7, 59.7, 56.7
**PtC**_**24**_**Si**	111.5^f^	94.9	88.8	88.5	67.9, 66.7, 65.5, 64.7, 64.1, 63.63, 63.60, 63.4, 63.3, 63.2, 63.1, 62.8, 62.7, 62.5, 62.2, 61.61, 61.53, 60.7, 59.7, 56.7
**PtC**_**26**_**Si**	111.6^f^	94.9	88.7	88.6	68.0, 66.8, 65.6, 64.8, 64.3, 63.8, 63.7, 63.6, 63.42, 63.39, 63.3, 63.04, 63.0, 62.8, 62.7, 62.4, 62.1, 61.6, 61.5, 60.6, 59.7, 56.7
**PtC**_**28**_**Si**	–g	94.9	88.8	88.4	67.7, 66.6, 65.4, 64.7, 64.1, 63.7, 63.6, 63.5, 63.4, 63.26, 63.23, 63.08, 63.03, 62.8, 62.42, 62.39, 62.2, 61.9, 61.36, 61.30, 60.4, 59.5, 56.5^e^
**PtC**_**32**_**Si**	–g	94.7	89.2	88.9	68.0, 66.9, 65.6, 65.0, 64.5, 64.1, 63.8, 63.5, 63.1, 62.9, 62.5, 62.0, 61.52, 61.47, 60.6, 59.6, 58.7, 56.6^h^

a Data at 101 MHz are from ref (29).

b The chemical shifts of the TIPS analogs are very similar, as reported in ref (36) or (38).

c This represents a reversal from the assignment in ref (29), as explained in ref (36) (see closing portion of that discussion section).

d Data at 126 MHz are from ref (38).

e One fewer than the theoretical number of *sp* carbon atom signals is observed, presumably due to the overlapping of two signals; that at 62.8 ppm is more intense than the others.

f Poor signal-to-noise ratio.

g Signal was not observed.

h Only 18 distinct signals were observed (theory: 28).

**Table 2:** ^13^C{^1^H} NMR Data (δ/ppm, *J*/Hz, 126 MHz/CDCl_3_) for **PtC**_***x***_**Pt**

complex	PtC≡C [^1^*J*_CPt_]	PtC≡C [^2^*J*_CPt_]	others
**PtC**_**4**_**Pt**^a^	86.4 [970]	104.0 [262]	–
**PtC**_**6**_**Pt**^a^	95.8	98.4	61.1
**PtC**_**8**_**Pt**^a^	100.6 [998]	96.7 [265]	64.1, 58.1
**PtC**_**12**_**Pt**^a^	106.5	95.5	65.7, 63.0, 61.0, 57.1
**PtC**_**16**_**Pt**^a^	109.1	95.0	66.7, 64.9, 63.1, 61.5, 60.1, 56.8
**PtC**_**20**_**Pt**^b^	110.5	95.0	67.6, 65.9, 64.5, 63.4, 62.3, 61.1, 59.9, 56.8
**PtC**_**24**_**Pt**^b^	111.2	94.9	67.8, 66.4, 65.1, 64.2, 63.4, 62.7, 61.8, 60.8, 59.8, 56.7
**PtC**_**28**_**Pt**	111.5^c^	94.9	67.9, 66.7, 65.4, 64.6, 63.9, 63.5, 62.9, 62.3, 61.6, 60.7, 59.7, 56.7
**PtC**_**32**_**Pt**	111.4^c^	94.7	67.7, 66.5, 65.4, 64.6, 64.1, 63.6, 63.3, 62.9, 62.5, 62.0, 61.3, 60.5, 59.5, 56.5
**PtC**_**36**_**Pt**	111.6^c^	94.9	67.9, 66.8, 65.7, 64.9, 64.4, 64.1, 63.8, 63.5, 63.2, 62.9, 62.6, 62.1, 61.5, 60.6, 59.6, 56.6
**PtC**_**40**_**Pt**	111.5^c^	94.8	68.0, 66.9, 65.7, 65.0, 64.5, 64.2, 63.9, 63.7, 63.5, 63.3, 63.1, 62.9, 62.5, 62.1, 61.5, 60.6, 59.6, 56.6
**PtC**_**44**_**Pt**	–d	94.8	68.0, 67.4, 66.9, 65.7, 65.0, 64.5, 64.2, 63.9, 63.8, 63.6, 63.4, 63.3, 63.0, 62.8, 62.5, 62.0, 61.5, 60.6, 59.6, 56.6
**PtC**_**52**_**Pt**^e^	111.0^c^	95.5	68.0, 67.8, 66.8, 66.6, 65.5, 65.2, 64.6, 64.2, 63.9, 63.3, 62.8, 62.6, 62.2, 61.7, 61.5, 60.8, 60.6, 59.9, 59.8, 57.3, 57.3^e^

a Data at 101 MHz are from ref (29).

b Data at 126 MHz are from ref (36).

c Poor signal-to-noise ratio.

d Signal was not observed.

e For solubility purposes, this spectrum was recorded in 1:1 v/v C_6_D_5_CD_3_/CDCl_3_; only 23 signals were observed (theory: 26); that at 62.0 ppm was more intense than the others (Figure 4).

**Table 3:** UV–Visible Data for **PtC**_***x***_**Si** (CH_2_Cl_2_)

complex	wavelength (nm) [ε (M^–1^ cm^–1^)]	concentration (mol/L)
**PtC**_**6**_**Si**^a^	244 [116000], 249 [125000], 255 [131000], 261 [104000]	1.25 × 10^–6^
**PtC**_**8**_**Si**^a^	255 [62000], 269 [65000], 287 [98000], 310 [73000]	1.25 × 10^–6^
**PtC**_**10**_**Si**^b^	259 [51200], 272 [54200], 287 [68600], 302 [125000], 312 [92700], 329 [186000], 378 [1900], 409 [1400], 444 [940]	4.24 × 10^–6^
**PtC**_**12**_**Si**^b^	268 [92900], 282 [71200], 298 [85100], 313 [105000], 334 [139000], 350 [164000], 401 [1200], 437 [730], 475 [240]	4.10 × 10^–6^
**PtC**_**14**_**Si**^b^	272 [82500], 287 [76800], 302 [95200], 319 [110000], 337 [143000], 357 [188000], 373 [151000], 429 [1700], 462 [1500], 504 [1100]	4.75 × 10^–6^
**PtC**_**16**_**Si**^b^	261 [60600], 274 [59600], 289 [67700], 304 [91000], 320 [115000], 338 [134000], 358 [175000], 378 [188000], 393 [127000]	2.41 × 10^–6^
**PtC**_**18**_**Si**^b^	275 [46400], 289 [53600], 304 [76300], 319 [105000], 336 [124000], 355 [154000], 376 [190000], 396 [192000], 418 [112000]^c^	6.49 × 10^–6^
**PtC**_**20**_**Si**^b^	319 [90700], 333 [121000], 350 [144000], 370 [185000], 392 [216000], 411 [203000], 435 [109000]^c^	4.18 × 10^–6^
**PtC**_**22**_**Si**	290 [61500], 305 [77200], 320 [103000], 336 [129000], 352 [148000], 372 [174000], 393 [203000], 407 [207000]	5.84 × 10^–6^
**PtC**_**24**_**Si**	288 [45600], 338 [115000], 355 [151000], 374 [178000], 395 [221000], 418 [233000], 433 [205000], 462 [99400]^c^	2.20 × 10^–6^
**PtC**_**26**_**Si**	348 [122000], 365 [160000], 384 [186000], 404 [224000], 429 [229000], 448 [90800]^c^, 503 [19200]^c^	4.90 × 10^–6^
**PtC**_**28**_**Si**	362 [142000], 380 [189000], 399 [218000], 420 [241000], 444 [223000], 463 [169000]^c^, 490 [65700]^c^	3.70 × 10^–6^
**PtC**_**32**_**Si**^d^	259 [153000], 374 [222000], 394 [262000], 422 [283000]	3.52 × 10^–6^

a Data are from ref (29).

b Data are from ref (38).

c Shoulder; the wavelength was determined from the second derivative of the spectrum, with the extinction coefficients taken from the raw data in Figure 6.

d Due to the limited amount of sample, the ε values are considered to be accurate to only two significant digits.

**Table 4:** UV–Visible Data for **PtC**_***x***_**Pt** (CH_2_Cl_2_)

complex	wavelength (nm) [ε (M^–1^ cm^–1^)]	concentration (mol/L)
**PtC**_**4**_**Pt**^a^	330 [17000], 350 [13200]	1.25 × 10^–5^
**PtC**_**6**_**Pt**^a^	315 [44000], 345 [15000], 358 [11000], 369 [9000]	1.25 × 10^–5^
**PtC**_**8**_**Pt**^a^	294 [88 000], 326 [126000], 356 [7000], 383 [6000], 414 [3000]	1.25 × 10^–6^
**PtC**_**10**_**Pt**^b^	291 [44000], 331 [54000], 362 [78000], 382 [126000]	2.25 × 10^–6^
**PtC**_**12**_**Pt**^a^	315 [101000], 336 [267000], 359 [432000]	1.25 × 10^–6^
**PtC**_**14**_**Pt**^b^	298 [40000], 320 [51000], 338 [146000], 360 [392000], 388 [550000]	2.25 × 10^–6^
**PtC**_**16**_**Pt**^a^	290 [46000], 306 [42000], 326 [54000], 346 [151000], 369 [397000], 397 [602000]	1.25 × 10^–6^
**PtC**_**18**_**Pt**^b^	293 [59500], 308 [59600], 324 [55000], 341 [62000], 360 [141000], 384 [326000], 413 [446000]	2.25 × 10^–6^
**PtC**_**20**_**Pt**^c^	293 [55000], 309 [58200], 325 [60400], 346 [65000], 372 [117000], 398 [248000], 428 [326000]	2.25 × 10^–6^
**PtC**_**24**_**Pt**^c^	307 [105000], 321 [130000], 336 [144000], 353 [157000], 372 [171000], 391 [252000], 421 [430000], 452 [516000]	2.25 × 10^–6^
**PtC**_**28**_**Pt**	327 [105000], 341 [115000], 358 [125000], 435 [290000], 467 [303000]	1.95 × 10^–6^
**PtC**_**32**_**Pt**	374 [162000], 445 [329000], 477 [331000]	2.21 × 10^–6^
**PtC**_**36**_**Pt**	388 [205000], 453 [358000], 483 [353000]	1.36 × 10^–6^
**PtC**_**40**_**Pt**	301 [86500], 418 [296000], 456 [378000], 486 [369000]	2.09 × 10^–6^
**PtC**_**44**_**Pt**	320 [89400], 429 [312000], 458 [387000], 491 [376000]	2.35 × 10^–6^
**PtC**_**48**_**Pt**^d^	320 [108000], 425 [338000], 451 [422000], 489 [389000]	1.01 × 10^–6^
**PtC**_**52**_**Pt**^d^	324 [103000], 425 [327000], 454 [412000], 490 [397000]	1.32 × 10^–6^

a Data are from ref (29).

b These complexes have been prepared as part of a separate study and will be reported at a later date.

c This represents new data from that reported in ref (36); the ε values are viewed as more accurate.

d Due to the limited amount of sample, the ε values are considered to be accurate to only two significant digits.

These complexes represent the most extensive series of unsymmetrically substituted polyynes in the literature, crowned by dotriacontahexadecayne **PtC**_**32**_**Si**. While they are less suitable for some of the goals in the Introduction, there are many trends of interest as noted below. To spare researchers from combing through earlier papers to retrieve data, all tables have been “backfilled” with lower homologues. Of course, some properties, such as the ^1^H NMR chemical shifts of the −C_6_H_4_C*H*_3_ signals (Table s3), are essentially “flat” throughout the series.

### Oxidative Homocouplings

**Scheme 4:**
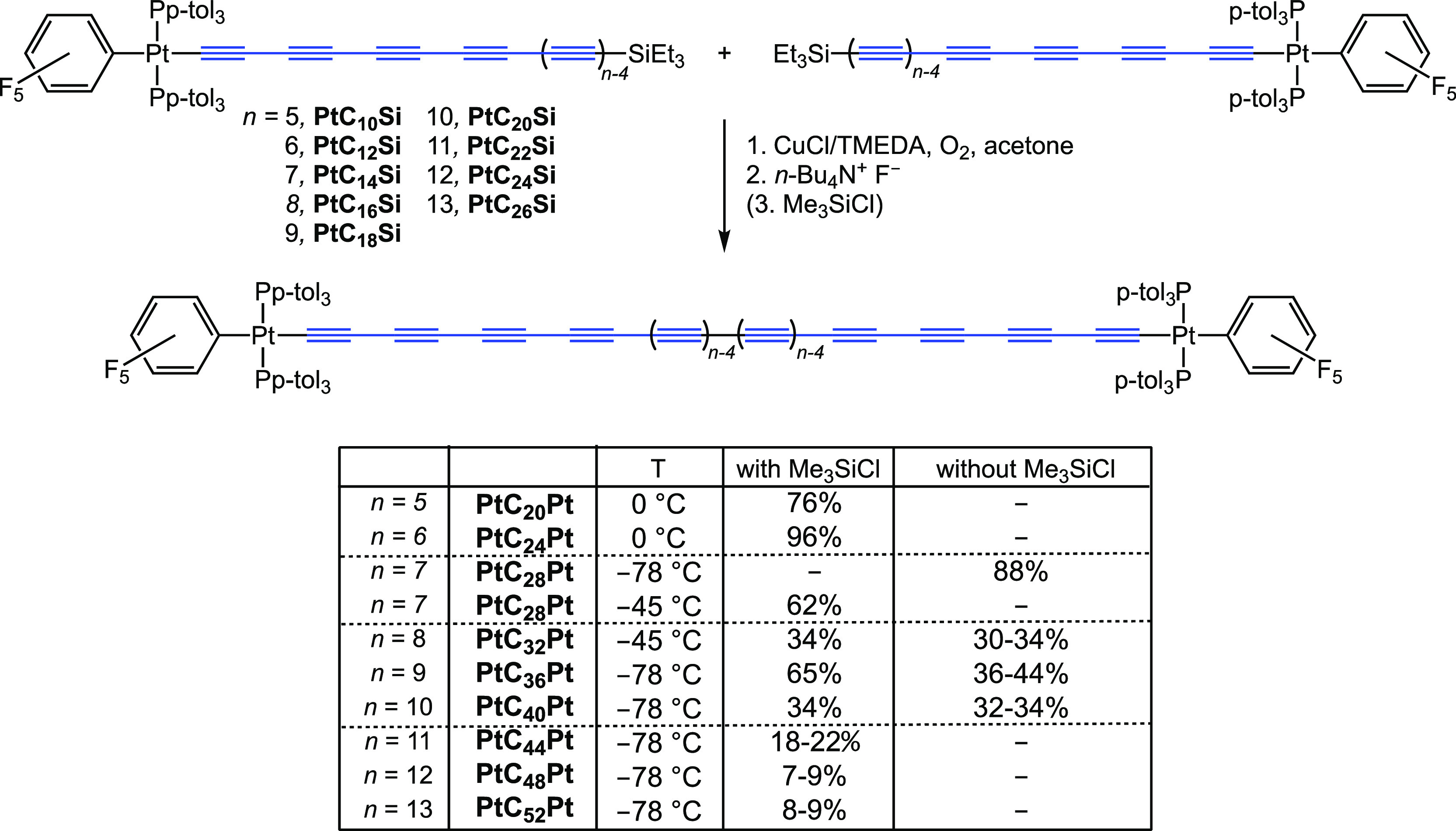
Oxidative Homocouplings of **PtC**_*x*_**H** Generated from **PtC**_*x*_**Si**

Although syntheses of **PtC**_**20**_**Pt** and **PtC**_**24**_**Pt** have been reported, these utilized educts with Pt(C≡C)_*n*_Si(*i*-Pr)_3_ linkages.^36^ The preparative data in Scheme 4 are restricted to the triethylsilyl precursors **PtC**_***x***_**Si**, and details for **PtC**_**20**_**Pt** and **PtC**_**24**_**Pt** are given in the SI. In all cases, **PtC**_***x***_**Si** was first combined with O_2_ and the Hay catalyst so that upon subsequent addition of wet *n*-Bu_4_N^+^F^–^ the resulting **PtC**_***x***_**H** would have a better chance of undergoing oxidation as opposed to competing processes.^43^ In this context, click trapping reactions have not yet been explored beyond **PtC**_**18**_**H**.^38^

As shown in Scheme 4, reactions of **PtC**_**10**_**Si** and **PtC**_**12**_**Si** at 0 °C gave **PtC**_**20**_**Pt** and **PtC**_**24**_**Pt** in 76–96% yields after silica gel chromatography. Due to the protodesilylation and oxidation rate trends noted above, couplings of higher homologues could be carried out at −45 to −78 °C. The addition of wet *n*-Bu_4_N^+^ F^–^ subsequent to O_2_ and CuCl/TMEDA called into question the actual need for Me_3_SiCl. Good yields of **PtC**_**28**_**Pt** could be obtained with or without them (88–62%, Scheme 4). However, for **PtC**_**32**_**Pt**, **PtC**_**36**_**Pt**, and **PtC**_**40**_**Pt**, better yields were realized with Me_3_SiCl (65–34% vs 44–30%). The solubility of **PtC**_**40**_**Pt** was noticeably lower in hexanes but remained appreciable in CH_2_Cl_2_ and CHCl_3_.

Several groups have found that when Hay conditions are used in the syntheses of polyynes in Figures 1 and 2, byproducts arising from the loss of C_2_ can form.^16−18,19b,21,22,29,38^ This occurs sporadically and so far has no mechanistic explanation. This problem was encountered in our first attempts to prepare **PtC**_**44**_**Pt** and **PtC**_**48**_**Pt**, as assayed by MS data that showed M^+^, M^+^–24, and M^+^–48 ions and HPLC. In second-generation efforts, we have been careful to check the precursors **PtC**_***x***_**Si** for such ions.^44^ When absent, the homocoupling products have not so far contained any lower homologues. Since MS is normally not a quantitative technique and lower homologues derived from C_2_ loss should be more volatile, we have not interpreted the relative ion intensities of samples.

Regardless, the yields drop to 22–7% for **PtC**_**44**_**Pt** and higher homologues (Scheme 4). When the solvents were removed from crude samples and the residues were analyzed by MS, no ions suggestive of dimeric or other nonoligomeric byproducts were observed. No other materials were eluted from silica gel. Complexes **PtC**_**40**_**Pt** to **PtC**_**52**_**Pt** were dark-red solids with decomposition points of >70 °C (Table s2), and photographs of CH_2_Cl_2_ solutions are provided in Figure s1. In the earlier stages of this work, some variability in sample stability was noted, but this is now believed to be a function of purity. For **PtC**_**32**_**Pt** and higher homologues, it is advisible to shield samples from light, but in no case does rapid degradation occur. They are best stored at −35 °C and used within 1 week, although many samples have survived much longer.

### NMR Spectra

The ^13^C{^1^H} NMR data in Tables 1 and 2 exhibit several conspicuous features. The chemical shifts of the PtC≡C signals shift downfield with increasing chain lengths (**PtC**_***x***_**Si**, 104.2 to 111.6 (Δδ 7.4 ppm), *x* = 6–26; **PtC**_***x***_**Pt**, 86.4 to 111.5 (Δδ 25.1 ppm), *x* = 4–40). Plots in Figure s2 illustrate the asymptotic approach to the macromolecular limit of **PtC**_**∞**_**Si** and **PtC**_**∞**_**Pt**, but these are obvious from the raw numbers alone (∼111.6 ppm in both cases). The PtC≡C signals of **PtC**_***x***_**Si** vary only from 95.4 to 94.7 ppm (Δδ < 1 ppm), but those of **PtC**_***x***_**Pt** are better differentiated (Δδ 9.2 ppm). Both series converge to the same 94.7 ppm value. The C≡CSi signals of **PtC**_***x***_**Si** shift downfield with increasing chain length (80.3 to 88.9 or Δδ 8.6 ppm), whereas the C≡CSi signals shift slightly upfield (91.2 to 88.8 or Δδ 2.4 ppm) to essentially the same limit.

**Figure 3:**
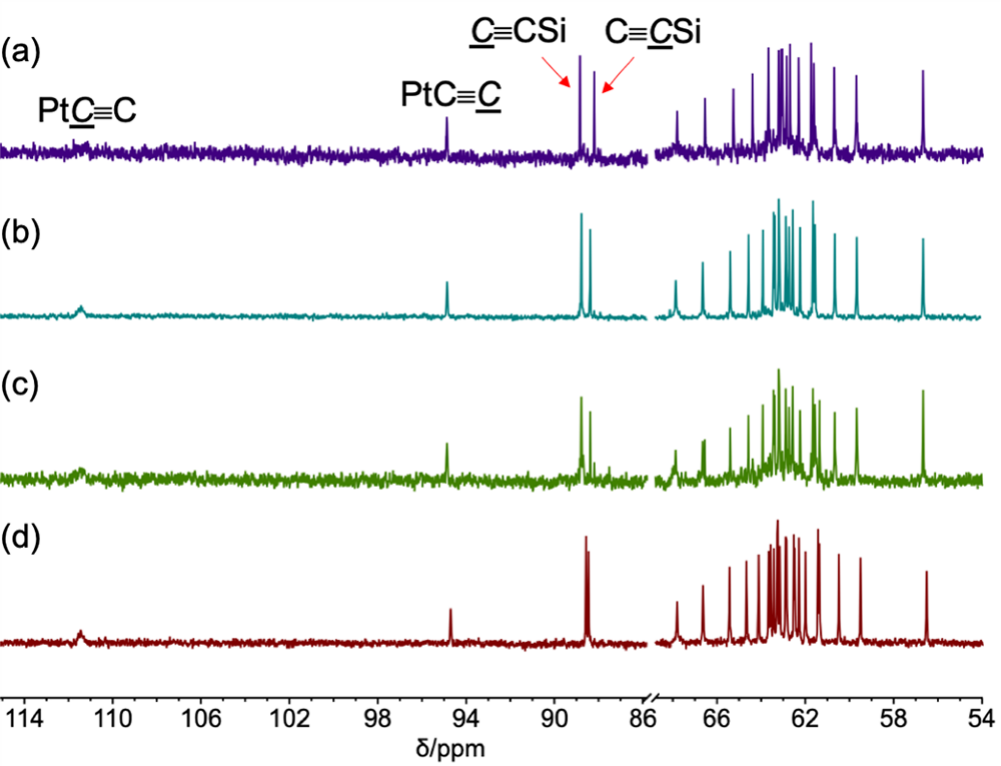
Representative ^13^C{^1^H} NMR spectra of **PtC**_*x*_**Si** (CDCl_3_, 126 MHz, *sp* carbon signals only): (a) **PtC**_**20**_**Si**, (b) **PtC**_**22**_**Si**, (c) **PtC**_**24**_**Si**, and (d) **PtC**_**26**_**Si**.

**Figure 4:**
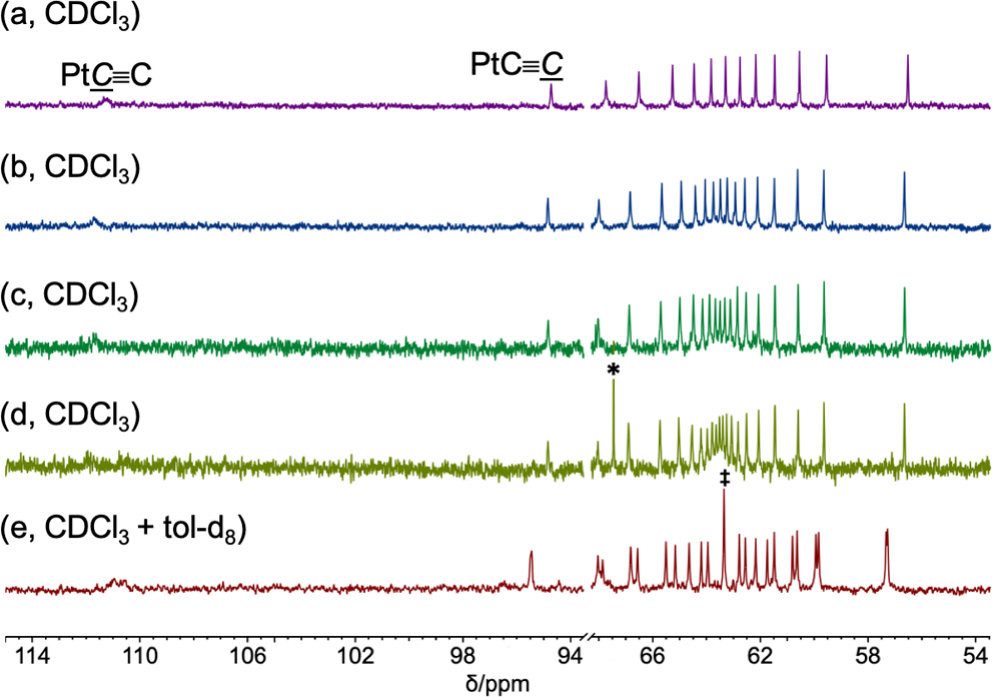
Representative ^13^C{^1^H} NMR spectra of **PtC**_*x*_**Pt** (126 MHz, *sp* carbon signals only): (a) **PtC**_**28**_**Pt**, (b) **PtC**_**36**_**Pt**, (c) **PtC**_**40**_**Pt**, (d) **PtC_44_Pt**, and (e) **PtC**_**52**_**Pt**. The signal * is a solvent-based impurity, and ‡ represents 2 or more carbon atoms (23 signals total; theory: 26).

As illustrated in Figures 3 and 4, the intensities of the PtC≡C signals are diminished relative to those of other sp carbon atoms by couplings to ^31^P and ^195^Pt (33.8% natural abundance, *I* = 1/2). These usually remain unresolved.^45^ With **PtC**_**52**_**Pt**, default solvent CDCl_3_ had to be augmented with toluene-*d*_8_ to enhance the solubility and visualize the sp signals. In Figure 3, the C≡CSi signals remain quite intense, despite couplings to ^29^Si (4.7% natural abundance).^46^ All of these splittings are evident in an optimized ^13^C{^1^H} NMR spectrum of a close analog of **PtC**_**8**_**Si**.^36^ Furthermore, DFT computations on TIPS analogs of **PtC**_***x***_**Si** (*x* = 8, 12, 16) nicely reproduce the chemical shift trends.^36^

As shown in Tables 1 and 2 and Figure 4 and in general agreement with the other polyynes in Figures 1 and 2,^31b^ the remaining *C*≡*C* signals cluster between 55.9 and 68.2 ppm. Further assignments are challenging.^45^ It might be simplistically postulated that those furthest upfield correspond to the innermost carbon atoms, but labeling and DFT studies have disproven this conjecture.^18,36^ To aid additional analyses below, the average chemical shifts of all sp carbon atoms except those of the PtC≡C and C≡CSi linkages (e.g., PtC≡C(*C≡C*)_*n*−2_C≡CSi/Pt) are compiled in Figure s3.

As presented in Table s1, the ^31^P{^1^H} NMR chemical shifts of **PtC**_***x***_**Si** and **PtC**_***x***_**Pt** fall into the same narrow range (17.90–18.05 and 17.20–18.04 ppm for *x* ≥ 6), with ^1^*J*_PPt_ decreasing approximately monotonically from 2636 to ∼2600 Hz and 2713 to ∼2600 Hz. With the *para*-substituted arylplatinum bis(phosphine) complexes *trans*-(*p*-ZC_6_H_4_)Pt(PEt_3_)_2_X (X = Br, H), the ^1^*J*_PPt_ values decrease as the Z groups become more electron-withdrawing.^47^ This provides further support for the enhanced electronegativities and ≡CH acidities of longer C_*x*_ chains.

### Vibrational Spectroscopy

The IR ν_C≡C_ bands of **PtC**_***x***_**Si** and **PtC**_***x***_**Pt** (*x* ≥ 6) are summarized in Table s1. Through *x* = 26–28, the number of absorptions generally increases with sp chain length, in accord with computational studies of **HC**_***x***_**H**.^48^ Furthermore, at the same sp chain length, unsymmetrically substituted **PtC**_***x***_**Si** complexes generally exhibit more bands than **PtC**_***x***_**Pt**. This is consistent with their nonzero dipole moment along the platinum–silicon vector and inherently noncentrosymmetric nature, which removes the IR/Raman mutual exclusion rule formally applicable to **PtC**_***x***_**Pt**.^19a^

**Figure 5:**
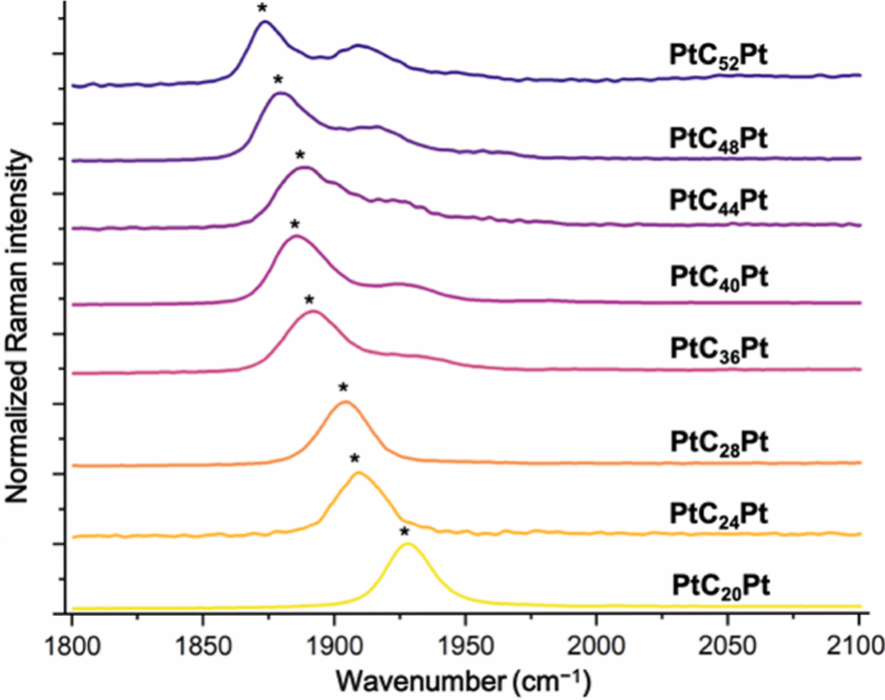
Raman spectra of **PtC**_*x*_**Pt** (∼1 mM solution in CH_2_Cl_2_, Я band denoted by *).

Raman spectra of polyynes have received attention as probes of BLA, as elaborated in the Discussion section.^49^ Although multiple Raman ν_C≡C_ absorptions are often detected^27^ and/or predicted computationally,^11^ spectra are normally dominated by the so-called Я band, corresponding to a collective *C*≡*C* stretching and ≡*C*–*C*≡ contraction mode. Thus, Raman spectra were acquired in CH_2_Cl_2_ as described in the Experimental Section. Data are summarized in Figure 5, Table 5, and Figure s6 and interpreted below.

**Table 5:** Raman Data for **PtC**_***x***_**Pt** (ν_C≡C_, cm^–1^, ∼1 mM CH_2_Cl_2_ Solutions)

complex	Я band	other bands, 1800–2200 cm^–1^
**PtC**_**6**_**Pt**	2054 (w)^a^	2179 (s)
**PtC**_**8**_**Pt**	2053 (w)^a^	2179 (s)
**PtC**_**12**_**Pt**	2040 (w)^a^	2179 (s)
**PtC**_**20**_**Pt**	1928 (s)	–
**PtC**_**24**_**Pt**	1910 (s)	–
**PtC**_**28**_**Pt**	1904 (s)	–
**PtC**_**36**_**Pt**	1891 (s)	–
**PtC**_**40**_**Pt**	1886 (s)	1930 (w)
**PtC**_**44**_**Pt**	1887 (s)	1930 (w)
**PtC**_**48**_**Pt**	1889 (s)	1925 (w)
**PtC**_**52**_**Pt**	1880 (s)	1924 (m)

a Due to interfering fluorescence, these bands broadened and the values are approximate.

### Optical Properties

**Figure 6:**
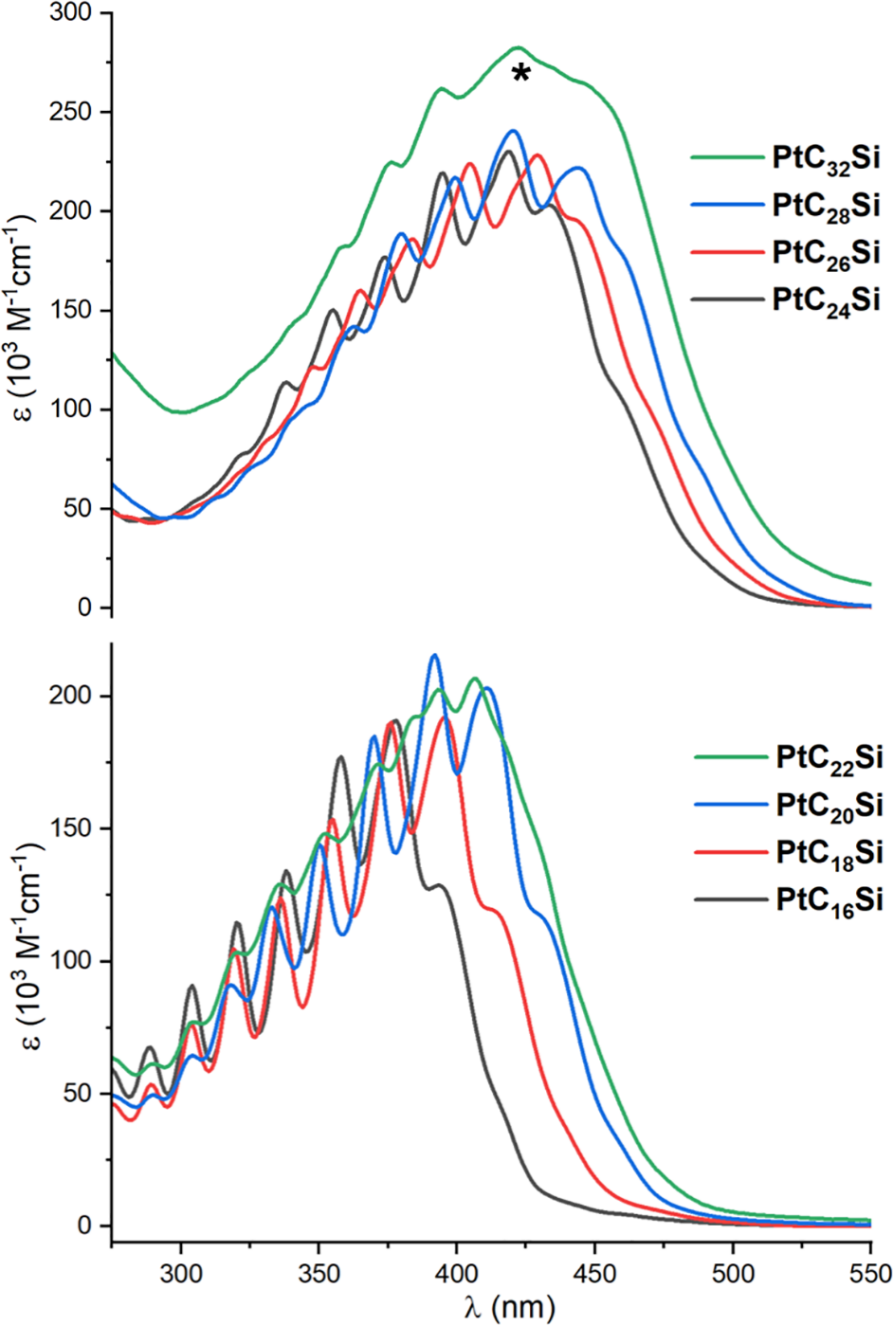
UV–visible spectra of 2.20 × 10^–6^–6.49 × 10^–6^ M CH_2_Cl_2_ solutions of **PtC**_*x*_**Si**. Due to the quantity of **PtC**_**32**_**Si** available, the extinction coefficients for this trace (*) are less reliable.

**Figure 7:**
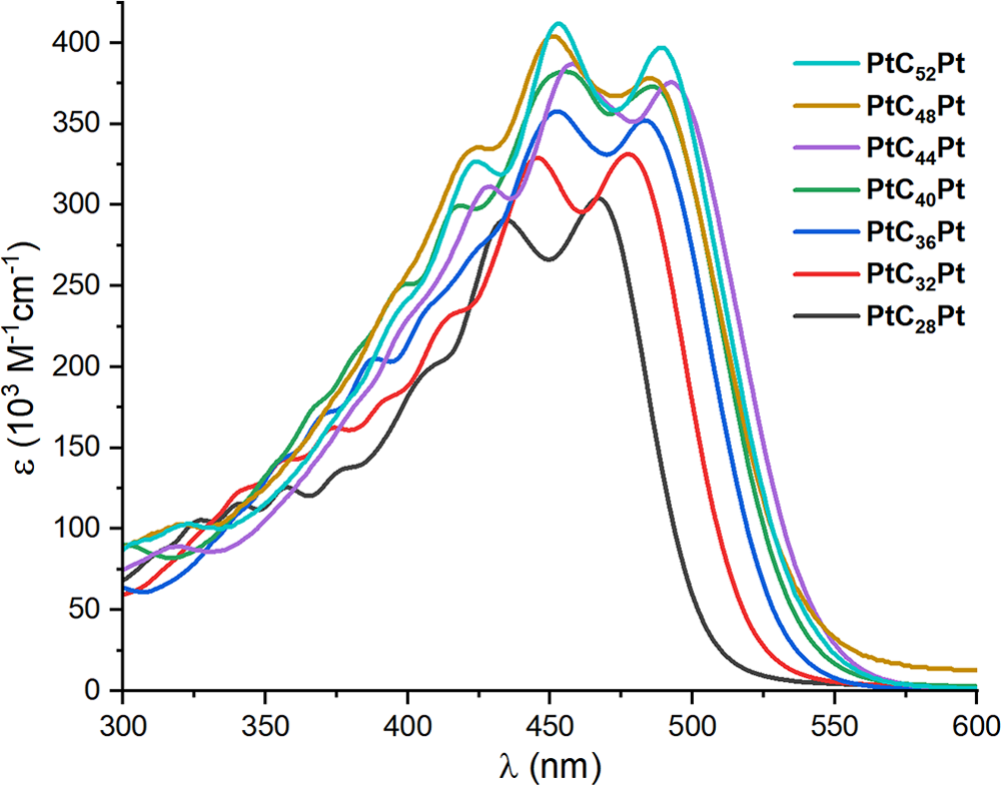
UV–visible spectra of 1.01 × 10^–6^–2.35 × 10^–6^ M CH_2_Cl_2_ solutions of **PtC**_*x*_**Pt**.

The colors of the solid monoplatinum complexes **PtC**_***x***_**Si** progressively deepened from bright yellow (*x* = 6, 8) to orange-yellow (*x* = 10) to orange-red (*x* = 12) to orange-brown (*x* = 14) to dark red (*x* = 16) to dark violet-red (*x* = 18, 20, 24, 26, 28, 32). This trend was paralleled by the diplatinum complexes **PtC**_***x***_**Pt**, and the colors exhibited by dilute solutions are illustrated in Figure s1. These observations are consistent with the UV–visible spectra summarized in Tables 3 and 4 and depicted in Figures 6 and 7.

As recognized since early studies,^13^ the absorption envelopes of many polyynes exhibit *C*≡*C* vibrational fine structure, and Figures 6 and 7 are rich in local maxima. Three trends are apparent, as is also seen with many of the other polyyne series in Figures 1 and 2. First, the longest-wavelength absorptions (λ_Emin_) red shift upon increasing the sp carbon chain length. Second, the bands become broader and fine structure weakens as the chains lengthen. Third, the λ_max_ (most intense absorption) transitions from λ_Emin_ for **PtC**_**16**_**Pt**, **PtC**_**18**_**Pt**, **PtC**_**20**_**Pt**, **PtC**_**24**_**Pt**, and **PtC**_**28**_**Pt** to the second longest wavelength band for **PtC**_**32**_**Pt** and higher homologues. The last trend, also nicely illustrated by Tr*C_***x***_Tr*,^21^ has been reproduced computationally for *trans* polyenes^50^ and is believed to have a Franck–Condon origin. Aspects of the absorption spectra that probe BLA are analyzed in the Discussion section.

### Crystallography

**Figure 8:**
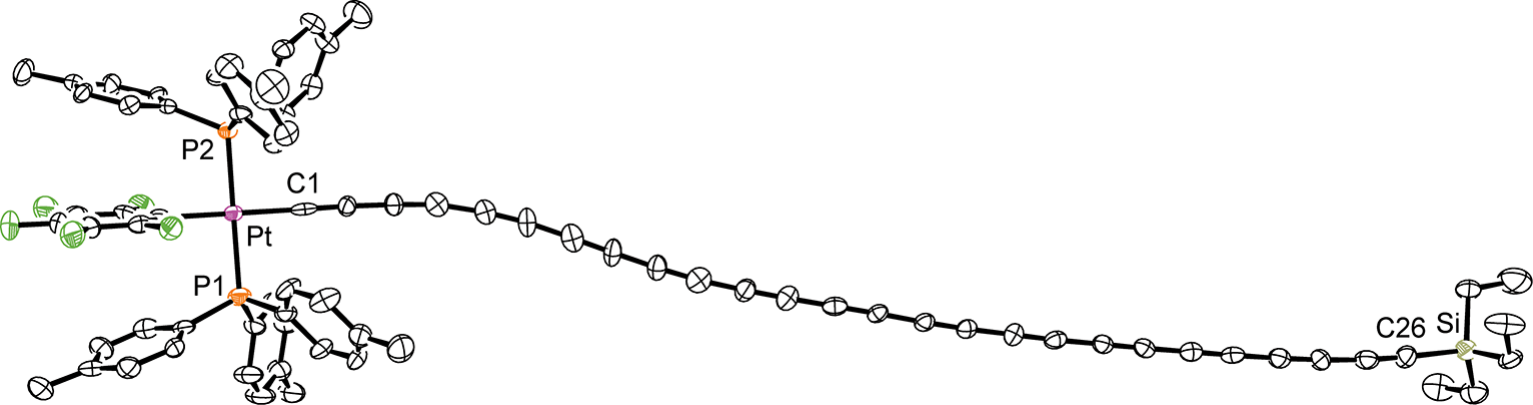
Thermal ellipsoid plot (50% probability) of the molecular structure of **PtC**_**26**_**Si**·(CH_2_Cl_2_) with hydrogen atoms and solvent molecules omitted.

**Figure 9:**
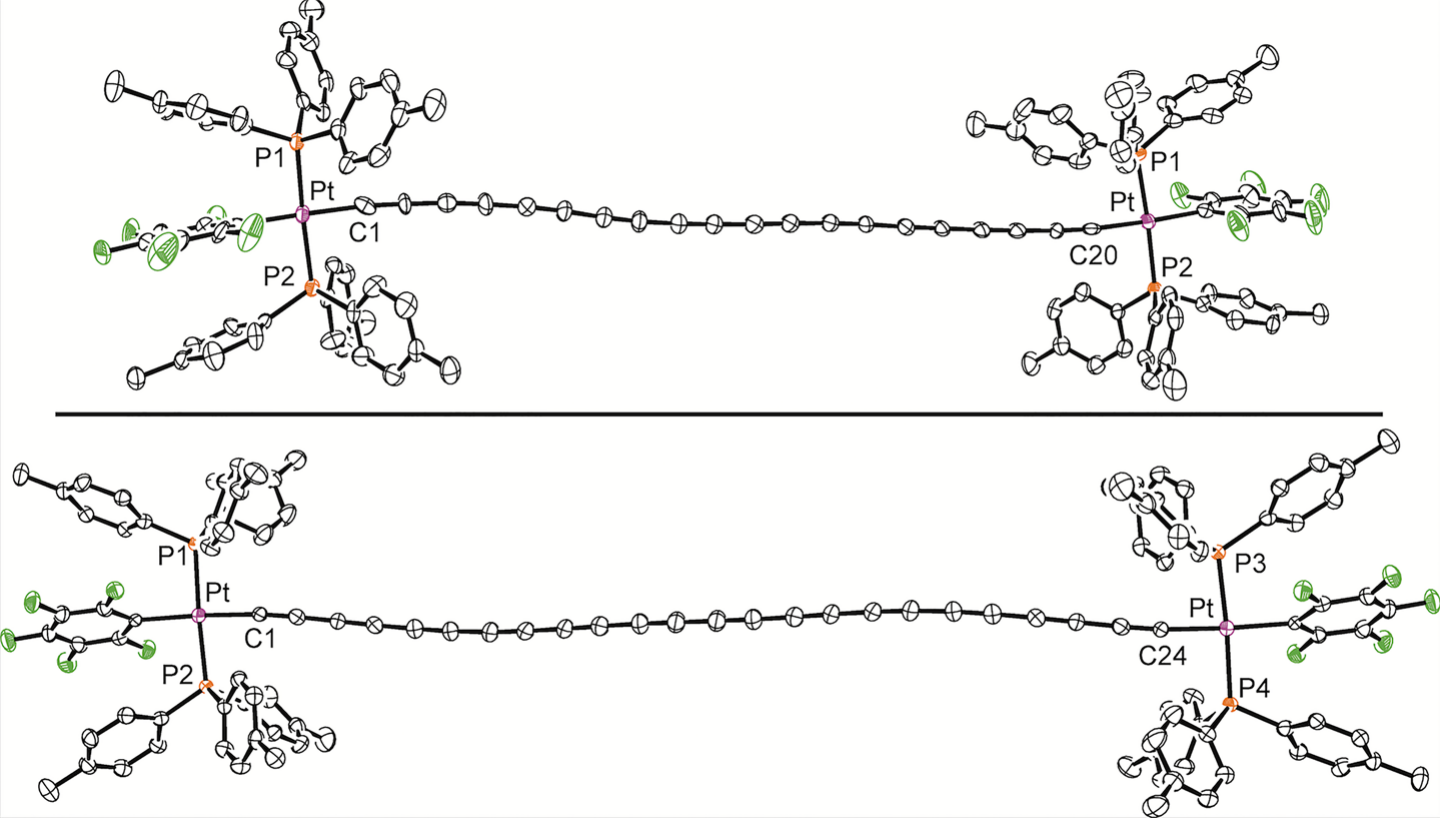
Thermal ellipsoid plots (50% probability) of the molecular structures of **PtC**_**20**_**Pt**·(CH_2_Cl_2_) (top) and **PtC**_**24**_**Pt**·(C_6_H_14_)_2_·(CH_2_Cl_2_)_1.7_ (bottom) with hydrogen atoms and solvent molecules omitted.

It was sought to crystallographically characterize as many of the preceding complexes as possible. As detailed in Table s7 and the Experimental Section, the structures of three solvates, **PtC**_**26**_**Si**·(CH_2_Cl_2_), **PtC**_**20**_**Pt**·(CH_2_Cl_2_), and **PtC**_**24**_**Pt**·(C_6_H_14_)_2_(CH_2_Cl_2_)_1.7_, could be solved. These are depicted in Figures 8 and 9. **PtC**_**26**_**Si** represents the longest structurally characterized conjugated polyyne with unlike endgroups, and **PtC**_**24**_**Pt**, the longest with like endgroups. We reported a crystal structure of the fractional solvate **PtC**_**20**_**Pt**·(CH_2_Cl_2_)_0.8_ earlier,^36^ but data for the new monosolvate are of much higher quality. The only other crystallographically characterized polyyne with at least 10 triple bonds is *t*-Bu(C≡C)_10_*t*-Bu.^16^

Key metric parameters are summarized in Tables s8 and s9, and selected data are analyzed in the Discussion section. The pentafluorophenyl ligand engages in π stacking interactions, but they are not as pronounced as for other complexes in this series, as judged from C_6_H_4_CH_3_/C_6_F_5_/C_6_H_4_CH_3_ centroid distances and angles. With **PtC**_**24**_**Pt**·(C_6_H_14_)_2_·(CH_2_Cl_2_)_1.7_, the midpoint of the C_24_ chain corresponds to the crystallographic inversion center. Chain curvature is always evident, but well within the bounds of other polyynes.^51^

**Figure 10:**
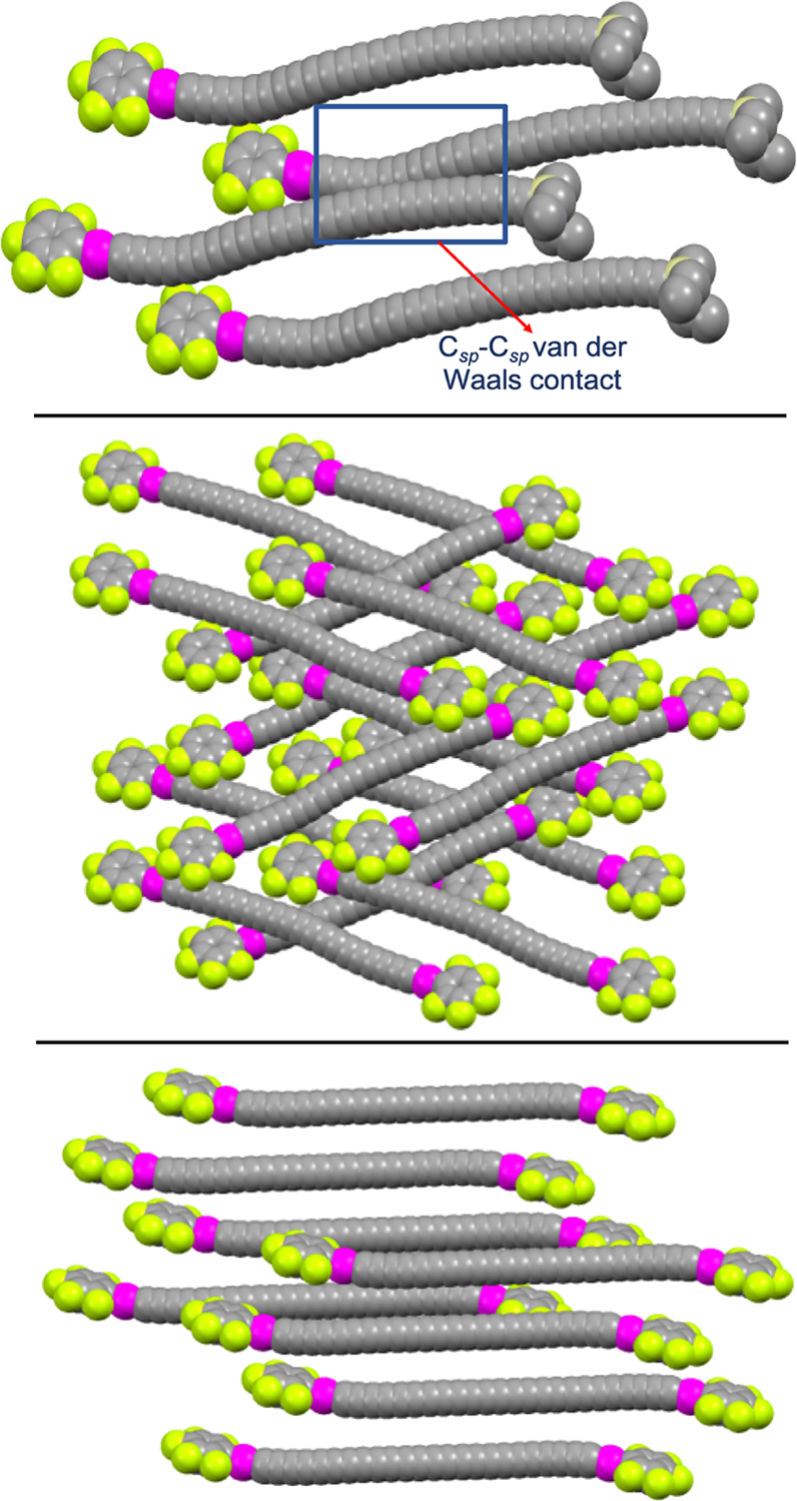
Crystal lattices of **PtC**_**26**_**Si**·(CH_2_Cl_2_) (top), **PtC**_**20**_**Pt**·(CH_2_Cl_2_) (middle), and **PtC**_**24**_**Pt**·(C_6_H_14_)_2_·(CH_2_Cl_2_)_1.7_ (bottom) with hydrogen atoms, solvent molecules, and *p*-tol_3_P ligands omitted and the remaining atoms at van der Waals radii.

The crystal packing of conjugated polyynes has been analyzed in detail,^51^ and excerpts from each lattice are depicted in Figure 10. In **PtC**_**24**_**Pt**·(C_6_H_14_)_2_·(CH_2_Cl_2_)_1.7_, the platinum–platinum vectors are parallel, with a separation of 3.94 Å. Thus, the sp chains remain out of van der Waals contact (radius of sp carbon, 1.78 Å).^52^ With **PtC**_**26**_**Si**·(CH_2_Cl_2_), the platinum–silicon vectors are parallel. Now the separation narrows to 3.57 Å such that contacts between the noticeably curved neighboring chains are apparent. The neighbors pack in head-to-head arrangements with offsets of about 11 sp carbon atoms. With **PtC**_**20**_**Pt**·(CH_2_Cl_2_), there are two nonparallel sets of parallel platinum–platinum vectors, with none of the nearest parallel chains in van der Waals contact. All of these motifs have precedent in polyyne lattices.^51^

## Discussion

### Synthetic Methodology and Stabilities

The title complexes **PtC**_***x***_**Pt** and their supporting cast **PtC**_***x***_**Si** provide the largest collections of monodisperse symmetrically and unsymmetrically substituted polyynes assembled to date. Eighteen of the 29 complexes bookended by **PtC**_**6**_**Si**/**PtC**_**32**_**Si** and **PtC**_**4**_**Pt**/**PtC**_**52**_**Pt** have been crystallographically characterized (Figure s8 and Table s10), and more are expected to follow soon, including some with much longer sp chains than **PtC**_**24**_**Pt** or **PtC**_**26**_**Si**.

There are advantages and disadvantages associated with building block **HC**_**8**_**Si** used in Scheme 3. The multiple additions provide rapid access to mixtures of monoplatinum complexes **PtC**_***x***_**Si** with long sp carbon chains. Since the components differ by eight sp carbon atoms, they are easy to chromatographically separate. But unlike **SiC**_**4**_**Si**, which can be cleanly protodesilylated to **HC**_**4**_**Si**, the more remote termini of the precursor **SiC**_**8**_**Si** resist selective functionalization (Scheme 2). With the unsymmetrical bis(trialkylsilyl) tetrayne Me_3_Si(C≡C)_4_Si(*i*-Pr)_3_, the trimethylsilyl group can be preferentially removed to give H(C≡C)_4_Si(*i*-Pr)_3_, and this has been exploited by Tykwinski for sp chain extensions en route to Tr*C_**44**_Tr* (Figure 1).^21^

A key challenge with both heterocouplings and homocouplings is the increasing lability of terminal polyynes **PtC**_***x***_**H** as the sp chains lengthen. These can be isolated in pure form only through *x* = 8,^29,37^ and some diminution in the efficacy of click trapping is apparent at *x* = 18.^38^ A possible mechanistic solution would involve the direct conversion of −(C≡C)_*n*_Si or −(C≡C)_*n*_Sn termini to transition-metal derivatives −(C≡C)_*n*_L_*y*_M(C≡C)_*n*_– capable of reductively eliminating two alkynyl ligands under mild conditions.

The heterocouplings in Scheme 3 do not seem to have approached any limit arising from the stabilities of **PtC**_***x***_**Si**. However, the situation with homocouplings in Scheme 4 is less clear. There is an apparent upper yield limit of ∼10% for **PtC**_**48**_**Pt** and **PtC**_**52**_**Pt**, which we attribute to side reactions or decomposition of the precursors **PtC**_***x***_**H**. Nonetheless, as generations of co-workers have repeated the syntheses of **PtC**_**44**_**Pt**, **PtC**_**48**_**Pt**, and **PtC**_**52**_**Pt**, we have become more sanguine about their stabilities. Pure samples stored as prescribed could be kept for weeks or longer.

As shown in Table s2, the thermal decomposition points of **PtC**_***x***_**Pt** trend downward for *x* ≥ 28 but remain above 70 °C. Decomposition may involve sp chain/sp chain cross-linking, reminiscent of solid-state topochemical polymerizations of 1,3-diynes^53^ and foreshadowed by close contacts in the lattice of **PtC**_**26**_**Si**·(CH_2_Cl_2_) (Figure 9, top). In any case, we are optimistic that higher homologues of **PtC**_**52**_**Pt** represent viable synthetic targets, although this might be facilitated by replacing the (*p*-tol)_3_P ligands by bulkier triaryl phosphines.^54^ Also, some of the above issues can be avoided by the use of “masked alkyne equivalents”. As developed by Anderson, π adducts of polyynes and Co_2_(CO)_4_L_2_ fragments are used to assemble long C_*x*_ moieties and the dicobalt moieties removed in the final step.^30^

### Properties of C_∞_ Systems

Some extrapolations of the preceding data are more relevant to polymers **PtC**_**∞**_**Pt** and **PtC**_**∞**_**Si**, and others map the dissipation of end-group effects, pointing to values expected of carbyne (**C**_**∞**_). Such limits are best estimated using the Meier equation.^55^ In the most general form (eq 1), the convergence of a property *y* of a series of unsaturated oligomers from *y*_*n*_ to a constant value at *y*_∞_ can be modeled by the exponential relationship
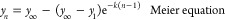
1

One overarching issue involves the electronic structure of carbyne, for which cumulated (=(C=C=)_∞_; BLA = 0) and polyyne (nonzero BLA) limits have received extensive consideration. Per the following subsections, our results add to the growing body of evidence from organic models in Figure 1 that support the polyyne formulation.^31b^ This is often couched as a Peierls distortion of the cumulated limit that installs a nonzero band gap.^10^

#### ^13^C{^1^H} NMR Data

The limits reached by the chemical shifts of the PtC≡C and C≡CSi signals with increasing *x* (Tables 1 and 2; Figure s2) clearly represent attributes of polymers **PtC**_**∞**_**Pt** and **PtC**_**∞**_**Si**. As seen in Figures 3 and 4 for **PtC**_***x***_**Pt** and **PtC**_***x***_**Si**, the chemical shifts of the terminal C≡C linkages of the other polyynes in Figures 1 and 2 also appear downfield of the remaining sp carbon signals.^31b^ This deshielding is more pronounced with electropositive transition-metal endgroups.

Other chemical shift data map the transition to carbyne. In Figure s3, the averages of all sp signals except the PtC≡C and C≡CSi linkages (*x* – 4 values total) are treated by the Meier protocol.^56^ For **PtC**_***x***_**Pt**, there is a nearly perfect monotonic trend (ppm) from 61.10 (*x* = 6) to 63.42 (*x* = 44) that extrapolates to 63.35 (*x* = ∞). The data for **PtC**_***x***_**Si** are quite similar for the corresponding values of *x* (63.02 for *x* = ∞). Thus, carbyne (**C**_**∞**_) should exhibit a signal with a mean chemical shift of ∼63.15 ppm, which by analogy to linear polyethylene^57^ should be a broad singlet. The sp signals of other polyynes in Figures 1 and 2 have been treated in somewhat different ways, but all point to comparable limits.

#### Raman Data

**Figure 11:**
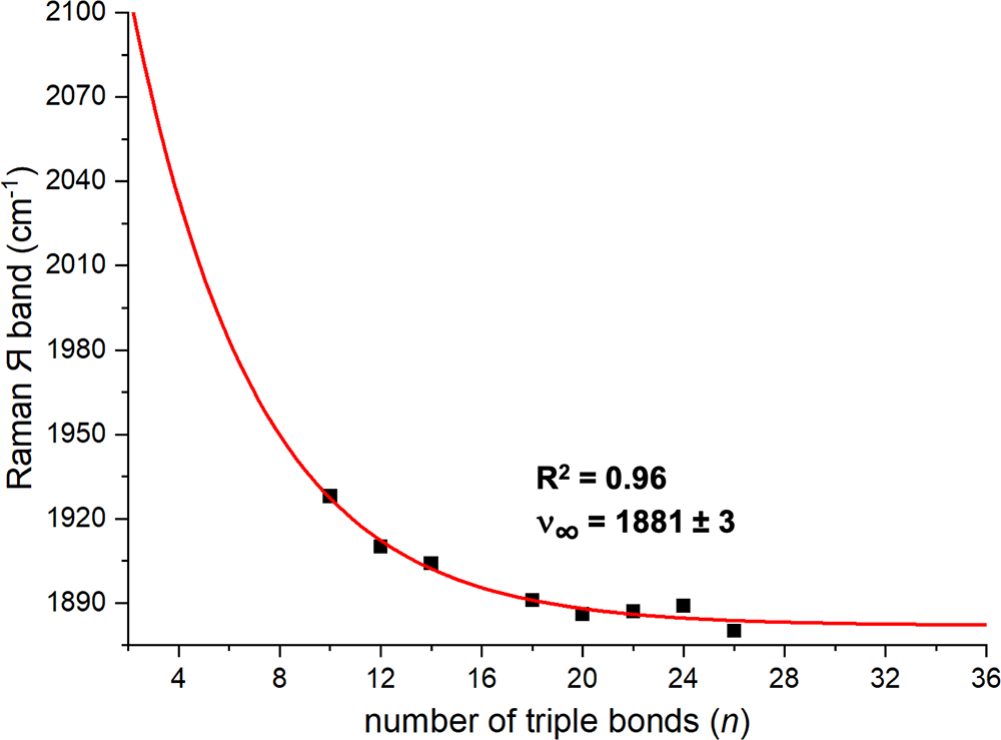
Extrapolation of the Raman Я band of **PtC**_*x*_**Pt** to *x* = ∞ by using the Meier equation.

The Raman Я band of polyynes has been shown to shift to lower energy as the BLA decreases.^49^ For **PtC**_***x***_**Pt**, they drop from 1928 cm^–1^ for *x* = 20 to 1880 cm^–1^ for *x* = 52. As depicted in Figure 11, the Meier equation indicates convergence to 1881 cm^–1^ (ν_∞_), requiring a polyyne electronic structure for **C**_**∞**_. This protocol also allows the estimation of an effective conjugation length (ECL) of 30 *C*≡*C* units, derived by limiting *y*_∞_ – *y*_ECL_ to 1 cm^–1^.^55b^ An ECL value represents the point at which the endgroups no longer affect the property of interest. With **PtC**_***x***_**Si**, some complexes exhibited multiple Raman bands of comparable intensities in this region (Figure s6). This has been seen for other unsymmetrically substituted polyynes, which as noted above are not subject to the Raman/IR mutual exclusion rule.^20^ Hence, these were not further analyzed.

The Я bands similarly converge to 1908 cm^–1^ for Tr*C_***x***_Tr*, 1865 cm^–1^ for Py*C_***x***_Py* (ECL 37), 1790 cm^–1^ for **AdC**_***x***_**Ad**, and 1835 cm^–1^ for transition-metal-substituted **ReC**_***x***_**Re** (ECL 37).^31b^ There are potential rationales for these modest differences (possible mixing of additional vibrational modes, experimental factors, etc.), but computational studies are likely required for further insight. In any case, the following conclusions emerge: (1) by the Raman criterion, **PtC**_***x***_**Pt** and all other polyyne series so analyzed exhibit marked BLA, far removed from zero, (2) the range of convergence values, 1790 to 1908 cm^–1^, can be viewed as limits on the corresponding absorption of carbyne, and (3) this range nicely agrees with experimental data for polydisperse samples of carbyne encapsulated in different types of carbon nanotubes (1850–1880 cm^–1^).^34b^

#### UV–Visible Data

As conjugated oligomers are extended, the λ_max_ and/or λ_Emin_ commonly red shift and increase in intensity. The shift is, of course, expected from basic MO theory. However, the situation with **PtC**_***x***_**Pt** is more complicated, as revealed by DFT studies of the model PH_3_ complexes *trans*,*trans*-(C_6_H_5_)(H_3_P)_2_Pt(C≡C)_*n*_Pt(PH_3_)_2_(C_6_H_5_) (**Pt′′C**_***x***_**Pt′′**, *x* = 4–26).^58^ Two π → π* transitions associated with the sp chain are predicted. That at longer wavelength (lower energy, termed band **II**) has predominant HOMO–LUMO character. However, its extinction coefficient decreases dramatically with increasing chain length. Experimentally, it is only observed with **PtC**_***x***_**Pt** with shorter chains (*x* = 4–12 but not 20), always with characteristic vibrational fine structure.^28,29^ Analogous series of bands have been found for lower homologues of many of the polyynes in Figure 1, as summarized in a recent experimental and computational study.^23^

The higher-energy band (band **I**) becomes markedly stronger with increasing chain length and is multiconfigurational with increasing HOMO–LUMO character. These afford the maxima in Figures 6 and 7, and thus the optical band gaps overestimate the true band gaps. The DFT studies furthermore show that the platinum character in the molecular orbitals of most interest (HOMO, HOMO – 1, and LUMO) decreases with increasing chain length, roughly proportional to the Pt/C_*x*_/Pt composition.^57^ The platinum contribution is significant for shorter sp chains (22% in **Pt′′C**_**6**_**Pt′′**, for which 25% of the atoms are platinum) but drops sharply for later members in the series (6% in **Pt′′C**_**20**_**Pt′′**, 9% of the atoms are platinum). Thus, the orbitals and corresponding transitions in still-higher homologues are almost exclusively carbon-chain-based.

**Figure 12:**
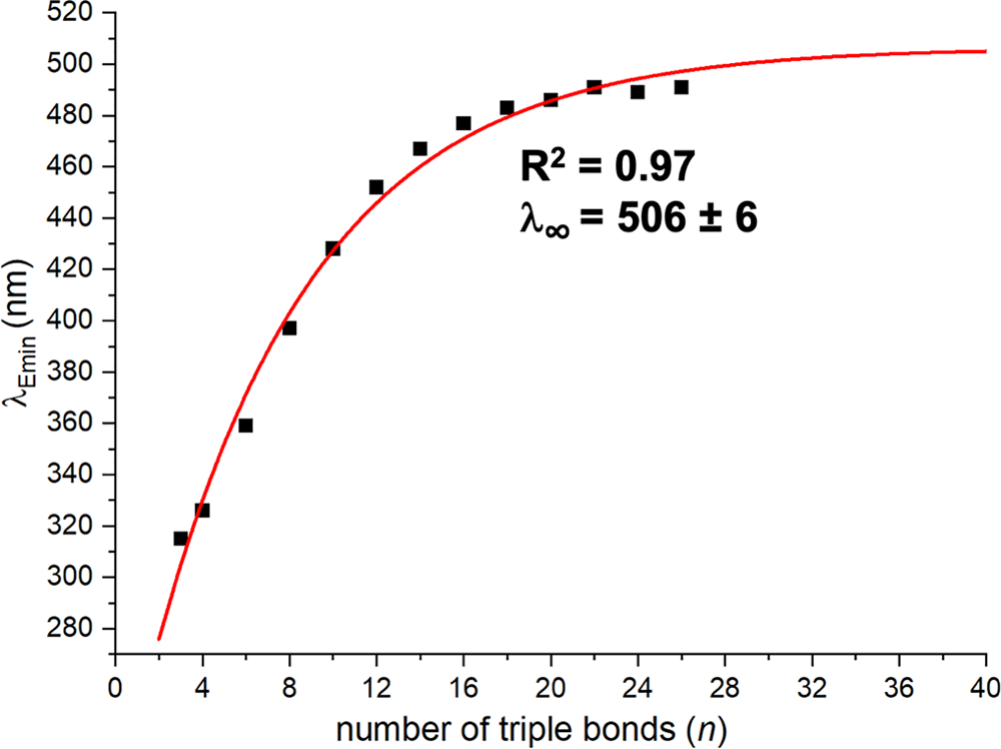
Extrapolation of the λ_Emin_ values (longest-wavelength UV–visible absorption) of **PtC_*x*_Pt** (*x* ≥ 6) to *x* = ∞ by using the Meier equation.

In Figure 12, the longest-wavelength absorptions (λ_Emin_) are plotted against the number of triple bonds *n* using the Meier equation. The excellent fit gives 506 nm for λ_Emin∞_, or an optical band gap of 2.50 eV for **C**_**∞**_. The ECL (derived by limiting λ_Emin∞_ – λ_Emin(ECL)_ to 1 nm) is ∼42. These data are quite similar to those obtained for Tr*C_***x***_Tr* (λ_Emin∞_ = 486 nm, ECL = 48),^21^ and similar values have been reported for **SiC**_***x***_**Si**, **AdC**_***x***_**Ad**, and *t*-**BuC**_***x***_*t*-**Bu** (λ_Emin∞_ = 501–503 nm).^31b^

There is some play in all of these numbers. For example, when Figure 12 is restricted to data for **PtC**_**16**_**Pt** to **PtC**_**52**_**Pt** (*n* ≥ 8), the λ_Emin∞_ drops to 493 nm and the ECL drops to 30. Depending upon how our earlier data with **Pt′C**_***x***_**Pt′** are treated, λ_Emin∞_ ranging from 558 to 636 nm can be obtained. Nonetheless, when all of these limits are taken together, they provide excellent evidence that the λ_Emin_ of **C**_**∞**_ should be ca. 500 nm. Furthermore, as elegantly presented in a recent study, the dramatic attenuation of band **II** with chain length parallels the evolution to the *D*_∞*h*_ limit of carbyne.^23b,31b^

#### Crystallographic Data

The wealth of structural data for **PtC**_***x***_**Pt** and **PtC**_***x***_**Si** might initially seem to hold promise for defining the BLA trends. However, the esd values that accompany crystallographic bond lengths and angles often constrain comparisons. With our compounds and most other polyynes, they render it virtually impossible to conclude that one *C*≡*C* or ≡*C*–*C*≡ linkage is shorter than another. As detailed in reviews,^51^ averages drawn from replicate or series of crystal structures can sometimes improve confidence. However, in many cases, computational studies (which are most often gas phase) can be regarded as more accurate.

In efforts to skirt these problems, two parameters, BLA(avg) and BLA, have been utilized. The first is the average of the ≡*C*–*C*≡ bond lengths minus that of the *C*≡*C* bond lengths. The second is the absolute value of the length of the central carbon–carbon bond minus the length of the two adjacent bonds (the central bond will be *C*≡*C* when *n* is odd and ≡*C*–*C*≡ when even). In Figure s8, these are plotted versus *n* for all of the crystal structures available for **PtC**_***x***_**Si** and **PtC**_***x***_**Pt**. While it is clear that the data are not converging toward a BLA of zero, there is no monotonic trend or Meier correlation. However, a Meier plot of our gas-phase computational data for **Pt′′C**_***x***_**Pt′′** gives a BLA(avg)_∞_ of 0.092 Å (*R*^2^ = 0.99; Figure s7).

Thus, we suggest that the bulky and polarizable platinum endgroups induce packing forces that alter the bond length patterns from those in the gas phase. However, data from crystal structures so far obtained in the series Py*C_***x***_Py* and *t*-**BuC**_***x***_*t*-**Bu** are nicely modeled by the Meier equation, giving BLA(avg)_∞_ values of 0.140 and 0.145 Å, respectively.^31b^ In contrast, there is no convergence in the case of **TIPSC**_***x***_**TIPS**. Given this result and our data in Figure s8, we feel that crystallographic correlations are likely to be uncommon.

## Conclusions

By use of the labile building block **HC**_**8**_**Si**, the sp carbon chains of **PtC**_***x***_**Si** could be extended in fewer steps than in previous efforts, thereby allowing access to the new diplatinum polyynediyl complexes **PtC**_**28**_**Pt**, **PtC**_**32**_**Pt**, **PtC**_**36**_**Pt**, **PtC**_**40**_**Pt**, **PtC**_**44**_**Pt**, **PtC**_**48**_**Pt**, and **PtC**_**52**_**Pt**. Excluding supramolecular systems,^30,34^ this series terminates with the longest polyyne isolated in pure form to date. NMR, IR, Raman, and UV–visible data as well as 18 crystal structures have been carefully analyzed as a function of chain length. Certain protocols map the attrition and ultimate loss of endgroup effects, affording experiment-based predictions for key properties of carbyne (**C**_**∞**_), which include a polyyne electronic structure with an appreciable bond length alternation and a 2.50 eV optical band gap. For the most part, these are in line with those extrapolated from analogs with organic endgroups, confirming that systems with transition-metal termini also provide valid models for the polymeric sp allotrope. This is of special interest to those who believe, as the authors, that in whatever competition may exist to isolate the longest monodisperse polyyne, transition-metal endgroups will ultimately provide the winners. In that context, **PtC**_**52**_**Pt** certainly does not represent any type of stability boundary, and the methods employed for the compounds in Figures 1 and 2 have only superficially dented the universe of sp/sp coupling methods. Accordingly, syntheses of higher homologues remain under active investigation.

## Experimental Section

### General Data

Reactions were conducted under dry inert atmospheres using conventional Schlenk techniques, but workups were carried out in air. Sources of chemicals, instrumental methods, and other protocols are summarized in the SI. (***Caution!****Polyynes normally possess highly positive heats of formation and may be regarded as energy-rich materials that are intrinsically thermodynamically unstable.*) Many explosions or rapid exothermic decompositions of polyynes have been reported. These most frequently involve species with (C≡C)_*n*_X linkages (X = H, halide). Regardless, all polyynes should be treated as potentially explosive, and appropriate safety precautions should be taken.

### **H(C≡C)_4_SiEt_3_** (**HC_8_Si**)^13^

A beaker was charged with **SiC**_**8**_**Si** (2.93 g, 8.97 mmol; see SI), pentane (4 mL), and MeOH (200 mL). Then aqueous NaOH (1.0 M; 1.0 mL, 1.0 mmol) was added. After 80 s, the solution was poured into a slurry of ice (ca. 200 g), HCl (2.0 M, 200 mL), and pentane (200 mL). The aqueous phase (together with some black particles) was separated and extracted with pentane (3 × 100 mL). The combined golden yellow pentane phases were dried (MgSO_4_, 15 min). The solution was concentrated to ca. 30 mL at 0–5 °C by rotary evaporation, kept at ≤−35 °C, and used immediately (“crude **HC**_**8**_**Si**”). TLC and click reaction assays^38^ verified a mixture of **HC**_**8**_**H**, **HC**_**8**_**Si**, and **SiC**_**8**_**Si**, and further chromatographic purification is described in the SI.

### **PtC_16_Si** and **PtC_24_Si** (from **PtC_8_Si**/**HC_8_Si**)

A three-neck flask was fitted with a gas dispersion tube, charged with **PtC**_**8**_**Si** (0.210 g, 0.178 mmol) and THF (120 mL), and cooled to −78 °C. A Schlenk flask was charged with CuCl (0.287 g, 2.90 mmol), acetone (10 mL), and TMEDA (0.801 mL, 0.621 g, 5.34 mmol) with stirring (0.5 h), after which a green solid separated from a blue supernatant. Then wet *n*-Bu_4_N^+^F^–^ (1.0 M in THF, 5 wt % water, 0.09 mL, 0.09 mmol) was added to the three-neck flask with stirring. After 20 min (TLC showed no remaining educt), Me_3_SiCl (0.18 mL, 1.4 mmol) and a −35 °C pentane solution of crude **HC**_**8**_**Si** (ca. 30 mL, from 50 equiv of **SiC**_**8**_**Si**) were added. Then oxygen was aspirated through the tube, and the blue supernatant was added with stirring.^44^ After 50 min, hexanes (100 mL) were added. The suspension was filtered through a silica gel plug (5 × 70 cm^2^, packed in 1:1 v/v acetone/hexanes), which was rinsed (1:1 v/v acetone/hexanes) until the filtrate was colorless. The solvents were removed from the filtrate by rotary evaporation at <10 °C. The red-brown residue was chromatographed on a silica gel column (4.5 × 30 cm^2^, packed in hexanes, and eluted with hexanes and then a CH_2_Cl_2_ gradient until 1:2 v/v CH_2_Cl_2_/hexanes). The solvents were removed from the product-containing fractions by rotary evaporation at <10 °C to give (in order of elution) **PtC**_**24**_**Si** as a brown-orange solid (0.020 g, 0.014 mmol, 8%) and **PtC**_**16**_**Si** as an orange solid (0.044 g, 0.034 mmol, 19%). In a second comparable experiment (SI), **PtC**_**32**_**Si** was also isolated (3%).

### **PtC_16_Si**(38)

The thermal, DSC, microanalytical, NMR, IR, UV–vis, and MS data agreed with those reported previously.

### **PtC_24_Si**

Anal. Calcd for C_78_H_57_F_5_P_2_PtSi (1373.327): C, 68.16; H, 4.18. Found: C, 68.93; H, 4.45. Thermal, IR, ^31^P{^1^H} NMR, ^1^H NMR, ^13^C{^1^H} NMR, and UV–vis data: Tables s2, s1, s1, s3, 1 and s5, and 3, respectively.

### **PtC_32_Si**

IR, ^31^P{^1^H} NMR, ^1^H NMR, ^13^C{^1^H}, and UV–vis data: Tables s1, s1, s3, 1 and s5, and 4, respectively.

### **PtC_18_Si** and **PtC_26_Si** (from **PtC_10_Si**/**HC_8_Si**)

A three-neck flask was fitted with a gas dispersion tube, charged with **PtC**_**10**_**Si** (0.203 g, 0.168 mmol) and THF (200 mL), and cooled to −78 °C. A Schlenk flask was charged with CuCl (0.240 g, 2.42 mmol), acetone (20 mL), and TMEDA (0.675 mL, 0.523 g, 4.48 mmol) with stirring (0.5 h), after which a green solid separated from the blue supernatant. Then wet *n*-Bu_4_N^+^F^–^ (1.0 M in THF, 5 wt % water, 0.08 mL, 0.08 mmol) was added to the three-neck flask with stirring. After 5 min (TLC showed no remaining educt), Me_3_SiCl (0.10 mL, 0.84 mmol) and a −35 °C pentane solution of crude **HC**_**8**_**Si** (ca. 30 mL, from 50 equiv of **SiC**_**8**_**Si**) were added. Then oxygen was aspirated through the tube, and the blue supernatant was added with stirring.^44^ After 50 min, hexanes (150 mL) were added. The suspension was filtered through a pad of silica gel (5 × 7 cm^2^, packed in 1:1 v/v acetone/hexanes), which was rinsed (1:1 v/v acetone/hexanes) until the filtrate became colorless. The solvents were removed from the filtrate by rotary evaporation at <10 °C. The red-brown residue was chromatographed on a silica gel column (4.5 × 30 cm^2^, packed in hexanes, and eluted with hexanes and then a CH_2_Cl_2_ gradient until 1:2 v/v CH_2_Cl_2_/hexanes). The solvents were removed from the product-containing fractions by rotary evaporation at <10 °C to give (in order of elution) **PtC**_**26**_**Si** as a brown-orange solid (0.015 g, 0.011 mmol, 7%) and **PtC**_**18**_**Si** as a dark-orange solid (0.035 g, 0.027 mmol, 16%). A later fraction afforded a mixture of **Pt**_**20**_**Pt**, **Pt**_**28**_**Pt**, and **Pt**_**36**_**Pt**, as assayed by MS and HPLC (see the text).

### **PtC_18_Si**(38)

The thermal, DSC, microanalytical, NMR, IR, UV–vis, and MS data agreed with those reported previously.

### **PtC_26_Si**

Anal. Calcd for C_80_H_57_F_5_P_2_PtSi (1397.327): C, 68.71; H, 4.11. Found: C, 68.32; H, 4.49. Thermal, IR, ^31^P{^1^H} NMR, ^1^H NMR, ^13^C{^1^H} and UV–vis data: Tables s2, s1, s1, s3, 1 and s5, and 4, respectively.

### **PtC_20_Si** and **PtC_28_Si** (from **PtC_12_Si**/**HC_8_Si**)

A three-neck flask was fitted with a gas dispersion tube, charged with **PtC**_**12**_**Si** (0.195 g, 0.159 mmol) and THF (60 mL), and cooled to −78 °C. A Schlenk flask was charged with CuCl (0.286 g, 3.12 mmol), acetone (8 mL), and TMEDA (0.828 mL, 0.642 g, 5.2 mmol) with stirring (0.5 h), after which a green solid separated from a blue supernatant. Then wet *n*-Bu_4_N^+^F^–^ (1.0 M in THF, 5 wt % water, 0.05 mL, 0.05 mmol) was added to the three-neck flask with stirring. After 5 min (TLC showed no remaining educt), Me_3_SiCl (0.05 mL, 0.4 mmol) and a −35 °C pentane solution of crude **HC**_**8**_**Si** (ca. 30 mL, from 50 equiv of **SiC**_**8**_**Si**) were added. Then oxygen was aspirated through the tube, and the blue supernatant was added with stirring.^44^ After 75 min, the suspension was filtered through a pad of silica gel (2.5 × 10 cm^2^, packed in 1:1 v/v acetone/hexanes), which was rinsed (1:1 v/v acetone/hexanes) until the filtrate was colorless. The solvents were removed from the filtrate by rotary evaporation. The red-brown residue was dried by oil pump vacuum and chromatographed on a silica gel column (3.5 × 30 cm^2^, packed in hexanes, and eluted with hexanes and then a CH_2_Cl_2_ gradient until 1:1 v/v CH_2_Cl_2_/ hexanes). The solvents were removed from the product-containing fractions by rotary evaporation at <10 °C to give (in order of elution) **PtC**_**28**_**Si** as a dark-violet solid (0.030 g, 0.021 mmol, 13%) and **PtC**_**20**_**Si** as a violet-red solid (0.074 g, 0.056 mmol, 35%).

### **PtC_20_Si**(38)

The thermal, DSC, microanalytical, NMR, IR, UV–vis, and MS data agreed with those reported previously.

### **PtC_28_Si**

Anal. Calcd for C_82_H_57_F_5_P_2_PtSi (1421.327): C, 69.24; H, 4.04. Found: C, 68.51; H, 4.65. Thermal, IR, ^31^P{^1^H} NMR, ^1^H NMR, ^13^C{^1^H} NMR, and UV–vis data: Tables s2, s1, s1, s3, 1 and s5, and 3, respectively.

### **PtC_28_Pt**

A three-neck flask was fitted with a gas dispersion tube, charged with **PtC**_**14**_**Si** (0.140 g, 0.112 mmol) and acetone (25 mL), and cooled to −45 °C. A Schlenk flask was charged with CuCl (0.055 g, 0.56 mmol), acetone (5 mL), and TMEDA (0.105 mL, 0.081 g, 0.67 mmol) with stirring (0.5 h), after which a green solid separated from a blue supernatant. Then oxygen was aspirated through the tube and the blue supernatant was added with stirring.^44^ After 15 min (TLC showed no reaction), wet *n*-Bu_4_N^+^F^–^ (1.0 M in THF, 5 wt % water, 0.06 mL, 0.06 mmol) was added with stirring. The dark-green suspension immediately turned reddish-brown. After 5 min, Me_3_SiCl (0.060 mL, 0.45 mmol) was added. After 75 min, the solvents were removed by rotary evaporation at ≤10 °C. The brown residue was chromatographed on a silica gel column (4.5 × 30 cm^2^, packed in hexanes, and eluted with 1:7 v/v CH_2_Cl_2_/hexanes and then a CH_2_Cl_2_ gradient until 1:3 v/v CH_2_Cl_2_/ hexanes). The solvents were removed from the product-containing fractions by rotary evaporation at <10 °C to give **PtC**_**28**_**Pt** as a brown-orange solid (0.080 g, 0.035 mmol, 62%).

Anal. Calcd for C_124_H_84_F_10_P_4_Pt_2_ (2277.529): C, 65.38; H, 3.72. Found: C, 65.71; H, 4.12. Thermal, IR, ^31^P{^1^H} NMR, ^1^H NMR, ^13^C{^1^H} NMR, and UV–vis data: Tables s2, s1, s1, s3, 2 and s6, and 4, respectively.

### **PtC_32_Pt**

A three-neck flask was fitted with a gas dispersion tube, charged with **PtC**_**16**_**Si** (0.099 g, 0.077 mmol) and acetone (60 mL), and cooled to −45 °C. A Schlenk flask was charged with CuCl (0.286 g, 3.12 mmol), acetone (8 mL), and TMEDA (0.827 mL, 0.641 g, 5.2 mmol) with stirring (0.5 h), after which a green solid separated from the blue supernatant. Oxygen was aspirated through the tube, and the blue supernatant was added with stirring.^44^ Wet *n*-Bu_4_N^+^F^–^ (1.0 M in THF, 5 wt % water, 0.05 mL, 0.05 mmol) was added with stirring. The dark-green suspension immediately turned reddish-brown. After 5 min, Me_3_SiCl (0.060 mL, 0.45 mmol) was added. After 30 min (TLC showed no remaining educt), hexanes (80 mL) were added. The mixture was filtered through a pad of silica gel (2.5 × 5 cm^2^, packed in 1:3 v/v acetone/hexanes), which was rinsed (1:3 v/v acetone/hexanes) until the filtrate became colorless. The solvents were removed from the filtrate by rotary evaporation, and the red-brown residue was chromatographed on a silica gel column (1 × 25 cm^2^, packed in hexanes, and eluted with hexanes and then a CH_2_Cl_2_ gradient until 1:3 v/v CH_2_Cl_2_/hexanes). The solvents were removed from the product-containing fractions by rotary evaporation and oil pump vacuum at <10 °C to give **PtC**_**32**_**Pt** as a red-brown solid (0.031 g, 0.013 mmol, 34%).

Anal. Calcd for C_128_H_84_F_10_P_4_Pt_2_ (2325.529): C, 66.09; H, 3.64. Found: C, 66.87; H, 3.90. Thermal, IR, ^31^P{^1^H} NMR, ^1^H NMR, ^13^C{^1^H} NMR, and UV–vis data: Tables s2, s1, s1, s3, 2 and s6, and 4, respectively.

### **PtC_36_Pt**

A three-neck flask was fitted with a gas dispersion tube, charged with **PtC**_**18**_**Si** (0.088 g, 0.068 mmol) and acetone (60 mL), and cooled to −78 °C. A Schlenk flask was charged with CuCl (0.286 g, 3.12 mmol), acetone (8 mL), and TMEDA (0.827 mL, 0.641 g, 5.2 mmol) with stirring (0.5 h), after which a green solid separated from a blue supernatant. Oxygen was aspirated through the tube, and the blue supernatant added with stirring.^44^ After 15 min (TLC showed no reaction), wet *n*-Bu_4_N^+^F^–^ (1.0 M in THF, 5 wt % water, 0.02 mL, 0.02 mmol) was added with stirring. The dark-green suspension immediately turned reddish-brown. After 5 min, Me_3_SiCl (0.05 mL, 0.4 mmol) was added. After 20 min (TLC showed no remaining educt), hexanes (240 mL) were added. The mixture was filtered through a pad of silica gel (2.5 × 5 cm^2^, packed in 1:4 v/v acetone/hexanes), which was rinsed (1:4 v/v acetone/hexanes) until the filtrate was colorless. The solvents were removed from the filtrate by rotary evaporation, and the residue was chromatographed on a silica gel column (2.5 × 30 cm^2^, packed in hexanes, and eluted with hexanes and then a CH_2_Cl_2_ gradient until 1:3 v/v CH_2_Cl_2_/hexanes). The solvents were removed from the product-containing fractions by rotary evaporation and oil pump vacuum at <10 °C to give **PtC**_**36**_**Pt** as a dark red-brown solid (0.053 g, 0.022 mmol, 65%).

Anal. Calcd for C_132_H_84_F_10_P_4_Pt_2_ (2373.529): C, 66.78; H, 3.57. Found: C, 66.70; H, 3.72. Thermal, IR, ^31^P{^1^H} NMR, ^1^H NMR, ^13^C{^1^H} NMR, and UV–vis data: Tables s2, s1, s1, s3, 2 and s6, and 4, respectively.

### **PtC_40_Pt**

Acetone (60 mL), **PtC**_**20**_**Si** (0.119 g, 0.089 mmol), CuCl (0.179 g, 1.95 mmol), acetone (5 mL), TMEDA (0.532 mL, 0.412 g, 3.25 mmol), oxygen, wet *n*-Bu_4_N^+^F^–^ (1.0 M in THF, 5 wt % water, 0.02 mL, 0.02 mmol), Me_3_SiCl (0.05 mL, 0.4 mmol), and hexanes (240 mL, TLC assay after 30 min) were combined in a procedure analogous to that for **PtC**_**36**_**Pt**. A comparable workup gave **PtC**_**40**_**Pt** as a dark-red solid (0.036 g, 0.015 mmol, 34%).

Anal. Calcd for C_136_H_84_F_10_P_4_Pt_2_ (2421.529): C, 67.44; H, 3.50. Found: C, 67.37; H, 3.74. Thermal, IR, ^31^P{^1^H} NMR, ^1^H NMR, ^13^C{^1^H} NMR, and UV–vis data: Tables s2, s1, s1, s3, 2 and s6, and 4, respectively.

### **PtC_44_Pt**

Acetone (40 mL), **PtC**_**22**_**Si** (0.051 g, 0.038 mmol), CuCl (0.082 g, 0.83 mmol), acetone (5 mL), TMEDA (0.212 mL, 0.164 g, 1.4 mmol), oxygen, wet *n*-Bu_4_N^+^F^–^ (1.0 M in THF, 5 wt % water, 0.016 mL, 0.016 mmol), Me_3_SiCl (0.04 mL, 0.33 mmol), and hexanes (100 mL, TLC assay after 3 h) were combined in a procedure analogous to that for **PtC**_**36**_**Pt**. A comparable workup gave **PtC**_**44**_**Pt** as a dark-red solid (0.011 g, 0.0045 mmol, 22%).

Anal. Calcd for C_140_H_84_F_10_P_4_Pt_2_ (2469.529): C, 68.03; H, 3.40. Found: C, 67.79; H, 3.74. Thermal, IR, ^31^P{^1^H} NMR, ^1^H NMR, ^13^C{^1^H} NMR, and UV–vis data: Tables s2, s1, s1, s3, 2 and s6, and 4, respectively.

### **PtC_48_Pt**

Acetone (40 mL), **PtC**_**24**_**Si** (0.037 g, 0.027 mmol), CuCl (0.025 g, 2.5 mmol), acetone (5 mL), TMEDA (0.056 mL, 0.043 g, 0.37 mmol), oxygen, wet *n*-Bu_4_N^+^F^–^ (1.0 M in THF, 5 wt % water, 0.026 mL, 0.026 mmol), Me_3_SiCl (0.036 mL, 0.29 mmol), and hexanes (100 mL, TLC assay after 3 h) were combined in a procedure analogous to that for **PtC**_**36**_**Pt**. A comparable workup gave **PtC**_**48**_**Pt** as a dark-red solid (0.0061 g, 0.0024 mmol, 9%).

Anal. Calcd for C_136_H_84_F_10_P_4_Pt_2_ (2517.529): C, 68.65; H, 3.33. Found: C, 68.02; H, 4.36.^59^ Thermal, IR, ^31^P{^1^H} NMR, ^1^H NMR, ^13^C{^1^H} NMR, and UV–vis data: Tables s2, s1, s1, s3, s6, and 4, respectively.

### **PtC_52_Pt**

Acetone (40 mL), **PtC**_**26**_**Si** (0.043 g, 0.031 mmol), CuCl (0.027 g, 2.75 mmol), acetone (5 mL), TMEDA (0.062 mL, 0.048 g, 0.41 mmol), oxygen, wet *n*-Bu_4_N^+^F^–^ (1.0 M in THF, 5 wt % water, 0.029 mL, 0.029 mmol), Me_3_SiCl (0.04 mL, 0.33 mmol), and hexanes (100 mL, TLC assay after 3 h) were combined in a procedure analogous to that for **PtC**_**36**_**Pt**. A comparable workup gave **PtC**_**52**_**Pt** as a dark-red solid (0.0068 g, 0.0027 mmol, 9%).

Anal. Calcd for C_136_H_84_F_10_P_4_Pt_2_ (2565.529): C, 69.23; H, 3.27. Found: C, 68.04; H, 4.32.^59^ Thermal, IR, ^31^P{^1^H}, ^1^H NMR, ^13^C{^1^H} NMR, and UV–vis data: Tables s2, s1, s1, s3, s6, and 4, respectively.

## Supporting Information Available

Additional syntheses and crystallography; thermal stability; and NMR (^1^H/^31^P{^1^H}/^19^F{^1^H}), IR, Raman, UV–visible, and MS data (PDF)

## Accession Codes

CCDC 2271515, 2271513, and 2271514 contain the supplementary crystallographic data for this paper. These data can be obtained free of charge via www.ccdc.cam.ac.uk/data_request/cif, or by emailing data_request@ccdc.cam.ac.uk, or by contacting The Cambridge Crystallographic Data Centre, 12 Union Road, Cambridge CB2 1EZ, UK; fax: + 44 1223 336033.
