# Supplementary material for: Monodisperse Molecular Models for the sp Carbon Allotrope Carbyne; Syntheses, Structures, and Properties of Diplatinum Polyynediyl Complexes with PtC20Pt to PtC52Pt Linkages
Source: ACS Cent Sci. 2023 Nov 16;9(12):2225–40. doi: 10.1021/acscentsci.3c01090 (PMC10755852; doi:10.1021/acscentsci.3c01090)
Supplement: Supplementary file 1 — oc3c01090_si_001.pdf [file oc3c01090_si_001.pdf]

SUPPORTING INFORMATION FOR

Monodisperse Molecular Models for the sp Carbon Allotrope Carbyne; Syntheses, Structures, and Properties of Diplatinum Polyynediyl Complexes with $PtC_{20}Pt$ to $PtC_{52}Pt$ Linkages

Aayushi Arora,[†] Sourajit Dey Baksi,[†] Nancy Weisbach, Hashem Amini, Nattamai Bhuvanesh, and John A. Gladysz*

Department of Chemistry, Texas A&M University, PO Box 30012, College Station, Texas 77842-3012, USA

email: gladysz@chem.tamu.edu

Experimental Section (Part 2)

General Data. Reactions were conducted under dry inert atmospheres using conventional Schlenk techniques, but workups were carried out in air. Chemicals were treated as follows: acetone, distilled from CaH_2; THF, CH_2Cl_2, and diethyl ether, passed through a Glass Contour solvent purification system; ethyl acetate, hexanes, pentane, EtOH, and MeOH (5 × ACS grade), Me_3SiCl (98+%/Alfa Aesar or 98%/Acros), CuCl (99.999%/Alfa Aesar or 99.99%/Aldrich), TMEDA (99% /Acros), n-Bu_4N^+ F^- (1.0 M in THF, 5 wt% water/Acros), $CDCl_3$ (Cambridge Isotope Laboratories) and silica gel (Acros, Fluoroflash or 60M Macherey-Nagel), used as received.

NMR spectra were obtained on standard 500 MHz spectrometers and referenced as follows (δ/ppm): ^1H, residual $CHCl_3$ (7.24); $^{13}C\{^1H\}$, internal $CDCl_3$ (77.0); ^{19}F, external C_6F_6 (–164.9); $^{31}P\{^1H\}$, external H_3PO_4 (0.00). IR spectra were recorded on a Shimadzu IRAffinity-1 spectrometer with a Pike MIRacle ATR system (diamond crystal). UV-visible spectra were recorded on a Shimadzu UV-1800 spectrometer. Mass spectra were recorded using Bruker microFlex (MALDI-TOF-MS) or Thermo Scientific Q Exactive Focus (ESI and APCI) instruments. Thermal behavior was assayed with OptiMelt MPA100, Mettler-Toledo DSC-821 or TA Instruments DSC-2500 systems.[s1] Raman spectra were recorded using a WITec Alpha 300RA confocal microscope equipped with a 488 nm diode laser, a UHTS 300 VIS spectrometer, and a cooled Andor EMCCD detector at –60 °C. The laser intensity was ~250 uW at the 100× objective (Nikon E Plan, 0.9 NA) in air. A 600 g mm^{-1} grating (resolution < 3 cm^{-1}) was used and all spectra (recorded on ~1 mM CH_2Cl_2 solutions) are the composition of 20 consecutive spectra with 3 s integration times. Thin-layer chromatography (TLC) was carried out on EMD Silica Gel 60 F_{254} aluminum plates and visualized using 254 or 365 nm lamps. Microanalyses were conducted by Atlantic Microlab.

New Syntheses of Previously Reported Complexes

The syntheses of **$PtC_{20}Pt$** and **$PtC_{24}Pt$** reported earlier[s2] used precursors with $PtC_{10}Si(i$-$Pr)_3$ and $PtC_{12}Si(i$-$Pr)_3$ linkages. Analogous syntheses from the $SiEt_3$ homologs **$PtC_{10}Si$** and **$PtC_{12}Si$** (Scheme 4) follow. All spectroscopic data agreed with those described previously.

$PtC_{20}Pt$. A three-neck flask was charged with **$PtC_{10}Si$** (0.073 g, 0.061 mmol)[s3] and acet-

one (20 mL), fitted with a gas dispersion tube and a condenser, and cooled to 0 °C. A Schlenk flask was charged with CuCl (0.0302 g, 0.304 mmol), acetone (5 mL), and TMEDA (0.092 mL, 0.61 mmol) with stirring (0.5 h), after which a green solid separated from a blue supernatant. Then oxygen was bubbled through the tube and the blue supernatant added with stirring. After 0.5 h (TLC showed no reaction), wet n-$Bu_4N^+ F^-$ (1.0 M in THF, 5 wt% water; 0.024 mL, 0.024 mmol) was added with stirring. After 0.5 h, Me_3SiCl (0.093 mL, 0.73 mmol) was added. After 2.5 h, the solvent was removed by rotary evaporation (ice bath). The residue was chromatographed at 0 °C on a jacketed silica gel column (20 × 2 cm, eluted with 20:80 v/v ethyl acetate/hexanes). The solvent was removed from the product-containing fractions by rotary evaporation (ice bath) and oil pump vacuum to give **$PtC_{20}Pt$** as a yellow-brown solid (0.051 g, 0.023 mmol, 76%), which slightly darkened at 140 °C and turned black without melting at 170 °C (sealed capillary).

$PtC_{24}Pt$. A three-neck flask was charged with **$PtC_{12}Si$** (0.059 g, 0.048 mmol)[s3] and acetone (20 mL), fitted with a gas dispersion tube and a condenser, and cooled to 0 °C. A Schlenk flask was charged with CuCl (0.019 g, 0.19 mmol), acetone (5 mL), and TMEDA (0.72 mL, 0.48 mmol) with stirring (0.5 h), after which a green solid separated from a blue supernatant. Then oxygen was bubbled through the tube and the blue supernatant added with stirring. After 0.5 h (TLC showed no reaction), wet n-$Bu_4N^+ F^-$ (1.0 M in THF, 5 wt% water; 0.009 mL, 0.009 mmol) was added with stirring. After 0.5 h, Me_3SiCl (0.010 mL, 0.57 mmol) was added. After 2 h, the solvent was removed by rotary evaporation (ice bath). The residue was chromatographed at 0 °C on a jacketed silica gel column (20 × 2 cm, eluted with 20:80 v/v ethyl acetate/hexanes). The solvent was removed from the product-containing fractions by rotary evaporation (ice bath) and oil pump vacuum to give **$PtC_{24}Pt$** as a red-brown solid (0.052 g, 0.023 mmol, 96%), which slightly darkened at 120 °C and turned black without melting at 160 °C (sealed capillary).

SiC_8Si.[s4] A three-neck flask was fitted with a gas dispersion tube, charged with **HC_4Si** (9.501 g, 57.82 mmol)[s4,s5] and acetone (150 mL), and cooled to 0 °C. A Schlenk flask was charged with CuCl (0.750 g, 7.58 mmol), acetone (20 ml), and TMEDA (0.375 mL, 0.291 g, 2.05 mmol) with stirring (0.5 h), after which a green solid separated from a blue supernatant. Then oxygen was

aspirated through the tube and the blue supernatant added with stirring. After 15 min, the cooling bath was removed. After 90 min, the mixture was poured into aqueous HCl (2.0 M, 200 mL), which was extracted with diethyl ether (3 × 150 mL). The combined extracts were dried ($MgSO_4$). The solvents were removed by rotary evaporation to give a brownish oil. The residue was dissolved in a minimum of HCl/EtOH (1:100 v/v) and kept at −35 °C. Yellow needles formed, which were collected by filtration, washed with cold EtOH, and air-dried to give **SiC_8Si** as light yellow needles (7.854 g, 24.05 mmol, 83%).

NMR (δ/ppm, $CDCl_3$): ^{1}H (500 MHz) 0.99 (t, $^3J_{HH}$ = 7.9 Hz, 18H, C$\underline{H}_3$), 0.64 (q, $^3J_{HH}$ = 7.9 Hz, 12H, SiC$\underline{H}_2$); ^{13}C{^{1}H} (126 MHz) 89.0 (s, $\underline{C}$≡CSi),[s6] 86.5 (s, C≡$\underline{C}$Si),[s6] 62.3, 61.8 (2 s, SiC≡C$\underline{C}$≡$\underline{C}$), 7.5 (s, $\underline{C}H_3$), 4.2 (s, $^1J_{CSi}$ = 56.7 Hz, Si$\underline{C}H_2$).[s7]

Syntheses of Additional New Complexes

The following extends the preparation of **$PtC_{20}Si$** from **$PtC_{16}Si$** reported earlier[s3] to the next higher homolog (see general protocol, Scheme 1).[s8]

$PtC_{22}Si$. A three-neck flask was fitted with a gas dispersion tube, charged with **$PtC_{18}Si$** (0.104 g, 0.081 mmol)[s3] and THF (70 mL), and cooled to −78 °C. A Schlenk flask was charged with CuCl (0.072 g, 8.8 mmol), acetone (10 mL), and TMEDA (0.12 mL, 0.091 g, 0.78 mmol) with stirring, after which a green solid separated from a blue supernatant. Then wet n-Bu_4N^+ F^- (1.0 M in THF, 5 wt% water, 0.070 mL, 0.070 mmol) was added to the three-neck flask with stirring. After 5 min (silica gel TLC, 1:9 v/v CH_2Cl_2/hexanes, showed no remaining educt), Me_3SiCl (0.061 mL, 0.78 mmol) was added. After 5 min, **HC_4Si** (0.501 g, 3.05 mmol; precooled to –35 °C)[s3] was added. Then oxygen was aspirated through the tube, and the blue supernatant added with stirring.[s9] After 5 min, the cooling bath was removed. After 50 min, hexanes (100 mL) were added. The dark brown suspension was filtered through a pad of silica gel (5 × 7 cm, packed in hexanes), which was rinsed (1:1 v/v acetone/hexanes) until the filtrate became colorless. The solvents were removed from the filtrate by rotary evaporation at <10 °C. The red-brown residue was chromatographed on a silica gel column (4.5 × 30 cm, packed in hexanes, eluted with hexanes and then a CH_2Cl_2 gradient until 3:1 v/v CH_2Cl_2/hexanes). The solvents were removed from the

product-containing fractions by rotary evaporation at <10 °C to give a dark orange oil. Cold MeOH was added, and the precipitate was collected by filtration and dried by oil pump vacuum to give **PtC$_{22}$Si** as a dark orange-red powder (0.032 g, 0.024 mmol, 22%), which slightly darkened at 71 °C (sealed capillary).[s8] Anal. Calcd for $C_{76}H_{57}F_5P_2PtSi$ (1349.327): C, 67.01; H, 4.33. Found: C, 67.40; H, 4.62.

Alternative or Replicate Syntheses of New Complexes

To help ensure reproducibility, all new complexes in this study were independently synthesized by at least two coworkers. Some representative replicate syntheses, which bookend the yield ranges in Scheme 4, follow.

PtC$_{16}$Si, **PtC$_{24}$Si**, and **PtC$_{32}$Si** (from **PtC$_8$Si/HC$_8$Si).** A three-neck flask was fitted with a gas dispersion tube, charged with **PtC$_8$Si** (0.202 g, 0.171 mmol)[s10] and acetone (60 mL), and cooled to −78 °C. A Schlenk flask was charged with CuCl (0.286 g, 3.12 mmol), acetone (8 mL), and TMEDA (0.80 mL, 0.64 g, 5.2 mmol) with stirring (0.5 h), after which a green solid separated from a blue supernatant. Then wet n-Bu$_4$N$^+$ F$^-$ (1.0 M in THF, 5 wt% water, 0.08 mL, 0.08 mmol) was added to the three-neck flask with stirring. After 10 min (TLC, silica gel, 1:9 v/v ethyl acetate/hexanes, showed no remaining educt), Me$_3$SiCl (0.10 mL, 0.78 mmol) and a −78 °C hexane solution of **HC$_8$Si** (from 50 equiv of **SiC$_8$Si**)[s11] were added. Then oxygen was aspirated through the tube and the blue supernatant added with stirring.[s9] After 45 min (TLC showed no remaining educt), hexanes (150 mL) were added. The suspension was filtered through a pad of silica gel (3 × 10 cm, packed in 1:1 v/v acetone/hexanes), which was rinsed (1:1 v/v acetone/hexanes) until the filtrate became colorless. The solvents were removed from the filtrate by rotary evaporation. The red-brown residue was dried by oil pump vacuum and chromatographed on a silica gel column (3.5 × 30 cm, packed in hexanes, eluted with hexanes and then a CH_2Cl_2 gradient until 1:2 v/v CH_2Cl_2/hexanes). The solvents were removed from the product containing fractions by rotary evaporation and oil pump vacuum to give (in order of elution) **PtC$_{32}$Si** as a dark red solid (0.007 g, 0.005 mmol, 3%), **PtC$_{24}$Si** as a dark violet solid (0.031 g, 0.022 mmol, 13%), and **PtC$_{16}$Si** as a dark red-brown solid (0.095 g, 0.074 mmol, 43%).

$PtC_{18}Si$ and $PtC_{26}Si$ (from **$PtC_{10}Si$/HC_8Si**)**.** A three-neck flask was fitted with a gas dispersion tube, charged with **$PtC_{10}Si$** (0.202 g, 0.167 mmol)[s3] and THF (200 mL), and cooled to −78 °C. A Schlenk flask was charged with CuCl (0.286 g, 3.12 mmol), acetone (8 mL), and TMEDA (0.8 mL, 0.64 g, 5.2 mmol) with stirring (0.5 h), after which a green solid separated from a blue supernatant. Then wet n-Bu_4N^+ F^- (1.0 M in THF, 5 wt% water, 0.08 mL, 0.08 mmol) was added to the three-neck flask with stirring. After 5 min (TLC, silica gel, 1:9 v/v ethyl acetate/hexanes, showed no remaining educt), Me_3SiCl (0.08 mL, 0.63 mmol) and a −78 °C hexane solution of **HC_8Si** (from 50 equiv **SiC_8Si**)[s11] were added. Then oxygen was aspirated through the tube and the blue supernatant added with stirring.[s9] After 20 min (TLC showed no remaining educt), hexanes (150 mL) were added. The suspension was filtered through a pad of silica gel (2.5 × 10 cm, packed in 1:1 v/v acetone/hexanes), which was rinsed (1:1 v/v acetone/hexanes) until the filtrate became colorless. The solvents were removed from the filtrate by rotary evaporation. The red-brown residue was dried by oil pump vacuum and chromatographed on a silica gel column (3.5 × 30 cm, packed in hexanes, eluted with hexanes and then a CH_2Cl_2 gradient until 1:2 v/v CH_2Cl_2/hexanes). The solvents were removed from the product-containing fractions by rotary evaporation and oil pump vacuum to give **$PtC_{26}Si$** as a dark violet solid (0.030 g, 0.021 mmol, 13%) and **$PtC_{18}Si$** as a violet solid (0.068 g, 0.052 mmol, 31%).

$PtC_{28}Pt$. A three-neck flask was fitted with a gas dispersion tube, charged with **$PtC_{14}Si$** (0.163 g, 0.130 mmol) and acetone (80 mL), and cooled to −78 °C. A Schlenk flask was charged with CuCl (0.286 g, 3.12 mmol), acetone (8 mL), and TMEDA (0.80 mL, 0.64 g, 5.2 mmol) with stirring (0.5 h), after which a green solid separated from a blue supernatant. Oxygen was aspirated through the tube and the blue supernatant added with stirring. After 15 min (TLC showed no reaction), wet n-Bu_4N^+ F^- (1.0 M in THF, 5 wt% water, 0.05 mL, 0.05 mmol) was added with stirring and the dark green suspension immediately turned reddish-brown. After 20 min (TLC showed no remaining educt), hexanes (240 mL) were added. The mixture was filtered through a pad of silica gel (2.5 × 7 cm, packed in 1:4 v/v acetone/hexanes), which was rinsed (1:4 v/v acetone/hexanes) until the filtrate was colorless. The solvents were removed from the filtrate by rotary evaporation

and the brown residue chromatographed on a silica gel column (2.5 × 30 cm, packed in hexanes, eluted with hexanes and then a CH_2Cl_2 gradient until 1:1 v/v CH_2Cl_2/hexanes). The solvents were removed from the product containing fractions by rotary evaporation and oil pump vacuum to give **PtC$_{28}$Pt** as a dark violet solid (0.130 g, 0.0632 mmol, 88%).

Additional Characterization of Complexes

■ <u>Mass spectrometry</u>[s12]

PtC$_{16}$Si[s3] (MALDI$^+$, matrix: DCTB + 0.1% TFA in THF): 1277 ([**PtC$_{16}$Si**]$^+$, 9%), 1109 ([**PtC$_{16}$Si** – C_6F_5 – 1]$^+$, 4%), 970 ([(tol$_3$P)$_2$PtC$_6$F$_5$]$^+$, 15%), 803 ([(tol$_3$P)$_2$Pt]$^+$, 100%).

PtC$_{18}$Si[s3] (MALDI$^+$, matrix: DCTB + 0.1% TFA in THF): 1301 ([**PtC$_{18}$Si**]$^+$, 12%), 970 ([(tol$_3$P)$_2$PtC$_6$F$_5$]$^+$, 86%), 803 ([(tol$_3$P)$_2$Pt]$^+$, 100%).

PtC$_{20}$Si[s3] (MALDI$^+$, matrix: DCTB + 0.1% TFA in THF): 1325 ([**PtC$_{20}$Si**]$^+$, 2%), 970 ([(tol$_3$P)$_2$PtC$_6$F$_5$]$^+$, 48%), 802 ([(tol$_3$P)$_2$Pt – 1]$^+$, 100%).

PtC$_{22}$Si (APCI$^-$): 1349 ([**PtC$_{22}$Si**]$^-$), 4%), 863 (unknown, 100%).

PtC$_{24}$Si (MALDI$^+$, matrix: DCTB + 0.1% TFA in THF): 1373 ([**PtC$_{24}$Si**]$^+$), 100%).

PtC$_{26}$Si (MALDI$^+$, matrix: DCTB + 0.1% TFA in THF): 1397 ([**PtC$_{26}$Si**]$^+$, 100%), 789 ([(tol$_3$P)$_2$Pt – CH$_3$ + 1]$^-$, 63%), 594 ([(tol$_3$P)$_2$ – CH$_3$ + 1]$^-$, 100%).

PtC$_{28}$Si (MALDI$^-$, matrix: DCTB + 0.1% TFA in THF): 1421 ([**PtC$_{28}$Si**]$^-$, 87%), 813 (unknown, 100%).

PtC$_{32}$Si (MALDI$^-$, matrix: DCTB + 0.1% TFA in THF): 1470 ([**PtC$_{32}$Si**]$^-$, 95%), 666 (unknown, 100%).

PtC$_{28}$Pt (MALDI$^+$, matrix: DCTB + 0.1% TFA in THF): 2277 ([**PtC$_{28}$Pt**]$^+$, 2%), 1611 ([(tol$_3$P)$_3$PtC$_{28}$C$_6$F$_5$]$^+$, 100%), 1587 ([(C$_6$F$_5$)(tol$_3$P)$_3$PtC$_{26}$]$^+$, 17%), 1563 ([(C$_6$F$_5$)(tol$_3$P)$_3$Pt-C$_{24}$]$^+$, 14%); (MALDI$^-$, matrix: DCTB + 0.1% TFA, in THF): 2276 ([**PtC$_{28}$Pt**]$^-$, 100%).

PtC$_{32}$Pt (MALDI$^-$, matrix: DCTB + 0.1% TFA in THF): 2325 ([**PtC$_{32}$Pt**]$^-$, 79%), 559 (unknown, 100%).

PtC$_{40}$Pt (MALDI$^+$, matrix: DCTB + 0.1% TFA in CH$_2$Cl$_2$): 2421 ([**PtC$_{40}$Pt**]$^+$, 0.5%), 970 ([(C$_6$F$_5$)(tol$_3$P)$_2$Pt]$^+$, 100%), 803 ([(*p*-tol$_3$P)$_2$Pt]$^+$, 92%).

PtC$_{44}$Pt (MALDI$^+$, matrix: DCTB + 0.1% TFA in CH$_2$Cl$_2$): 2469 ([**PtC$_{44}$Pt**]$^+$, 0.5%), 970 ([(C$_6$F$_5$)(tol$_3$P)$_2$Pt]$^+$, 100%).

PtC$_{48}$Pt (MALDI$^+$, matrix: DCTB + 0.1% TFA in CH$_2$Cl$_2$): 2862 (unknown, 100%), 2516 ([**PtC$_{48}$Pt** – 1]$^+$, 33%), 970 ([(C$_6$F$_5$)(tol$_3$P)$_2$Pt]$^+$, 95%).

$PtC_{52}Pt$ (MALDI$^-$, matrix: DCTB + 0.1% TFA in CH_2Cl_2): 2564 ([**$PtC_{52}Pt$**]$^-$, 0.6%), 1361 (unknown, 2%), 970 ($[(C_6F_5)(tol_3P)_2Pt]^+$, 100%).

■ $^{19}F\{^{1}H\}$ NMR (δ/ppm, $CDCl_3$, 470 MHz)

$PtC_{22}Si$: −115.85 to −116.55 (m, $^3J_{FPt}$ = 294 Hz, *o* to Pt),[s7] −163.29 to −163.45 (m, 2F, *m* to Pt), −163.57 (t, $^3J_{FF}$ = 18.9 Hz, 1F, *p* to Pt).

$PtC_{24}Si$: −115.88 to −116.56 (m, $^3J_{FPt}$ = 296 Hz, *o* to Pt),[s7] −163.29 to −163.42 (m, 2F, *m* to Pt), −163.55 (t, $^3J_{FF}$ = 18.8 Hz, 1F, *p* to Pt).

$PtC_{26}Si$: −115.86 to −116.54 (m, $^3J_{FPt}$ = 291 Hz, *o* to Pt),[s7] −163.27 to −163.40 (m, 2F, *m* to Pt), −163.52 (t, $^3J_{FF}$ = 21.1 Hz, 1F, *p* to Pt).

$PtC_{28}Si$: −116.10 to −116.18 (m, $^3J_{FPt}$ = 291 Hz, *o* to Pt),[s7] −163.22 to −163.35 (m, 2F, *m* to Pt), −163.48 (t, $^3J_{FF}$ = 18.9 Hz, 1F, *p* to Pt).

$PtC_{32}Si$: −116.16 to −116.24 (m, *o* to Pt), −163.30 to −163.41 (m, 2F, *m* to Pt), −163.53 (t, $^3J_{FF}$ = 18.7 Hz, 1F, *p* to Pt).

$PtC_{28}Pt$ (two independent samples): (1) −117.77 to −117.85 (m, $^3J_{FPt}$ = 290 Hz, *o* to Pt),[s7] −164.94 to −165.04 (m, 2F, *m* to Pt), −165.19 (t, $^3J_{FF}$ = 18.8 Hz, 1F, *p* to Pt); (2) −115.9 to −116.6 (m, $^3J_{FPt}$ = 291 Hz, *o* to Pt),[s7] −163.3 to −163.5 (m, 2F, *m* to Pt), −163.6 (t, $^3J_{FF}$ = 21.1 Hz, 1F, *p* to Pt).

$PtC_{32}Pt$: −116.14 to −116.22 (m, $^3J_{FPt}$ = 291 Hz, *o* to Pt),[s7] −163.28 to −163.39 (m, 2F, *m* to Pt), −163.52 (t, $^3J_{FF}$ = 19.2 Hz, 1F, *p* to Pt).

$PtC_{36}Pt$: −116.08 to −116.17 (m, $^3J_{FPt}$ = 292 Hz, *o* to Pt),[s7] −163.23 to −163.32 (m, 2F, *m* to Pt), −163.47 (t, $^3J_{FF}$ = 19.1 Hz, 1F, *p* to Pt).

$PtC_{40}Pt$: −116.06 to −116.14 (m, $^3J_{FPt}$ = 291 Hz, *o* to Pt),[s7] −163.22 to −163.33 (m, 2F, *m* to Pt), −163.47 (t, $^3J_{FF}$ = 19.1 Hz, 1F, *p* to Pt).

$PtC_{44}Pt$: −116.06 to −116.14 (m, $^3J_{FPt}$ = 291 Hz, *o* to Pt),[s7] −163.22 to −163.33 (m, 2F, *m* to Pt), −163.47 (t, $^3J_{FF}$ = 19.1 Hz, 1F, *p* to Pt).

$PtC_{48}Pt$: −116.01 to −116.09 (m, $^3J_{FPt}$ = 291 Hz, *o* to Pt),[s7] −163.20 to −163.31 (m, 2F, *m* to Pt), −163.42 (t, $^3J_{FF}$ = 19.1 Hz, 1F, *p* to Pt).

$PtC_{52}Pt$: −116.06 to −116.14 (m, $^3J_{FPt}$ = 291 Hz, *o* to Pt),[s7] −163.22 to −163.33 (m, 2F, *m* to Pt), −163.47 (t, $^3J_{FF}$ = 19.1 Hz, 1F, *p* to Pt).

■ Additional NMR data

$PtC_{28}Pt$ (δ/ppm, $CDCl_3$, replicate measurement): ^1H (500 MHz) 7.46-7.42 (m, 24H, *o* to

P),[s13] 7.12 (d, $^3J_{HH}$ = 7.8 Hz, 24H, *m* to P),[s13] 2.36 (s, 36H, $C\underline{H}_3$); $^{13}C\{^1H\}$ (126 MHz)[s14] 146.7-144.8 (m, *o* to Pt),[s15] 141.1 (s, *p* to P), 138.1-135.3 (2 m, *p* and *m* to Pt),[s15] 134.1 (virtual t, $^2J_{CP}$ = 6.4 Hz, *o* to P), 128.8 (virtual t, $^3J_{CP}$ = 5.6 Hz, *m* to P), 126.7 (virtual t, $^1J_{CP}$ = 30.4 Hz, *i* to P), 111.21 (br s, Pt$\underline{C}$≡C), 94.71 (s, PtC≡$\underline{C}$), 67.69, 66.46, 65.22, 64.42, 63.79, 63.24, 62.72, 62.14, 61.43, 60.52, 59.52, 56.52 (12 s, PtC≡C($\underline{C}$≡$\underline{C}$)$_6$), 21.3 (s, $\underline{C}H_3$ *p* to P).

Crystallography

A. CH_2Cl_2 solution of **PtC$_{26}$Si** was layered with MeOH and kept at ≤ 8 °C. After 2 d, orange plates were collected. Cell parameters were determined from 45 data frames taken at widths of 1° and refined with 9811 reflections using APEX3.[s16] Data were corrected for Lorentz and polarization factors, and (using SADABS)[s17] crystal decay and absorption effects. Systematic reflection conditions and statistical tests suggested the space group *P*-1, which was confirmed by SHELXT. [s17] Hydrogen atom positions were placed in idealized positions and refined using a riding model. All non-hydrogen atoms were refined anisotropically. A molecule of CH_2Cl_2 was located; elongated ellipsoids (C1s, Cl2s, Cl3s) suggested disorder, which was successfully modeled between two positions with occupancies of 0.49 and 0.51. Appropriate restraints were used to keep bond distances, bond angles, and thermal ellipsoids chemically meaningful. The absence of additional symmetry and voids was confirmed using PLATON (ADDSYM).[s18] The structure was refined (weighted least squares refinement on F^2) to convergence.[s18,s20]

B. A CH_2Cl_2/hexanes solution of **PtC$_{20}$Pt** was allowed to slowly evaporate at room temperature. After several days, brown plates were collected. Cell parameters were determined from 45 data frames taken at widths of 1° and refined with 9806 reflections using APEX3.[s16] Data were corrected for Lorentz and polarization factors, and (using SADABS)[s17] crystal decay and absorption effects. Systematic reflection conditions and statistical tests suggested the space group $P2_1$/c. Hydrogen atom positions were placed in idealized positions and refined using a riding model. All non-hydrogen atoms were refined anisotropically. A molecule of CH_2Cl_2 was located; elongated ellipsoids suggested disorder, which was successfully modeled between two positions with an occupancies of 0.73 and 0.27. Appropriate restraints were used to keep bond distances, bond angles,

and thermal ellipsoids chemically meaningful. The absence of additional symmetry and voids was confirmed using PLATON (ADDSYM).[s18] The structure was refined (weighted least squares refinement on F^2) to convergence.[s18,s20]

C. A CH_2Cl_2/hexanes solution of **$PtC_{24}Pt$** was allowed to slowly evaporate at room temperature. After several days, orange blocks were collected. Cell parameters were determined from 45 data frames taken at widths of 1° and refined with 9770 reflections using APEX3.[s16] Data were corrected for Lorentz and polarization factors, and (using SADABS)[s17] crystal decay and absorption effects. Systematic reflection conditions and statistical tests suggested the space group *P*-1. Hexane and CH_2Cl_2 molecules were located; elongated and unusual ellipsoids of the latter suggested disorder and partial occupancies. This was successfully modeled between two positions with occupancies of 0.60 and 0.25, corresponding to **$PtC_{24}Pt$**$\cdot(C_6H_{14})_2\cdot(CH_2Cl_2)_{1.7}$. Appropriate restraints were used to keep bond distances, bond angles, and thermal ellipsoids chemically meaningful. The absence of additional symmetry and voids was confirmed using PLATON (ADDSYM).[s18] The structure was refined (weighted least squares refinement on F^2) to convergence,[s18,s20] and exhibited an inversion center at the midpoint of the C_{24} linkage.

Additional Figures

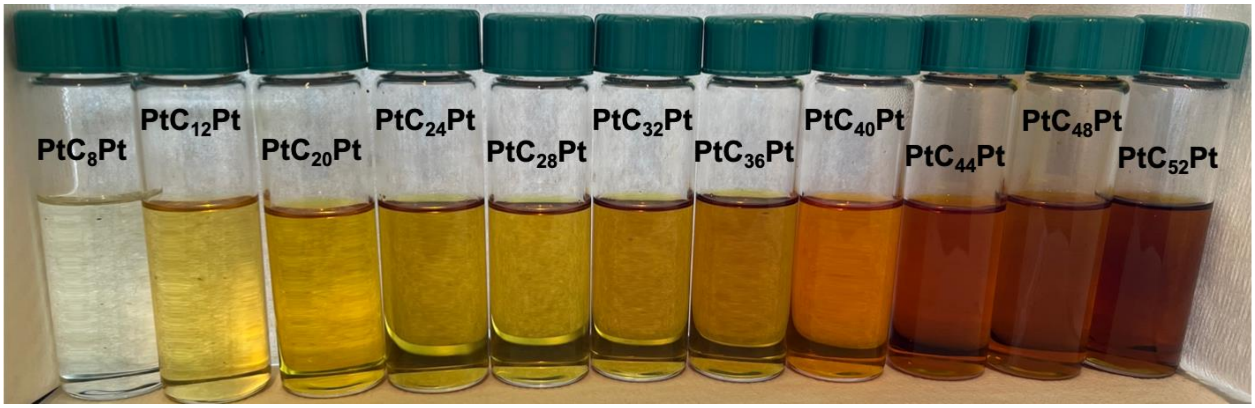

Figure s1. Photographs of solutions of **PtC_xPt** (~ 4×10^{-5} M in CH_2Cl_2).

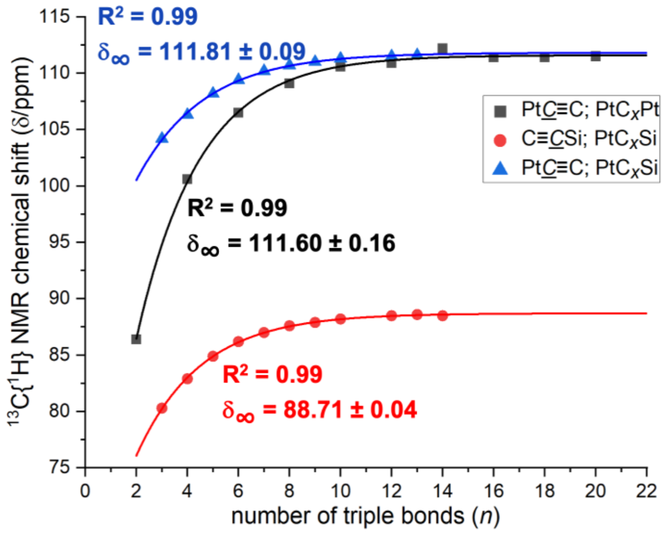

Figure s2. Extrapolation of the Pt$\underline{C}$≡C or C≡$\underline{C}$Si ^{13}C{^{1}H} NMR chemical shifts of **PtC$_x$Si** or **PtC$_x$Pt** (Tables 1 and 2, $CDCl_3$) to $x = \infty$ using the Meier equation: $\delta_n = \delta_\infty - (\delta_\infty - \delta_1)e^{-k(n-1)}$. Due to the change of solvent, **PtC$_{52}$Pt** is omitted.

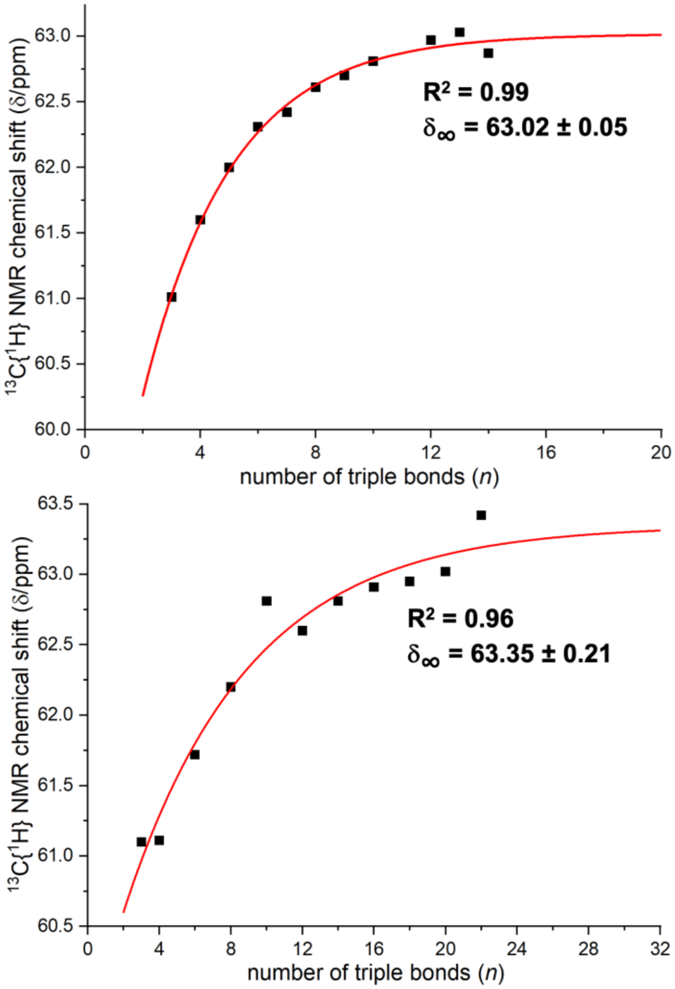

Figure s3. Extrapolation of the average ^{13}C{^{1}H} NMR chemical shifts of all sp carbon atoms of **PtC$_x$Si** (top) or **PtC$_x$Pt** (bottom) (Tables 1 and 2, $CDCl_3$), but omitting the Pt$\underline{C}$≡$\underline{C}$ and $\underline{C}$≡$\underline{C}$Si signals, to $x = \infty$ using the Meier equation: $\delta_n = \delta_\infty - (\delta_\infty - \delta_1)e^{-k(n-1)}$. Due to the change of solvent, **PtC$_{52}$Pt** is omitted.

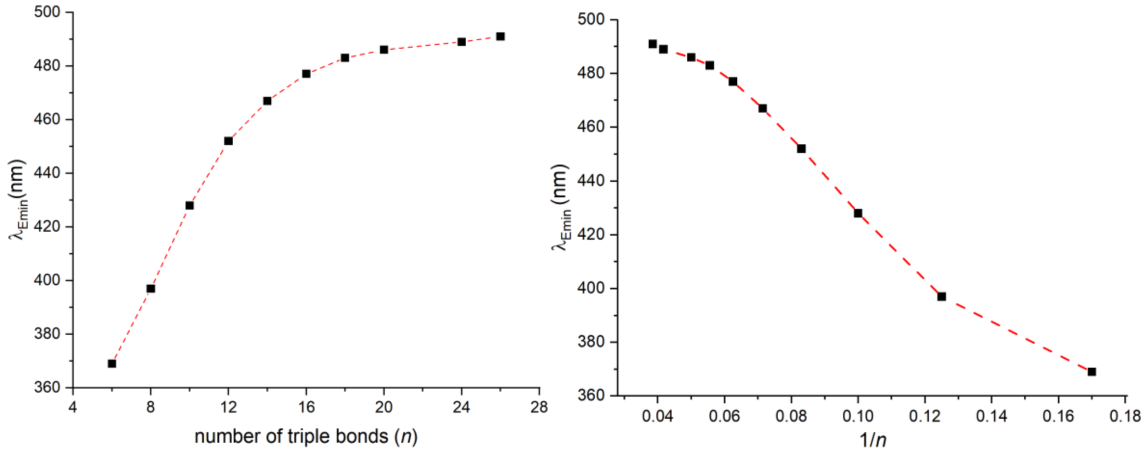

Figure s4. Alternatives to the Meier plot (Figure 12) for visualizing the dependence of the longest wavelength UV-visible bands (λ_{Emin}) of **PtC$_x$Pt** upon the number of triple bonds *n*.

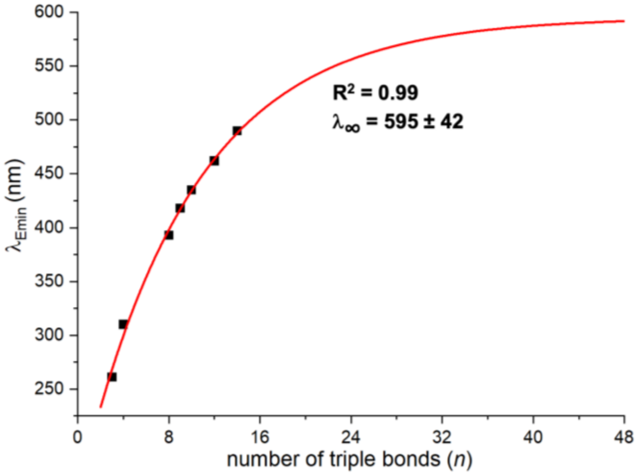

Figure s5. Extrapolation of the λ_{Emin} values (longest wavelength UV-visible absorption) of **PtC$_x$Si** ($x \geq 6$) to $x = \infty$ using the Meier equation: $\delta_n = \delta_\infty - (\delta_\infty - \delta_1)e^{-k(n-1)}$.

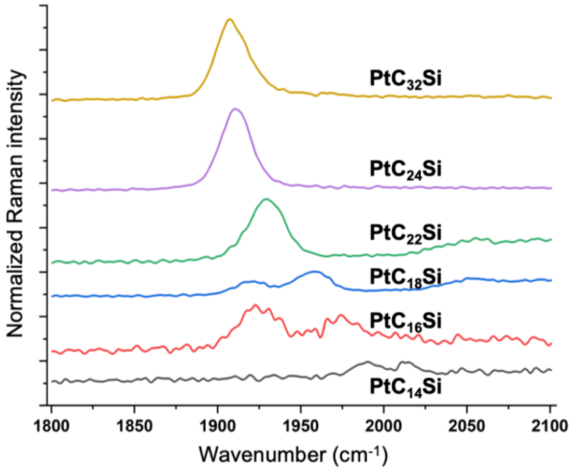

Figure s6. Raman spectra of **PtC$_x$Si** (~1mM CH_2Cl_2 solution) showing peaks (cm^{-1}) at 1907 (**PtC$_{32}$Si**), 1911 (**Pt-C$_{24}$Si**), 1930 (**PtC$_{22}$Si)**, 1959, 2055, 2179 (**PtC$_{18}$Si**); ca. 1975, ca. 1924 (**PtC$_{16}$Si**), 1990, 2078 (**PtC$_{14}$Si**). Spectra of the unsymmetrically substituted polyynes **[BF]C$_x$TIPS** (x = 10, 8, 6, 5, 4, 3, 2) also show multiple bands.[s21]

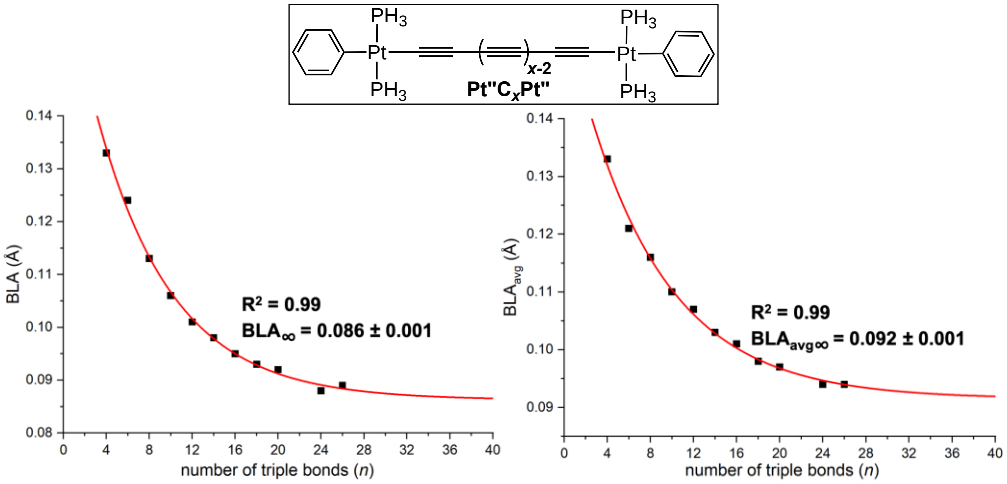

Figure s7. Extrapolation of the computed BLA (left) and BLA(avg) (right) of **Pt"C$_x$Pt"** to $x = \infty$ using the Meier equation: $y_n = y_\infty - (y_\infty - y_1)e^{-k(n-1)}$.

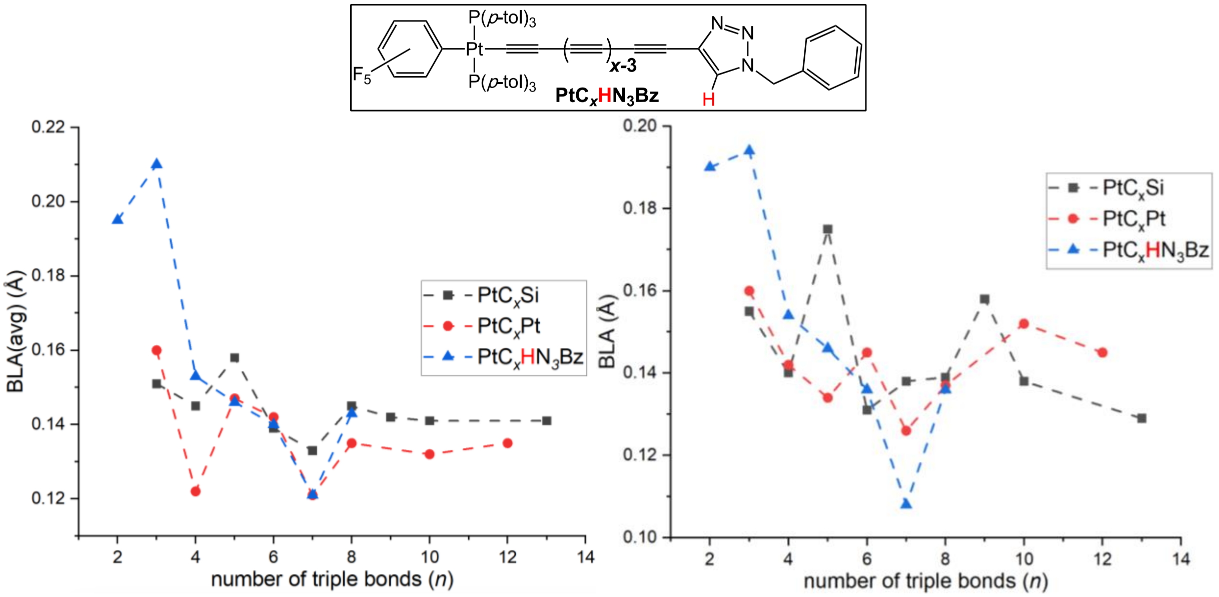

Figure s8. Plots showing the absence of obvious relationships involving crystallographic BLA(avg) (left) and BLA (right) parameters and the number of triple bonds in **PtC$_x$Pt**, **PtC$_x$Si**, and **PtC$_x$HN$_3$Bz**. Apart from **PtC$_{10}$Pt** and **PtC$_{14}$Pt** (unpublished structures), data are from this work and references s2, s3, and s10. All values are provided in Table s10. For **PtC$_x$HN$_3$Bz**, the index x represents the number of sp carbon atoms, as opposed to including the two contiguous sp^2 carbon atoms as done in reference s3.

Additional Tables

Table s1. IR and $^{31}P\{^1H\}$ NMR (202 MHz, $CDCl_3$) data for **PtC$_x$Si** and **PtC$_x$Pt**.

complex	IR $\nu_{C\equiv C}$ (cm^{-1}, powder film)	$^{31}P\{^1H\}$ NMR (δ/ppm) [$^1J_{PPt}$, Hz][a]
**PtC$_6$Si**[b]	2150/2023 (s/m)	17.90 [2636][b]
**PtC$_8$Si**[b]	2131/2088/2065/2015 (m/s/sh/w)	17.90 [2624][b]
**PtC$_{10}$Si**[c]	2166/2142/2065/2006 (m/w/s/s)	18.00 [2610]
**PtC$_{12}$Si**[c]	2145/2118/2027/2000 (m/w/s/m)	18.00 [2608]
**PtC$_{14}$Si**[c]	2120/2064/2016/1987 (m/w/m/s)	18.03 [2601]
**PtC$_{16}$Si**[c]	2158/2133/2061/2046/2004/1967 (w/w/m/w/w/s)	18.02 [2602]
**PtC$_{18}$Si**[c]	2166/2141/2070/2035/2001/1951 (w/m/s/m/w/s)	18.04 [2602]
**PtC$_{20}$Si**[c]	2122/2108/2046/1985/1935 (w/w/m/w/s)	18.03 [2602]
**PtC$_{22}$Si**	2122/2108/2046/1985/1935 (w/w/m/w/s)	18.02 [2588]
**PtC$_{24}$Si**	2141/2083/2012/1994/1917 (m/m/s/s/s)	18.05 [2588]
**PtC$_{26}$Si**	2127/2079/2039/2008/1994/1923 (w/w/w/m/m/s)	18.04 [2598]
**PtC$_{28}$Si**	2116/2056/1981/1942/1906 (m/m/s/m/s)	18.05 [2590]
**PtC$_{32}$Si**	2136/2090/2036/2016/1969/1907 (w/m/m/m/s/s)	18.05 [2603]
**PtC$_4$Pt**	none observed	16.30 [2713]
**PtC$_6$Pt**	none observed	17.20 [2683]
**PtC$_8$Pt**[b]	2152/2011 (s/m)	17.60 [2654][b]
**PtC$_{12}$Pt**[b]	2127/2088/1992 (m/s/m)	17.70 [2622][b]
**PtC$_{16}$Pt**[b]	2154/2088/2054/1984 (w/w/s/m)	18.00 [2609][b]
**PtC$_{20}$Pt**[d]	2158/2142/2053/2023/1988 (w/w/m/s/s)	17.80 [2609]
**PtC$_{24}$Pt**[d]	2138/2115/2065/2003/1980/1918 (m/w/w/s/s/w)	17.80 [2602]
**PtC$_{28}$Pt**	2150/2108/2091/2041/1996/1953/1900 (w/w/w/w/w/s/m)[e]	18.05 [2604]
**PtC$_{32}$Pt**	2147/2085/2070/1936/1898 (w/m/m/s/s)	18.06 [2605]
**PtC$_{36}$Pt**	2134/2050/1989/1922/1889 (w/m/w/s/s)	18.04 [2604]
**PtC$_{40}$Pt**	2113/2031/1977/1912 (w/m/w/s)	18.04 [2600]
**PtC$_{44}$Pt**	2150/2022/1986/1961 (w/w/w/s)	18.03 [2604]
**PtC$_{48}$Pt**	2154/2017/1990/1963 (w/w/w/s)	18.02 [2606]
**PtC$_{52}$Pt**	2160/2063/1992/1967 (w/w/w/s)	18.04 [2604]

[a]The coupling refers to a satellite (^{195}Pt = 33.8%). [b]Data at 162 MHz from reference s10. [c]Data from reference s3. [d]Data from reference s2. [e]Data from an independently prepared sample (replicability check): 2147/2110/2091/2043/1952/1900 (w/w/w/w/s/m).

Table s2. Thermal stability data (°C) for **PtC$_x$Si** and **PtC$_x$Pt**.

complex	TGA, onset mass loss (T_i)[a]	DSC $T_i/T_e/T_p/T_c/T_f$[a]	decomposition (onset) capillary thermolysis[b]
**PtC$_6$Si**[c]	205	154/156/157/159/160[e]	249[d]
**PtC$_8$Si**[c]	198	155/165/170/174/180[e]	115[d]
**PtC$_{10}$Si**[g]	197	121/132/149/159/164[e]	105[d,f]
**PtC$_{12}$Si**[g]	185	120/165/184/195/–[e]	135[d,f]
**PtC$_{14}$Si**[g]	167	135/150/165/170/164[e]	50[d,f]
**PtC$_{16}$Si**[g]	164	103/118/137/152/178[e]	83[h]
**PtC$_{18}$Si**[g]	160	56/58/63/67/69[i]<br>89/108/123/135/146[e]	99[d,i]
**PtC$_{20}$Si**[g]	139	50/54/64/72/75[i]<br>80/100/115/132/140[e]	61[d]
**PtC$_{22}$Si**	–	–	71[d,j]
**PtC$_{24}$Si**	153	93/115/132/148/160[e]	89[d,j]
**PtC$_{26}$Si**	178	79/109/130/148/180[e]	80[d,j]
**PtC$_{28}$Si**	178	–	68[d,j]
**PtC$_4$Pt**[c]	332	110/116/129/140/160[e]	260[d]
**PtC$_6$Pt**[c]	251	179/184/190/–/–[i]	240[j]
**PtC$_8$Pt**[c]	252	155/162/167/175/180[e]	234[d]
**PtC$_{12}$Pt**[c]	270	150/155/156/158/160[e]	288[d]
**PtC$_{16}$Pt**[c]	270	–	270[k]
**PtC$_{20}$Pt**[l]	236	162/177/240/240/240[e]	140[f]/170[d]
**PtC$_{24}$Pt**[l]	230	127/159/177/195/230[e]	120[f]/160[d]
**PtC$_{28}$Pt**	167	114/131/161/169/169[e]	81[d]
**PtC$_{32}$Pt**	170	104/106/125/126/136[i]<br>136/143/168/169/170[e]	75[d]
**PtC$_{36}$Pt**	130	94/–/–/–/–[e]	80[d]
**PtC$_{40}$Pt**	156	104/134/143/151/160[e]	76[d]
**PtC$_{44}$Pt**	–	–	75[d]
**PtC$_{48}$Pt**	–	–	73[d]
**PtC$_{52}$Pt**	–	–	71[d]

[a]These terms are defined in reference s1. [b]Sealed; conventional melting point apparatus. [c]Data from reference s10. [d]Decomposition without any liquefaction under 400 °C. [e]Exotherm. [f]Blackening begins. [g]Data from reference s3 (the decomposition temperatures are erroneously reported therein as from open capillaries). [h]Liquefaction at 121 °C. [i]Endotherm. [j]At this temperature, the sample was black; however, the deep color of the sample made it impossible to assign an onset of darkening. [k]Melting at 189 °C. [l]Data from reference s2.

Table s3. ^{1}H NMR data for **PtC$_x$Si** (δ/ppm; J/Hz; 500 MHz/$CDCl_3$).

complex	CH *ortho* to P[a] (m)	CH *meta* to P[a] [d; $^3J_{HH}$]	$C_6H_4C\underline{H}_3$ (s)	$SiCH_2C\underline{H}_3$ [t; $^3J_{HH}$]	$SiC\underline{H}_2CH_3$ [q; $^3J_{HH}$]
**PtC$_6$Si**[b]	7.45[b]	7.10 [7.4]	2.36	0.90 [7.8]	0.50 [7.8]
**PtC$_8$Si**[b]	7.45[b]	7.11 [7.8]	2.36	0.95 [7.8]	0.57 [7.8]
**PtC$_{10}$Si**[c]	7.48-7.44	7.12 [7.8]	2.37	0.98 [7.9]	0.62 [7.9]
**PtC$_{12}$Si**[c]	7.45-7.42	7.12 [7.8]	2.37	0.98 [7.9]	0.63 [7.9]
**PtC$_{14}$Si**[c]	7.47-7.43	7.12 [7.8]	2.37	0.99 [7.9]	0.64 [7.9]
**PtC$_{16}$Si**[c]	7.46-7.43	7.12 [7.8]	2.37	1.00 [7.9]	0.65 [7.9]
**PtC$_{18}$Si**[c]	7.46-7.42	7.12 [7.8]	2.37	1.00 [7.9]	0.65 [7.9]
**PtC$_{20}$Si**[c]	7.46-7.42	7.12 [7.8]	2.37	1.00 [7.9]	0.65 [7.9]
**PtC$_{22}$Si**	7.45-7.41	7.12 [7.8]	2.36	0.99 [7.9]	0.65 [7.9]
**PtC$_{24}$Si**	7.46-7.42	7.12 [7.8]	2.37	1.00 [7.9]	0.65 [7.9]
**PtC$_{26}$Si**	7.46-7.42	7.12 [7.8]	2.37	1.00 [7.9]	0.65 [7.9]
**PtC$_{28}$Si**	7.45-7.41	7.12 [7.8]	2.36	0.99 [7.9]	0.65 [7.9]
**PtC$_{32}$Si**	7.45-7.41	7.12 [7.8]	2.36	0.99 [7.9]	0.65 [8.0]

[a]The aryl ^{1}H NMR signal that is a doublet is assigned to the CH group *meta* to phosphorus, and that that is a multiplet (presumably due to coupling to additional nuclei) is assigned to the *ortho* CH group. [b]Data at 400 MHz from reference s10; in this study only the center of the multiplet for CH group *ortho* to phosphorus was recorded. [c]Data from reference s3.

Table s4. ^{1}H NMR data for **PtC$_x$Pt** (δ/ppm; J/Hz; 500 MHz/$CDCl_3$).

complex	CH *ortho* to P[a] (m)	CH *meta* to P[a] [d; $^3J_{HH}$]	$C_6H_4C\underline{H}_3$ (s)
**PtC$_4$Pt**[b]	7.43[b]	6.88 [7.7]	2.28
**PtC$_6$Pt**[b]	7.44[b]	7.06 [7.8]	2.29
**PtC$_8$Pt**[b]	7.43[b]	7.06 [7.8]	2.33
**PtC$_{12}$Pt**[b]	7.43[b]	7.09 [7.8]	2.34
**PtC$_{16}$Pt**[b]	7.44[b]	7.10 [7.8]	2.35
**PtC$_{20}$Pt**[c]	7.46-7.42	7.12 [7.7]	2.36
**PtC$_{24}$Pt**[c]	7.46-7.42	7.12 [7.7]	2.36
**PtC$_{28}$Pt**	7.45-7.42	7.12 [7.8]	2.36
**PtC$_{32}$Pt**	7.45-7.41	7.12 [7.8]	2.36
**PtC$_{36}$Pt**	7.45-7.42	7.12 [7.8]	2.36
**PtC$_{40}$Pt**	7.45-7.41	7.11 [7.5]	2.36
**PtC$_{44}$Pt**	7.46-7.42	7.12 [7.5]	2.36
**PtC$_{48}$Pt**	7.46-7.42	7.12 [7.5]	2.36
**PtC$_{52}$Pt**	7.50-7.44	7.12 [7.5]	2.37

[a]The aryl ^{1}H NMR signal that is a doublet is assigned to the CH group *meta* to phosphorus, and that that is a multiplet (presumably due to coupling to additional nuclei) is assigned to the *ortho* CH group. [b]Data at 400 MHz from reference s10; in this study only the center of the multiplet for CH group *ortho* to phosphorus was recorded. [c]Data from reference s10.

Table s5. Additional $^{13}C\{^{1}H\}$ NMR data for **PtC$_x$Si** (δ/ppm; *J*/Hz; 126 MHz/$CDCl_3$).

complex	C_6F_5 *ortho* to Pt [dd; $^{1}J_{CF}/^{2}J_{CF}$][a]	C *para* to P (s)	C *ortho* to P [t; $^{2}J_{CP}$][b]	C *meta* to P [t; $^{3}J_{CP}$][b]	C *ipso* to P [t; $^{1}J_{CP}$][b]	$C_6H_4CH_3$ (s)	$SiCH_2CH_3$ (s)	$SiCH_2$ [s, $^{1}J_{CSi}$][c]
**PtC$_6$Si**[d]	146.0 [226/22.0]	140.8	134.2 [6.5]	128.7 [5.5]	127.1 [30.2]	21.3	7.3	4.3
**PtC$_8$Si**[d]	145.7 [223/19.0]	140.9	134.2 [6.5]	128.7 [5.5]	126.9 [30.6]	21.3	7.3	4.2
**PtC$_{10}$Si**[e]	146.0 [226/21.4]	141.2	134.4 [6.4]	128.9 [5.5]	127.1 [30.4]	21.5	7.5	4.3 [56.7]
**PtC$_{12}$Si**[e]	145.9 [224/23.9]	141.2	134.4 [6.3]	128.9 [5.5]	127.0 [30.4]	21.5	7.5	4.3 [56.7]
**PtC$_{14}$Si**[e]	145.9 [223/21.4]	141.2	134.3 [6.4]	128.9 [5.7]	127.0 [30.4]	21.5	7.4	4.2 [57.9]
**PtC$_{16}$Si**[e]	145.9 [227/22.7]	141.3	134.3 [6.3]	128.9 [5.6]	127.0 [30.4]	21.5	7.4	4.2 [56.7]
**PtC$_{18}$Si**[e]	146.0 [226/22.7]	141.3	134.3 [6.4]	128.9 [5.6]	126.9 [30.4]	21.3	7.4	4.2 [56.7]
**PtC$_{20}$Si**[e]	145.9 [223/22.7]	141.3	134.3 [6.4]	128.9 [5.6]	126.9 [30.4]	21.5	7.4	4.2 [56.5]
**PtC$_{22}$Si**	146.7 [223/23.7]	141.3	134.3 [6.4]	128.9 [5.6]	126.9 [30.5]	21.5	7.4	4.2 [56.7]
**PtC$_{24}$Si**	145.9 [223/23.9]	141.3	134.3 [6.4]	128.9 [5.5]	126.9 [30.4]	21.5	7.4	4.2 [56.7]
**PtC$_{26}$Si**	146.9-144.9[f]	141.3	134.3 [6.3]	128.9 [5.6]	126.9 [30.5]	21.5	7.4	4.2 [56.7]
**PtC$_{28}$Si**	146.6-144.8[f]	141.3	134.1 [6.4]	128.8 [5.6]	126.7 [30.5]	21.4	7.3	4.0 [57.0]
**PtC$_{32}$Si**	–[f]	141.3	134.3 [6.4]	128.9 [5.6]	126.9 [30.4]	21.5	7.4	4.2 [57.0]

[a]The *ipso* C_6F_5 signal was not observed, and the *meta* and *para* C_6F_5 signals were overlapping multiplets at 135-140 ppm. [b]These are virtual triplets; the *J* values represent the *apparent* couplings between adjacent peaks. [c]This coupling represents a satellite (d, ^{29}Si = 4.67%) and is not reflected in the peak multiplicity. [d]Data from reference s10 at 101 MHz. [e]Data from reference s3. [f]The signal/noise was not sufficient to resolve the signal or couplings.

Table s6. Additional $^{13}C\{^{1}H\}$ NMR data for **PtC$_x$Pt** (δ/ppm; *J*/Hz; 126 MHz/$CDCl_3$).

complex	$\underline{C}_6F_5$ *ortho* to Pt [dd; $^1J_{CF}/^2J_{CF}$][a]	$\underline{C}$ *para* to P (s)	$\underline{C}$ *ortho* to P [t; $^2J_{CP}$][b]	$\underline{C}$ *meta* to P [t; $^3J_{CP}$][b]	$\underline{C}$ *ipso* to P [t; $^1J_{CP}$][b]	$C_6H_4\underline{C}H_3$ (s)
**PtC$_4$Pt**[c]	145.9 [220/22.0]	139.7	134.4 [6.3]	128.1 [5.4]	128.1 [29.7]	21.3
**PtC$_6$Pt**[c]	145.7 [226/22.0]	140.4	134.2 [6.5]	128.7 [5.5]	126.8 [30.5]	21.3
**PtC$_8$Pt**[c]	145.7 [225/22.0]	140.6	134.2 [6.2]	128.6 [5.4]	127.2 [30.2]	21.3
**PtC$_{12}$Pt**[c]	145.7 [226/22.0]	140.9	134.2 [6.5]	128.7 [5.5]	126.9 [29.4]	21.3
**PtC$_{16}$Pt**[c]	145.7 [226/22.0]	141.0	134.2 [6.5]	128.7 [5.5]	126.8 [30.5]	21.3
**PtC$_{20}$Pt**[d]	–[e]	141.3	134.3 [6.6]	128.9 [5.5]	126.9 [30.5]	21.5
**PtC$_{24}$Pt**[d]	–[e]	141.1	134.3 [6.6]	128.9 [5.4]	126.8 [30.2]	21.5
**PtC$_{28}$Pt**	147.0-145.0[e]	141.3	134.3 [6.4]	128.9 [5.6]	126.9 [30.4]	21.5
**PtC$_{32}$Pt**	146.7-144.7[e]	141.3	134.3 [6.4]	128.9 [5.6]	126.7 [30.5]	21.4
**PtC$_{36}$Pt**	146.7-144.7[e]	141.3	134.3 [6.4]	128.9 [5.6]	126.7 [30.5]	21.5
**PtC$_{40}$Pt**	146.7-144.7[e]	141.1	134.1 [6.6]	128.8 [5.3]	126.9 [30.4]	21.5
**PtC$_{44}$Pt**	146.7-144.7[e]	141.3	134.3 [6.6]	128.9 [5.4]	126.9 [30.4]	21.5
**PtC$_{48}$Pt**	–[e]	141.3	134.1[e]	128.9 [5.4]	126.7 [30.5]	21.5
**PtC$_{52}$Pt**	–[e]	141.3	134.4[e]	128.8 [5.6]	126.8[e]	21.5

[a]The *ipso* C_6F_5 signal was not observed, and the *meta* and *para* C_6F_5 signals were overlapping multiplets at 135-139 ppm. [b]These are virtual triplets; the *J* values represent the *apparent* couplings between adjacent peaks. [c]Data from reference s10 at 101 MHz. [d]Data from reference s2 at 126 MHz. [e]The signal/noise was not sufficient to resolve the signal or couplings.

Table s7. Summary of Crystallographic Data.

	**$PtC_{26}Si$**$\cdot(CH_2Cl_2)$	**$PtC_{20}Pt$**$\cdot(CH_2Cl_2)$	**$PtC_{24}Pt$**$\cdot(C_6H_{14})_2\cdot(CH_2Cl_2)_{1.7}$
empirical formula	$C_{81}H_{59}Cl_2F_5P_2PtSi$	$C_{117}H_{86}Cl_2F_{10}P_4Pt_2$	$C_{133.7}H_{115.4}Cl_{3.4}F_{10}P_4Pt_2$
formula weight	1483.30	2266.81	2546.64
temperature [K]	110.0	110.0	110.0
diffractometer	BRUKER Quest	BRUKER Quest	BRUKER Venture
wavelength [Å]	0.71073	0.71073	1.54178
crystal system	triclinic	monoclinic	triclinic
space group	*P*–1	*P2₁/c*	*P*–1
unit cell dimensions			
a [Å]	12.5294(15)	22.951(2)	10.8825(4)
b [Å]	12.8742(16)	16.6804(14)	16.3618(7)
c [Å]	21.478(3)	28.229(2)	18.4427(7)
α [°]	90.420(2)	90	64.743(2)
β [°]	93.709(2)	113.118(3)	82.241(2)
γ [°]	99.751(2)	90	81.019(2)
volume [$Å^3$]	3406.8(7)	9939.5(15)	9939.5(15)
Z	2	4	1
ρ_{calc} [Mg/m^3]	1.446	1.515	1.446
μ [nm^{-1}]	2.261	2.999	6.175
F(000)	14924	4512	1281
crystal size [mm]	0.204 × 0.187 × 0.014	0.467 × 0.441 × 0.035	0.281 × 0.178 × 0.018
Θ range [°]	1.850 to 20.788	1.905 to 23.499	2.657 to 70.529
index ranges	$-12 \leq h \leq 12$	$-25 \leq h \leq 25$	$-13 \leq h \leq 12$
	$-12 \leq k \leq 12$	$-18 \leq k \leq 18$	$-19 \leq k \leq 20$
	$-21 \leq l \leq 21$	$-31 \leq l \leq 31$	$-22 \leq l \leq 22$
reflections collected	43447	76069	80507
independent reflections	7097 [R(int) = 0.0702]	14702 [R(int) = 0.0812]	11141 [R(int) = 0.0467]
data/restraints/parameters	7097/84/866	14702/75/1255	11141/38/729
goodness-of-fit on F^2	1.261	1.081	1.085
final R indices [$I>2\sigma(I)$]	$R1 = 0.0587$, $wR^2 = 0.1164$	$R1 = 0.0376$, $wR^2 = 0.0829$	$R1 = 0.0397$, $wR^2 = 0.0845$
R indices (all data)	$R1 = 0.0658$, $wR^2 = 0.1188$	$R1 = 0.0583$, $wR^2 = 0.0993$	$R1 = 0.0472$, $wR^2 = 0.0912$
largest diff. peak/hole [$eÅ^{-3}$]	1.934 and −2.246	1.433 and −0.947	2.313 and −1.461

Table s8. Crystallographic distances [Å] and angles [°] for **$PtC_{26}Si$**·(CH_2Cl_2).

Pt-C1	1.989(12)	C_{ipso}[a] -Pt-C1	178.4(5)
C1≡C2	1.217(14)	Pt-C1-C2	178.8(9)
C2-C3	1.319(16)	C1-C2-C3	177.4(11)
C3≡C4	1.210(15)	C2-C3-C4	177.7(12)
C4-C5	1.382(17)	C3-C4-C5	170.8(12)
C5≡C6	1.211(15)	C4-C5-C6	175.0(12)
C6-C7	1.348(18)	C5-C6-C7	174.8(13)
C7≡C8	1.209(16)	C6-C7-C8	179.0(13)
C8-C9	1.323(17)	C7-C8-C9	178.6(14)
C9≡C10	1.227(16)	C8-C9-C10	177.6(13)
C10-C11	1.343(18)	C9-C10-C11	175.8(13)
C11≡C12	1.208(16)	C10-C11-C12	177.1(12)
C12-C13	1.358(17)	C12-C13-C14	177.5(11)
C13≡C14	1.220(15)	C13-C14-C15	178.6(11)
C14-C15	1.340(17)	C14-C15-C16	178.0(11)
C15≡C16	1.208(15)	C15-C16-C17	177.1(11)
C16-C17	1.342(17)	C16-C17-C18	179.4(12)
C17≡C18	1.196(15)	C17-C18-C19	177.7(11)
C18-C19	1.364(17)	C18-C19-C20	179.7(13)
C19≡C20	1.198(15)	C19-C20-C21	179.0(12)
C20-C21	1.346(17)	C20-C21-C22	178.5(12)
C21≡C22	1.209(15)	C21-C22-C23	179.1(12)
C22-C23	1.361(17)	C22-C23-C24	178.0(12)
C23≡C24	1.201(15)	C23-C24-C25	177.6(11)
C24-C25	1.375(18)	C24-C25-C26	174.7(12)
C25≡C26	1.202(15)	C25-C26-Si	177.1(10)
C26-Si	1.837(12)		
Pt-C_{ipso}[a]	2.055(10)		
avg. C≡C	1.209	avg. bond angle	177.4
avg. C-C	1.350	from Pt to Si	
BLA(avg)[b]	0.141	π stacking	3.605/3.675
BLA[c]	0.129	distances[d]	
sum, bond lengths	35.371	stacking angle[e]	158.0
from Pt to Si			
Pt···Si	35.743	C_{ipso}-Pt-P-$C_{ipso'}$[f]	–4.90, –26.64

[a]C_{ipso} is the ligating carbon of the C_6F_5 ring. [b]BLA(avg) = (avg. C-C) – (avg. C≡C). [c]BLA = absolute value of the length of the central carbon-carbon bond (C≡C for *n* = odd; C-C for *n* = even; *n* = number of C≡C units) minus the average length of the two adjacent bonds. [d]Distances between the centroids of the C_6F_5 and two $C_6H_4CH_3$ rings. [e]The angle of the centroids of the three rings in *d*. [f]When the torsion angle is 0°, the C_6F_5 and $C_6H_4CH_3$ groups are positioned directly above/below each other (although some independent tilting remains possible).

Table s9. Crystallographic distances [Å] and angles [°] for solvates of **$PtC_{20}Pt$** and **$PtC_{24}Pt$**.

	**$PtC_{20}Pt$**$\cdot(CH_2Cl_2)$	**$PtC_{24}Pt$**$\cdot(C_6H_{14})_2\cdot(CH_2Cl_2)_{1.7}$[a]
Pt1-C1	1.984(7)	1.988(4)
C1≡C2	1.232(9)	1.211(6)
C2-C3	1.337(9)	1.354(6)
C3≡C4	1.226(9)	1.222(7)
C4-C5	1.345(10)	1.350(7)
C5≡C6	1.227(9)	1.214(7)
C6-C7	1.345(10)	1.354(7)
C7≡C8	1.201(9)	1.224(7)
C8-C9	1.352(10)	1.349(7)
C9≡C10	1.205(9)	1.211(7)
C10-C11	1.363(10)	1.362(7)
C11≡C12	1.216(9)	1.210(7)
C12-C12	–	1.355(10)
C12-C13	1.350(10)	–[a]
C13≡C14	1.216(9)	–[a]
C14-C15	1.358(9)	–[a]
C15≡C16	1.207(9)	–[a]
C16-C17	1.343(9)	–[a]
C17≡C18	1.217(9)	–[a]
C18-C19	1.348(9)	–[a]
C19≡C20	1.218(9)	–[a]
Pt2-C20	1.998(7)	–[a]
Pt1-C_{ipso}[b]	2.065(6)	–[a]
Pt2-C_{ipso}[b]	2.058(7)	2.064(4)
avg. C≡C	1.217	1.215
avg. C-C	1.349	1.354
BLA(avg)[c]	0.132	0.139
BLA[d]	0.152	0.145
sum, bond lengths from Pt to Pt	28.288	33.157
Pt···Pt	28.257	33.453
C_{ipso}[b]-Pt-C1	178.4(2)	176.73(16)
Pt-C1-C2	174.1(6)	174.6(4)
C1-C2-C3	178.2(7)	176.7(5)
C2-C3-C4	176.8(8)	178.5(5)
C3-C4-C5	177.7(8)	176.2(6)
C4-C5-C6	177.1(8)	176.9(6)
C5-C6-C7	176.5(8)	176.4(6)
C6-C7-C8	177.7(8)	176.0(6)
C7-C8-C9	176.7(8)	178.6(6)
C8-C9-C10	177.8(8)	178.0(7)
C9-C10-C11	177.1(8)	178.5(7)
C10-C11-C12	177.4(8)	178.7(6)

C11-C12-C12	–	179.7(8)
C11-C12-C13	178.2(8)	–[a]
C12-C13-C14	177.8(8)	–[a]
C13-C14-C15	176.9(8)	–[a]
C14-C15-C16	178.4(8)	–[a]
C15-C16-C17	178.5(8)	–[a]
C16-C17-C18	178.8(8)	–[a]
C17-C18-C19	178.9(7)	–[a]
C18-C19-C20	176.9(7)	–[a]
C19-C20-Pt2	174.9(5)	–[a]
avg. bond angle from Pt to Pt	177.4	177.3
π-stacking distances[e]	3.47, 3.80/3.46, 3.80	3.87, 3.91
stacking angle[f]	156.8/155.5	158.1
C_{ipso}-Pt-P-C_{ipso}[g]	–2.5, 12.6/–4.3, 8.7	–0.1, –2.69

[a]Due to the inversion center at the midpoint of the C_{24} chain, the metrical parameters involving Pt2 and C24-C13 are mirrored by those of Pt1 and C1-C12. [b]C_{ipso} is the ligating carbon of the C_6F_5 ring. [c]BLA(avg) = (avg. C-C)-(avg. C≡C). [d]BLA = absolute value of the length of the central carbon-carbon bond (C≡C for *n* = odd; C-C for *n* = even; *n* = number of C≡C units) minus the average length of the two adjacent bonds. [e]Distances between the centroids of the C_6F_5 and two $C_6H_4CH_3$ rings. [f]The angle of the centroids of the three rings in *e*. [g]When the torsion angle is 0°, the C_6F_5 and $C_6H_4CH_3$ groups are positioned directly above/below each other (although some independent tilting remains possible).

Table s10. Experimental BLA data for crystallographically characterized complexes. Plots are given in Figure s8, together with the structure of **PtC$_x$HN$_3$Bz**.

complex	BLA[a]	BLA(avg)[b]	complex	BLA[a]	BLA(avg)[b]
**PtC$_6$Si**[c]	0.155	0.151	**PtC$_{12}$HN$_3$Bz**[d]	0.146	0.146
**PtC$_8$Si**[c]	0.140	0.145	**PtC$_{14}$HN$_3$Bz**[d]	0.136	0.140
**PtC$_{10}$Si**[d]	0.175	0.158	**PtC$_{16}$HN$_3$Bz**[d]	0.108	0.121
**PtC$_{12}$Si**[d]	0.131	0.139	**PtC$_{18}$HN$_3$Bz**[d]	0.136	0.143
**PtC$_{14}$Si**[d]	0.138	0.133	**PtC$_6$Pt**[e]	0.164	0.160
**PtC$_{16}$Si**[d]	0.139	0.145	**PtC$_8$Pt**[c]	0.142	0.122
**PtC$_{18}$Si**[d]	0.158	0.142	**PtC$_{10}$Pt**[f]	0.134	0.147
**PtC$_{20}$Si**[d]	0.138	0.141	**PtC$_{12}$Pt**[c]	0.145	0.142
**PtC$_{26}$Si**[g]	0.129	0.141	**PtC$_{14}$Pt**[f]	0.126	0.121
**PtC$_6$HN$_3$Bz**[h]	0.190	0.195	**PtC$_{16}$Pt**[c]	0.137	0.135
**PtC$_8$HN$_3$Bz**[d]	0.194	0.210	**PtC$_{20}$Pt**[g]	0.152	0.132
**PtC$_{10}$HN$_3$Bz**[d]	0.154	0.153	**PtC$_{24}$Pt**[g]	0.145	0.135

[a]BLA = absolute value of the length of the central carbon-carbon bond (C≡C for *n* = odd; C-C for *n* = even (*n* = number of C≡C units)) minus the average length of the two adjacent bonds. [b]BLA(avg) = (avg C-C) – (avg C≡C). [c]Data from reference s4. [d]Data from reference s3. [e]Data from reference s23. [f]Unpublished structure: Dey Baksi, S.; Bhuvanesh, N. Texas A&M University. [g]This work. [h]Data from reference s22.

Table s11. Additional data relating to Meier equation plots.

Parameters from the equation $\nu_n = \nu_\infty - (\nu_\infty - \nu_1)e^{-k(n-1)}$ in Figure 11.

variable	**PtC$_x$Pt**
ν_1	2160 ± 115
k	0.18 ± 0.06

Parameters from the equation $\lambda_n = \lambda_\infty - (\lambda_\infty - \lambda_1)e^{-k(n-1)}$ in Figures 12 and s5.

variable	**PtC$_x$Pt**	**PtC$_x$Si**
λ_1	242 ± 09	194 ± 69
k	0.14 ± 0.02	0.13 ± 0.01

Parameters from the equation $\delta_n = \delta_\infty - (\delta_\infty - \delta_1)e^{-k(n-1)}$ in Figure s2.

variable	**PtC$_x$Si** (Pt<u>C</u>≡C)	**PtC$_x$Si** (C≡<u>C</u>Si)	**PtC$_x$Pt** (Pt<u>C</u>≡C)
δ_1	95.3 ± 0.6	69.9 ± 0.4	74.0 ± 0.8
k	0.38 ± 0.01	0.40 ± 0.01	0.40 ± 0.01

Parameters from the equation $\delta_n = \delta_\infty - (\delta_\infty - \delta_1)e^{-k(n-1)}$ in Figure s3.

variable	**PtC$_x$Si**	**PtC$_x$Pt**
δ_1	59.20 ± 0.02	60.20 ± 0.03
k	0.33 ± 0.03	0.17 ± 0.04

Parameters from the equation $y_n = y_\infty - (y_\infty - y_1)e^{-k(n-1)}$ in Figure s7.

variable	**Pt"C$_x$Pt"**
BLA_1	0.156 ± 0.003
k	0.318 ± 0.020
$BLA(avg)_1$	0.144 ± 0.002
k	0.254 ± 0.019

References

(s1) Data were treated as recommended by Cammenga, H. K.; Epple, M. Basic Principles of Thermoanalytical Techniques and Their Applications in Preparative Chemistry. *Angew. Chem., Int. Ed. Engl.* **1995**, *34*, 1171-1187; Grundlagen der Thermischen Analysetechniken und ihre Anwendungen in der praparativen Chemie. *Angew. Chem.* **1995**, *107*, 1284-1301.

(s2) Weisbach, N.; Kuhn, H.; Amini, H.; Ehnbom, A.; Hampel, F.; Reibenspies, J. H.; Hall, M. B.; Gladysz, J. A. Triisopropylsilyl (TIPS) Alkynes as Building Blocks for Syntheses of Platinum Triisopropylsilylpolyynyl and Diplatinum Polyynediyl Complexes. *Organometallics* **2019**, *38*, 3294-3310.

(s3) Amini, H.; Weisbach, N.; Gauthier, S.; Kuhn, H.; Bhuvanesh, N.; Hampel, F.; Reibenspies, J. H.; Gladysz, J. A. Trapping of Terminal Platinapolyynes by Copper(I) Catalyzed Click Cycloadditions; Probes of Labile Intermediates in Syntheses of Complexes with Extended sp Carbon Chains, and Crystallographic Studies. *Chem. Eur. J.* **2021**, *27*, 12619-12634.

(s4) Eastmond, R.; Johnson, T. R.; Walton, D. R. M. Silylation as a Protective Method for Terminal Alkynes in Oxidative Couplings. *Tetrahedron* **1972**, *28*, 4601-4616.

(s5) Dembinski, R.; Bartik, T.; Bartik, B.; Jaeger, M.; Gladysz, J. A. Towards Metal-Capped One-Dimensional Carbon Allotropes: Wirelike C_6–C_{20} Polyynediyl Chains That Span Two Redox-Active (η^5-C_5Me_5)Re(NO)(PPh_3) Endgroups. *J. Am. Chem. Soc.* **2000**, *122*, 810-822.

(s6) This signal is assigned by analogy to chemical shift trends established for (*i*-Pr)$_3$Si-(C≡C)$_4$Si(*i*-Pr)$_3$: Eisler, S.; Slepkov, A. D.; Elliott, E.; Luu, T.; McDonald, R.; Hegmann, F. A.; Tykwinski, R. R. Polyynes as a Model for Carbynes: Synthesis, Physical Properties, and Nonlinear Optical Response. *J. Am. Chem. Soc.* **2005**, *127*, 2666-2676.

(s7) This coupling represents a satellite (d; ^{195}Pt = 33.8% or ^{29}Si = 4.67%), and is not reflected in the peak multiplicity given.

(s8) The complex **PtC$_{22}$Si** can also be prepared in comparable overall yield (along with a larger quantity of **PtC$_{14}$Si**) by treating **PtC$_6$Si** with crude **HC$_8$Si** under the conditions of Scheme

(s9) As noted in the main text, the slow addition of the blue Hay catalyst supernatant (from CuCl/TMEDA/acetone) is thought to help avoid byproducts derived from C_2 loss.

(s10) Mohr, W.; Stahl, J.; Hampel, F.; Gladysz, J. A. Synthesis, Structure, and Reactivity of sp Carbon Chains with Bis(phosphine)Pentafluorophenylplatinum Endgroups: Butadiynediyl (C_4) through Hexadecaoctaynediyl (C_{16}) Bridges, and Beyond. *Chem. Eur. J.* **2003**, *9*, 3324c3340.

(s11) For this experiment, the crude **HC$_8$Si** described in the main text was chromatographed (silica gel, hexanes, 3.5 × 30 cm column). The product containing fractions were assayed by silica gel TLC. That of **HC$_8$H** has the lowest R_f value, and this portion of the initially gold band abruptly becomes black and quite warm at some point in the column. An explosion has never occurred, but the silica "lifts" somewhat. Appropriate caution should be exercised, especially on larger scales.

(s12) m/Z; most intense peak of the isotope envelope.

(s13) The aryl ^{1}H NMR signal that appears as a doublet is assigned to the *meta* CH moiety, and that that appears as a multiplet (presumably due to stronger ^{31}P coupling) is assigned to be the *ortho* CH moiety.

(s14) The *ipso* C_6F_5 signal was not observed.

(s15) The expected first order couplings were not resolved due to poor signal/noise ratio.

(s16) *APEX3, Program for Data Collection on Area Detectors.* Bruker AXS Inc., Madison, WI 53711-5373 USA.

(s17) Sheldrick, G. M. *SADABS, Program for Absorption Correction for Data from Area Detector Frames*. Bruker AXS Inc., Madison, WI 53711-5373 USA.

(s18) (a) Sheldrick, G. M. SHELXT – Integrated space-group and crystal structure determination. *Acta Cryst*. **2015**, *A71*, 3-8. (b) Sheldrick, G. M. Crystal structure refinement with *SHELXL*. *Acta Cryst*. **2015**, *C71*, 3-8.

(s19) Spek, A. L. Single-crystal structure validation with the program *PLATON*. *J. Appl. Cryst.* **2003**, *36*, 7-13.

(s20) Dolomanov, O. V, Bourhis, L. J., Gildea, R. J., Howard, J. A. K., Puschmann, H. OLEX2: A Complete Structure Solution, Refinement and Analysis Program. *J. Appl. Cryst*. **2009**, *42*, 339-341.

(s21) (a) Dhindsa. J. S.; Cotterill, E. L.; Buguis, F. L.; Anghel, M.; Boyle, P. D.; Gilroy, J. B. Blending the Optical and Redox Properties of Oligoynes and Boron Difluoride Formazanates. *Angew. Chem., Int. Ed.* **2022**, *61*, e202208502; *Angew. Chem.* **2022**, *134*, e202208502. (b) Unlike symmetrically substituted polyynes, unsymmetrically substituted systems no longer have a center of symmetry, which removes the rule of mutual exclusion for IR and Raman spectra. See also Lucotti, A.; Tommasini, M.; Fazzi, D.; Del Zoppo, M.; Chalifoux, W. A.; Ferguson, M. J.; Zerbi, G.; Tykwinski, R. R. Evidence for Solution-State Nonlinearlity of sp-Carbon Chains Based on IR and Raman Spectroscopy: Violation of Mutual Exclusion. *J. Am. Chem. Soc.* **2009**, *131*, 4239-4244.

(s22) Gauthier, S.; Weisbach, N.; Bhuvanesh, N.; Gladysz, J. A. "Click" Chemistry in Metal Coordination Spheres: Copper (I)- Catalyzed 3+2 Cycloadditions of Benzyl Azide and Platinum Polyynyl Complexes *trans*-$(C_6F_5)(p\text{-tol}_3P)_2Pt(C{\equiv}C)_nH$ (n = 2-6). *Organometallics* **2009**, *19*, 5597-5599.

(s23) Owen, G. R.; Stahl, J.; Hampel, F.; Gladysz, J. A. Coordination-Driven Self Assembly, Structures, and Dynamic Properties of Diplatinum Hexatriynediyl and Butadiynediyl Complexes in which the sp Carbon Chains are Shielded by sp^3 Carbon Chains: Towards Endgroup-Endgroup Interactions. *Chem. Eur. J.* **2008**, *14*, 73– 87.

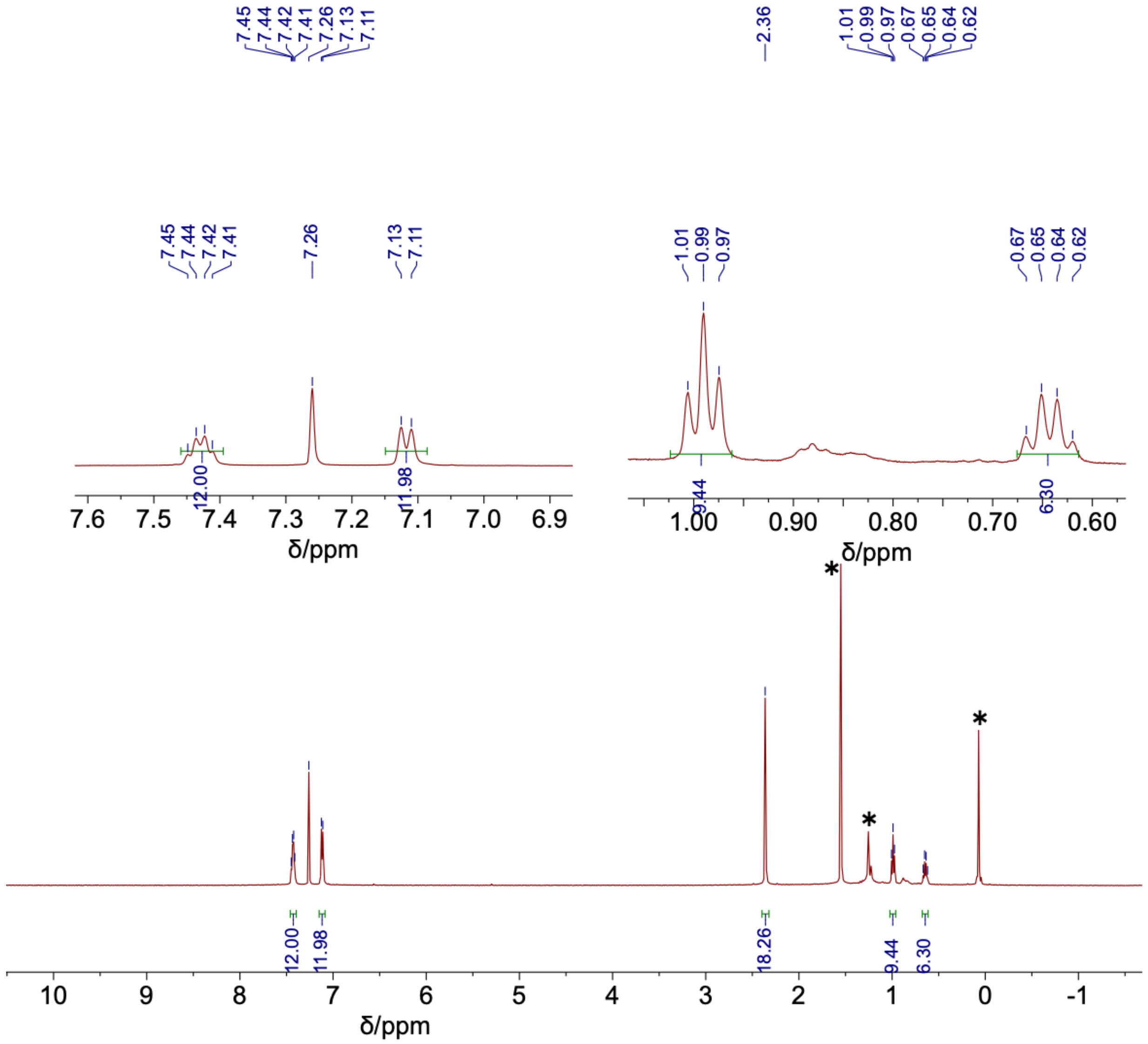

Figure s9. ^{1}H NMR spectrum of **$PtC_{22}Si$** ($CDCl_3$, 500 MHz). * denotes solvent-based impurities.

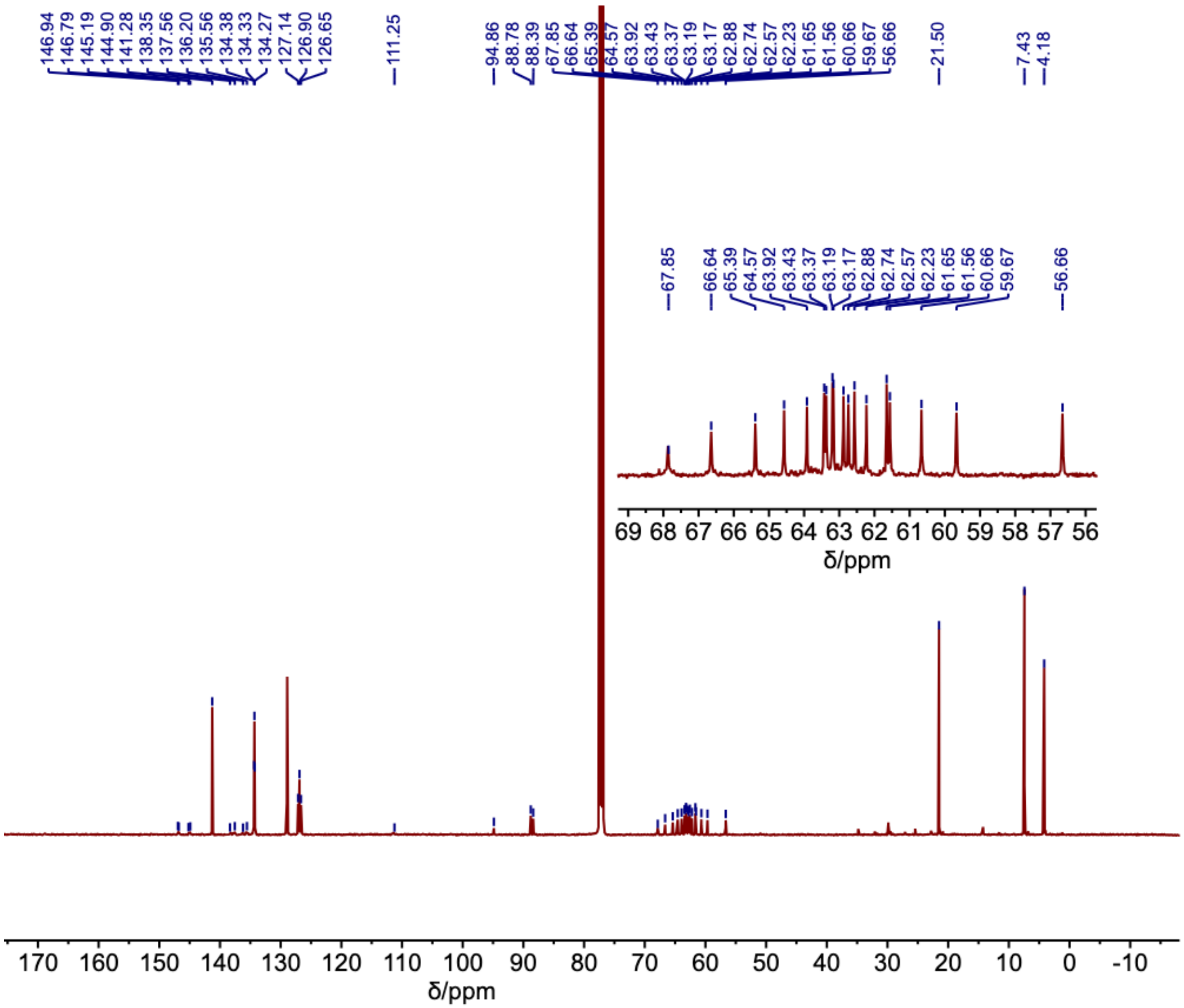

Figure s10. $^{13}C\{^{1}H\}$ NMR spectrum of **PtC$_{22}$Si** ($CDCl_3$, 126 MHz).

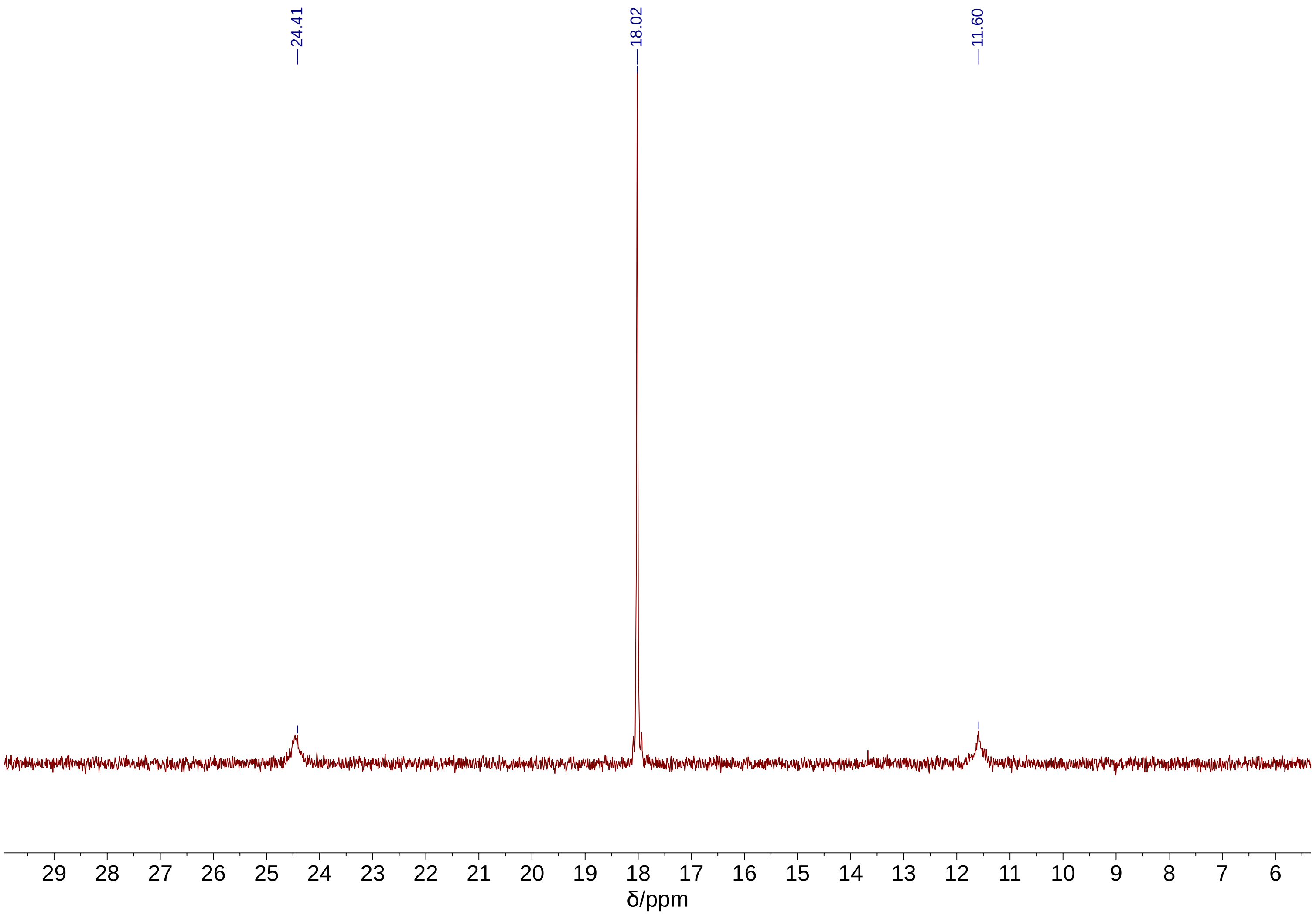

Figure s11. $^{31}P\{^1H\}$ NMR spectrum of **PtC$_{22}$Si** ($CDCl_3$, 202 MHz).

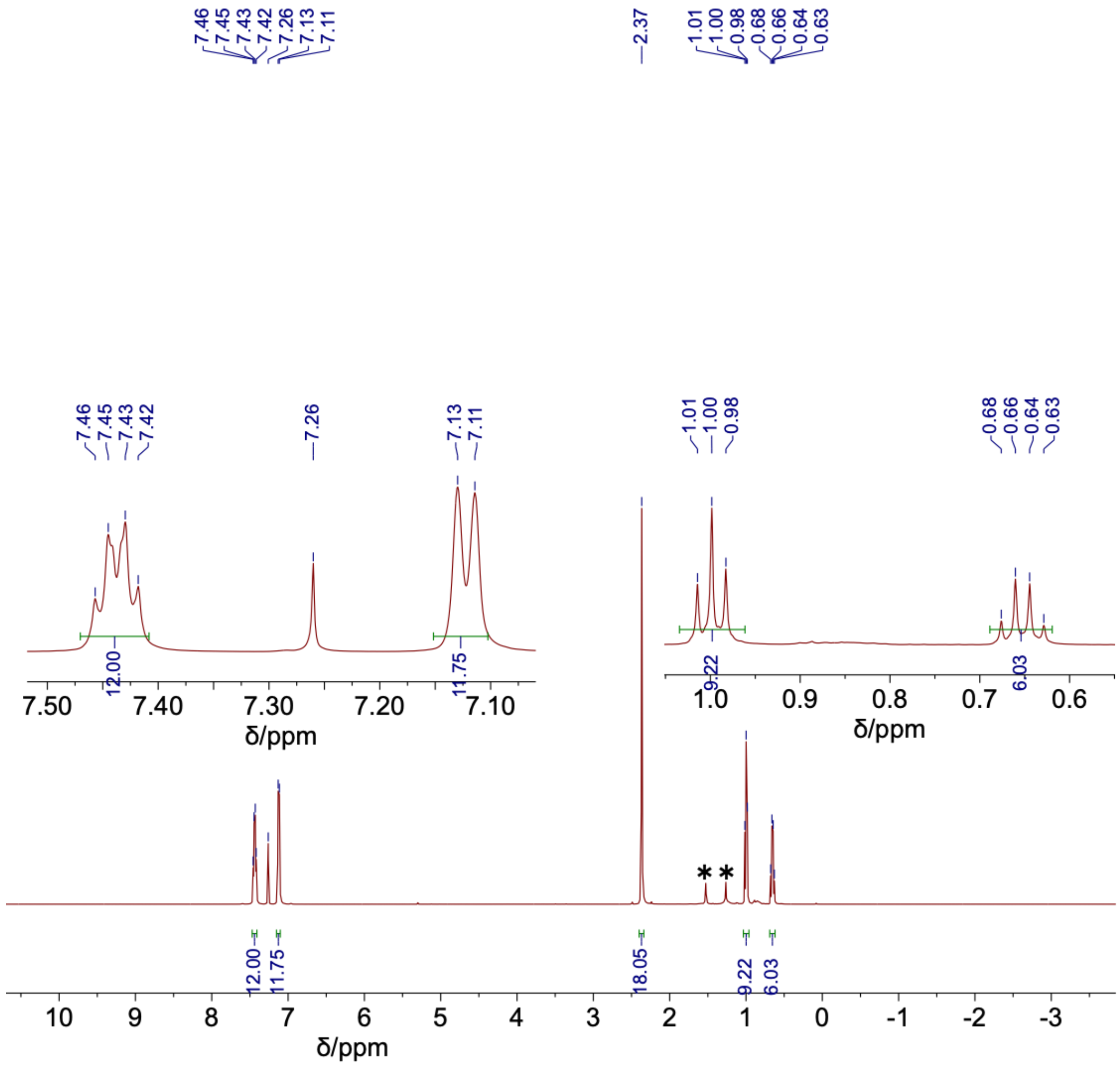

Figure s12. ^{1}H NMR spectrum of **$PtC_{24}Si$** ($CDCl_3$, 500 MHz). * denotes solvent-based impurities.

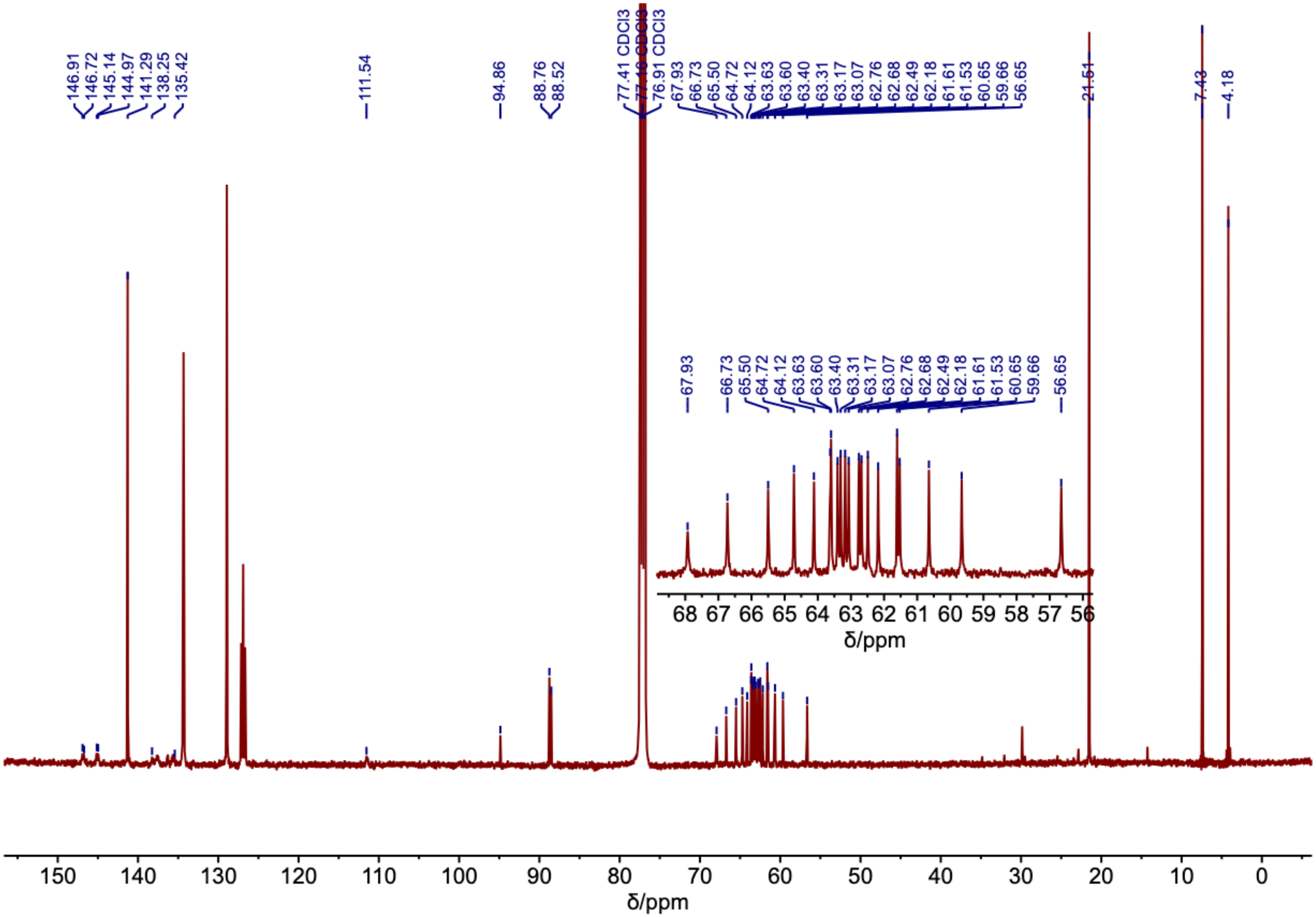

Figure s13. $^{13}C\{^{1}H\}$ NMR spectrum of $\mathbf{PtC_{24}Si}$ ($CDCl_3$, 126 MHz).

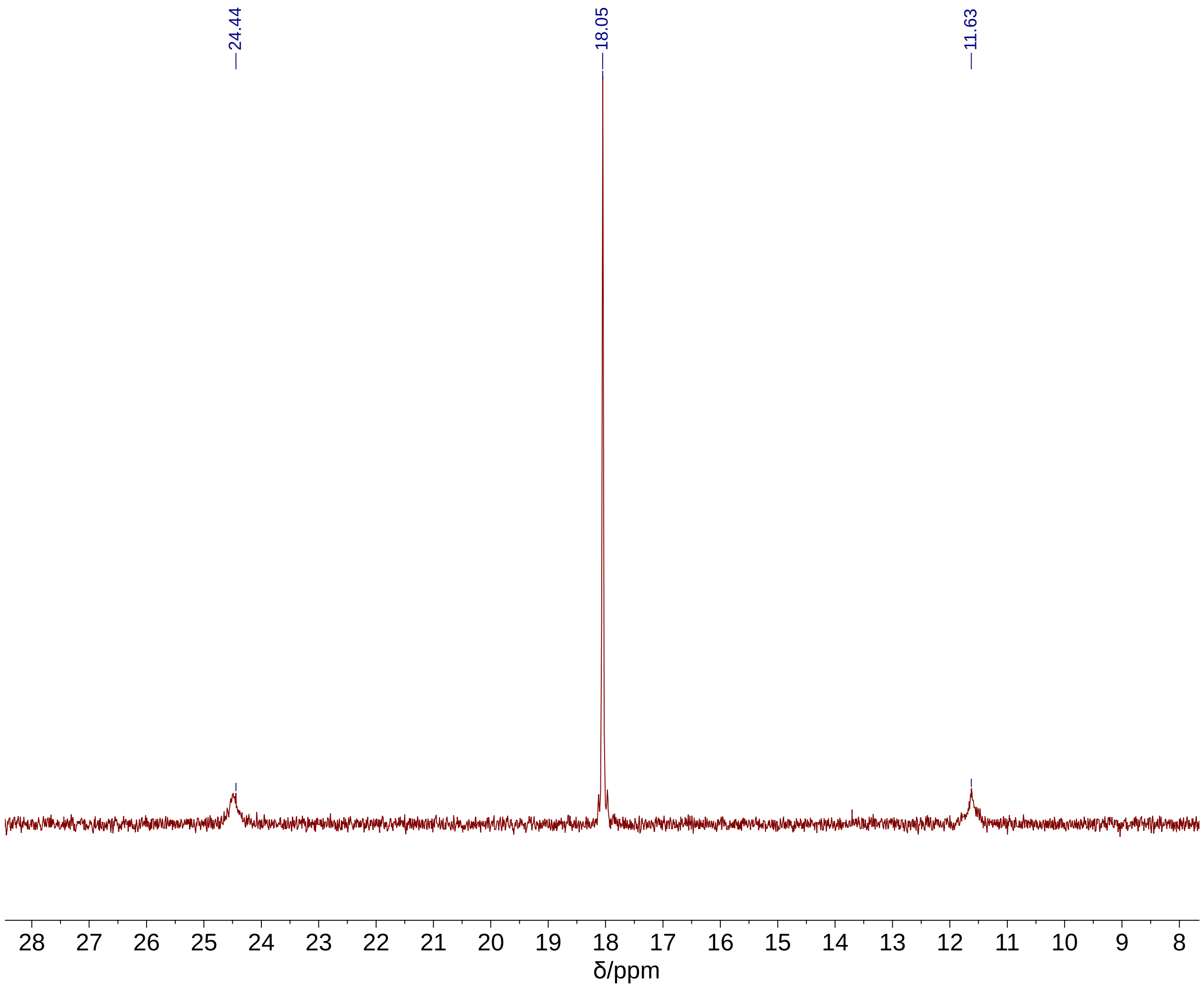

Figure s14. $^{31}P\{^{1}H\}$ NMR spectrum of **$PtC_{24}Si$** ($CDCl_3$, 202 MHz).

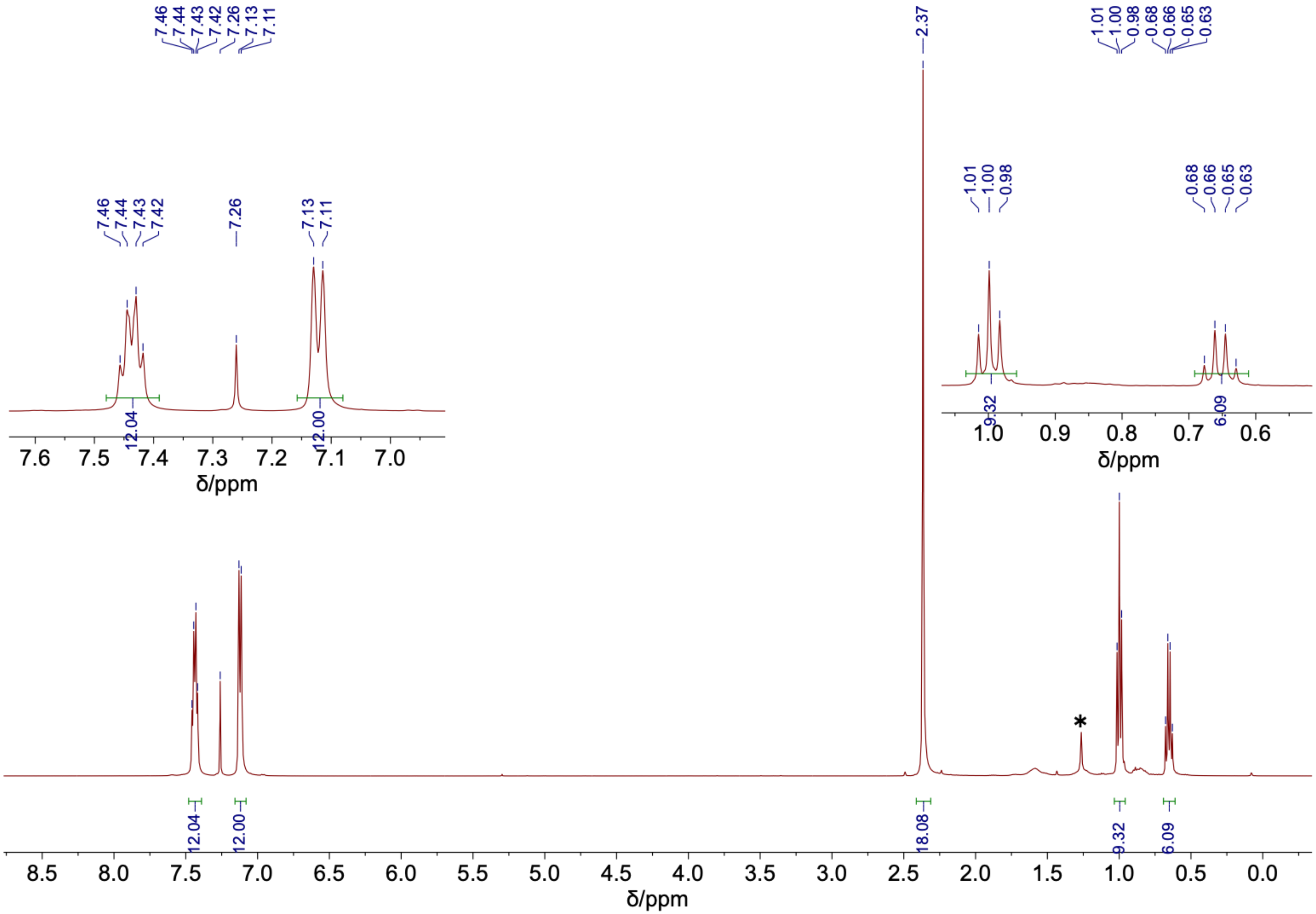

Figure s15. ^{1}H NMR spectrum of **$PtC_{26}Si$** ($CDCl_3$, 500 MHz). * denotes solvent-based impurities.

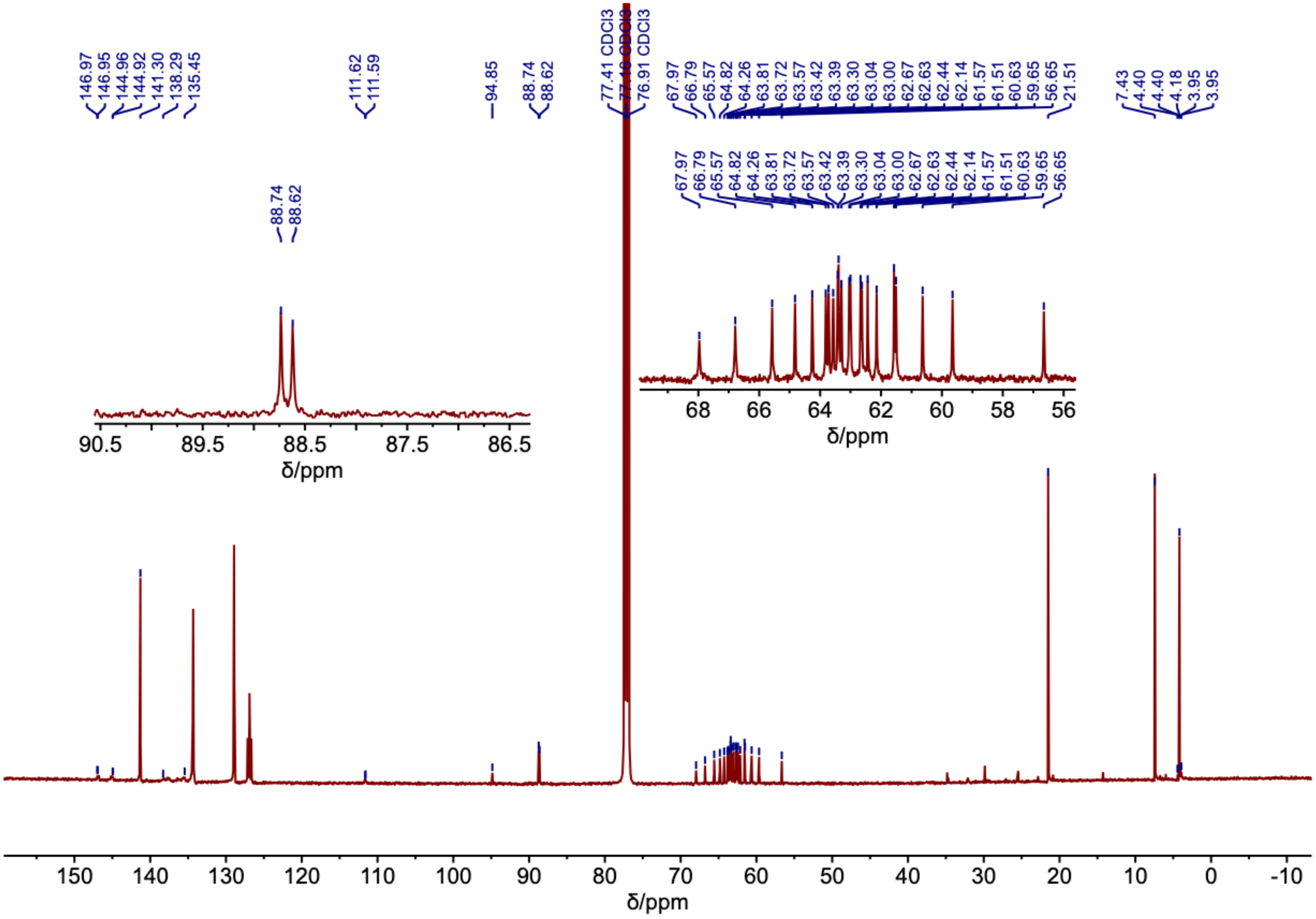

Figure s16. $^{13}C\{^{1}H\}$ NMR spectrum of **$PtC_{26}Si$** ($CDCl_3$, 126 MHz).

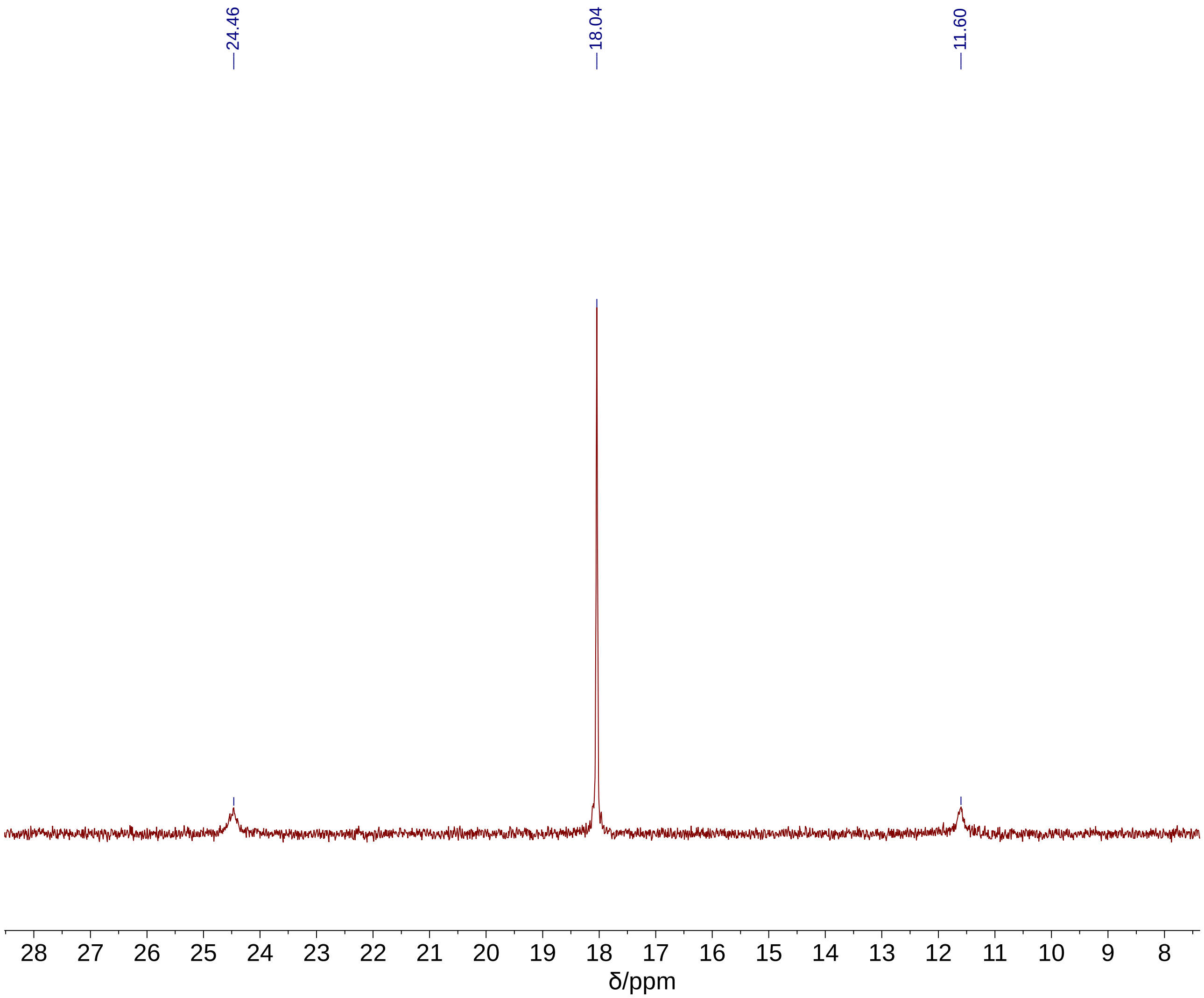

Figure s17. $^{31}P\{^1H\}$ NMR spectrum of **$PtC_{26}Si$** ($CDCl_3$, 202 MHz).

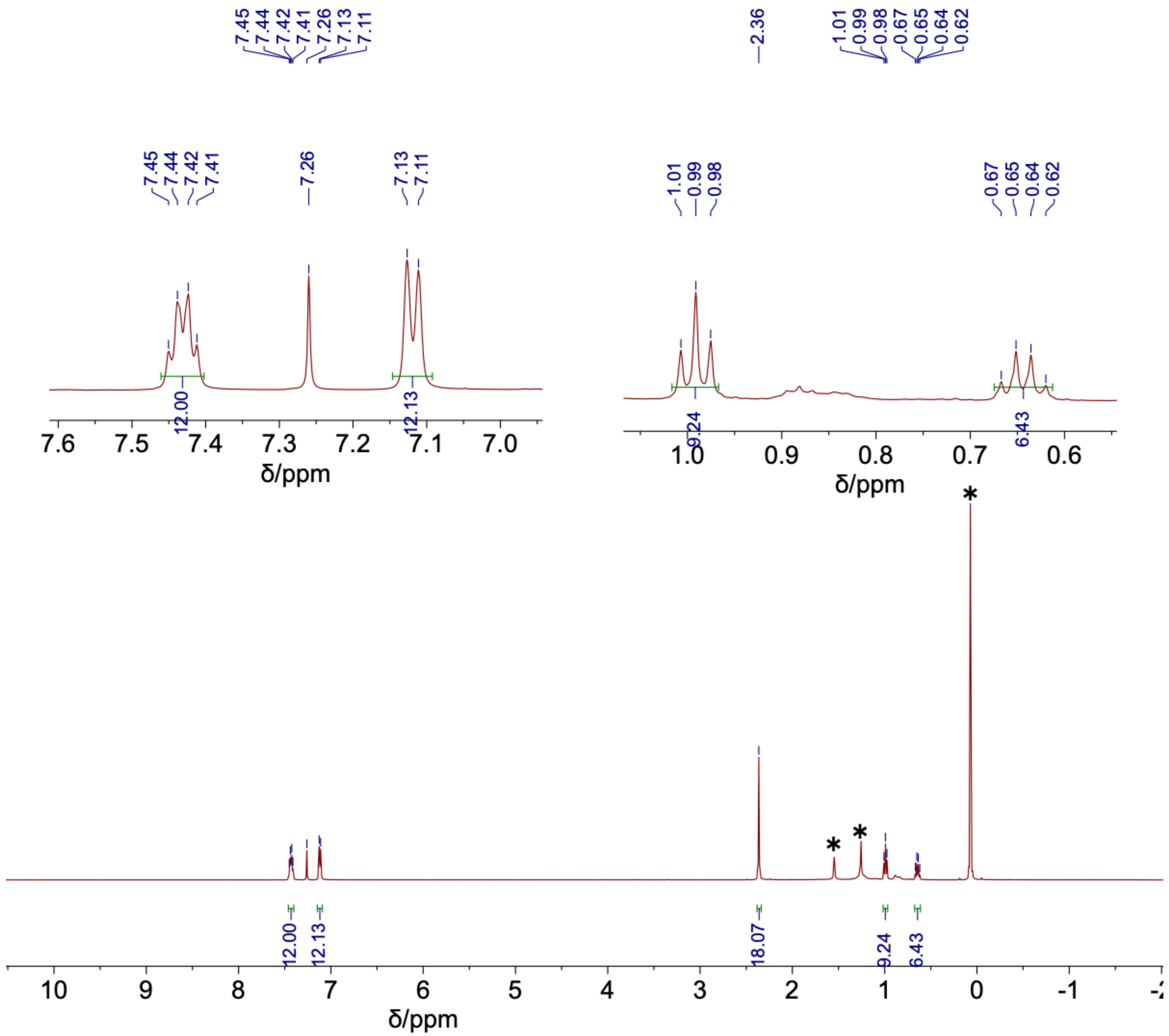

Figure s18. ^{1}H NMR spectrum of **$PtC_{28}Si$** ($CDCl_3$, 500 MHz). * denotes solvent-based impurities.

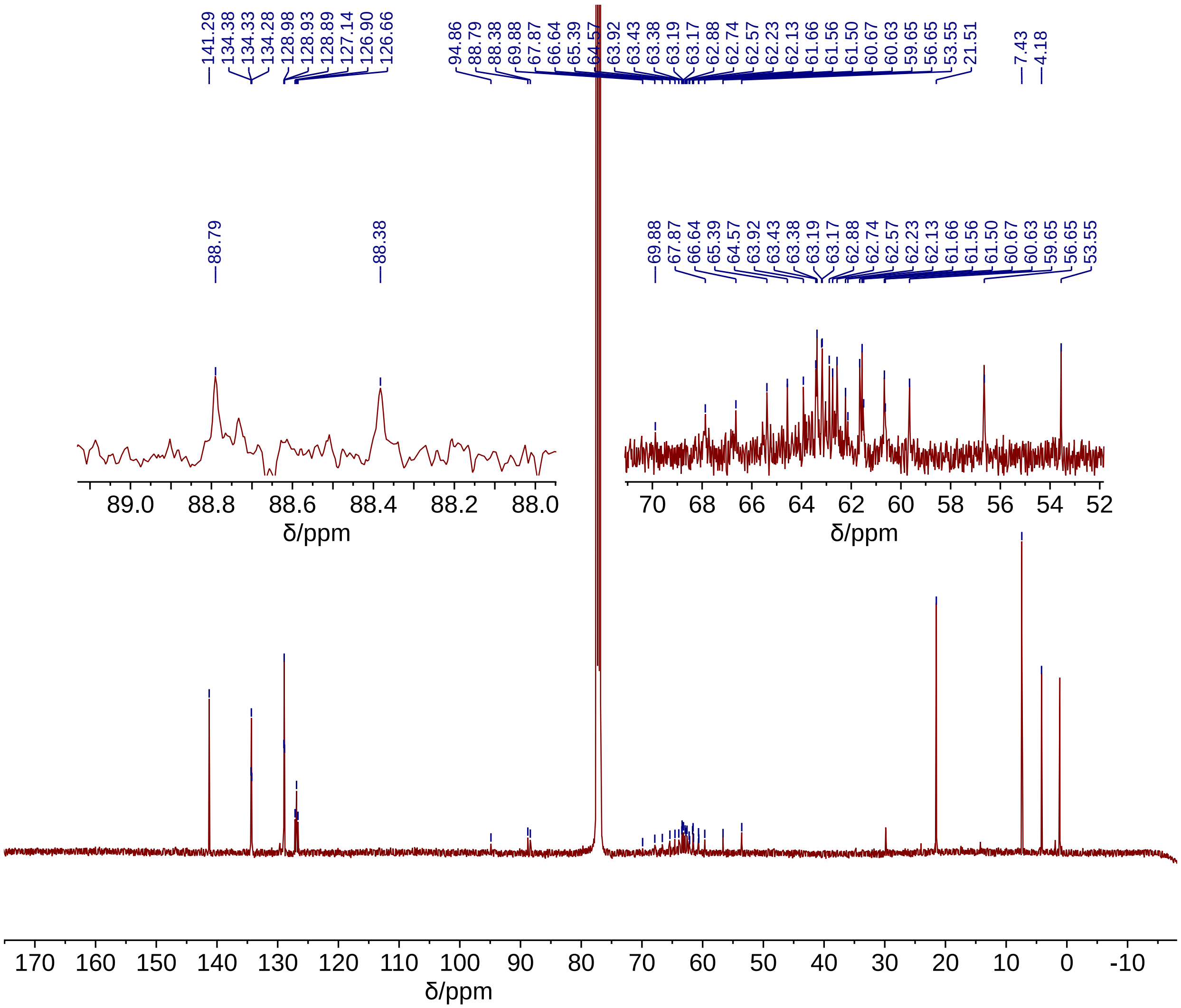

Figure s19. $^{13}C\{^1H\}$ NMR spectrum of $\mathbf{PtC_{28}Si}$ ($CDCl_3$, 126 MHz).

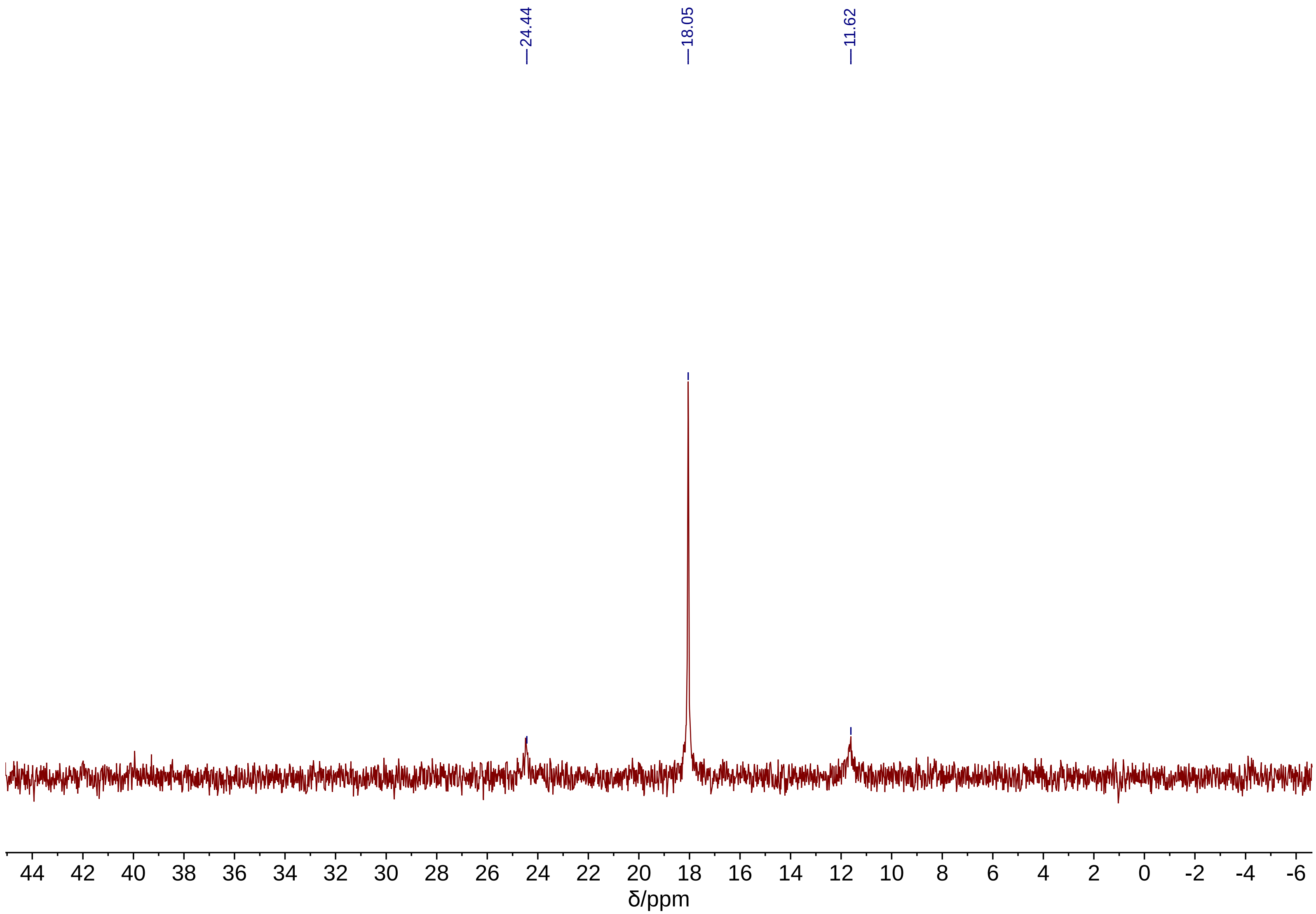

Figure s20. $^{31}P\{^{1}H\}$ NMR spectrum of **$PtC_{28}Si$** ($CDCl_3$, 202 MHz).

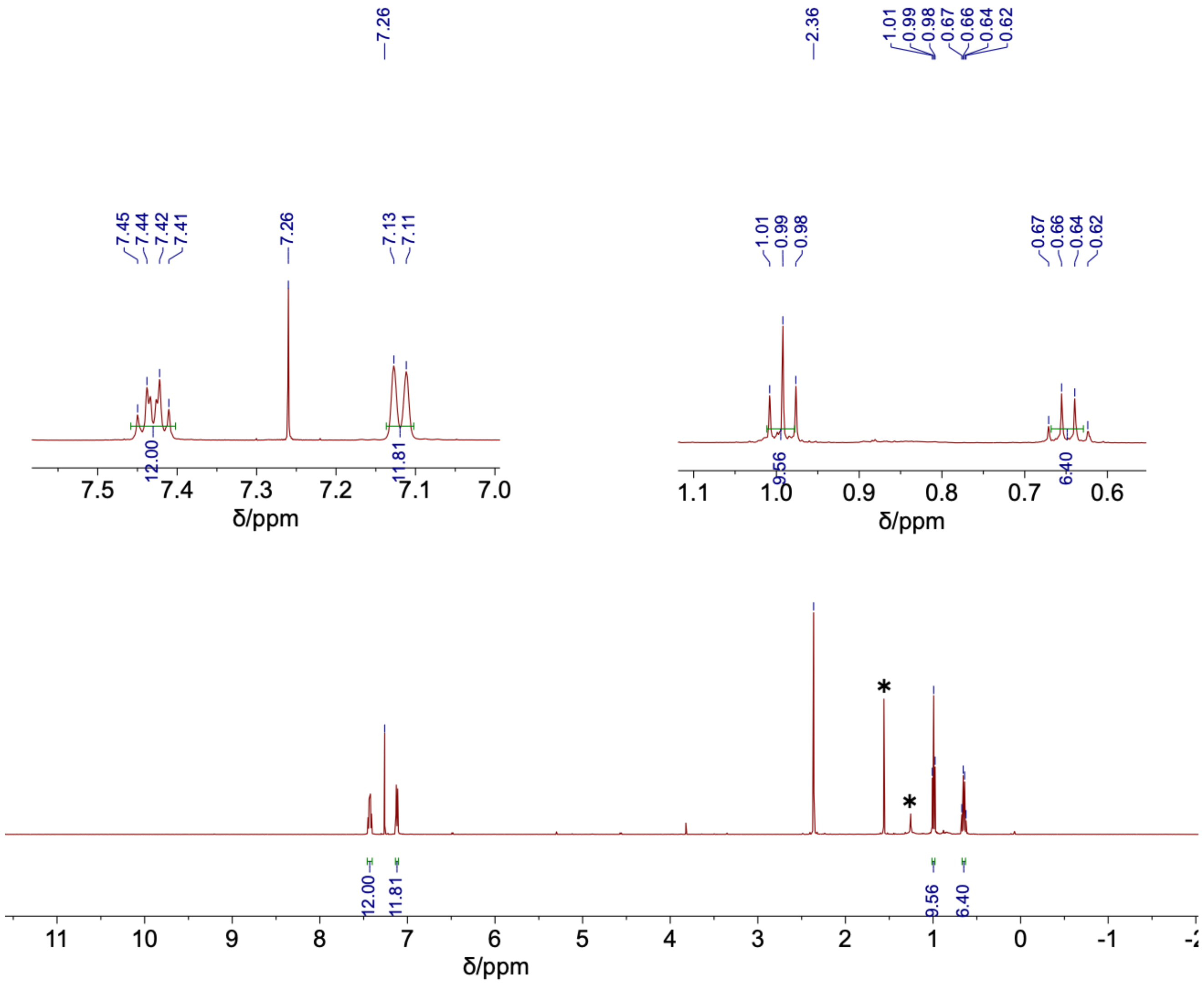

Figure s21. ^{1}H NMR spectrum of **$PtC_{32}Si$** ($CDCl_3$, 500 MHz). * denotes solvent-based impurities.

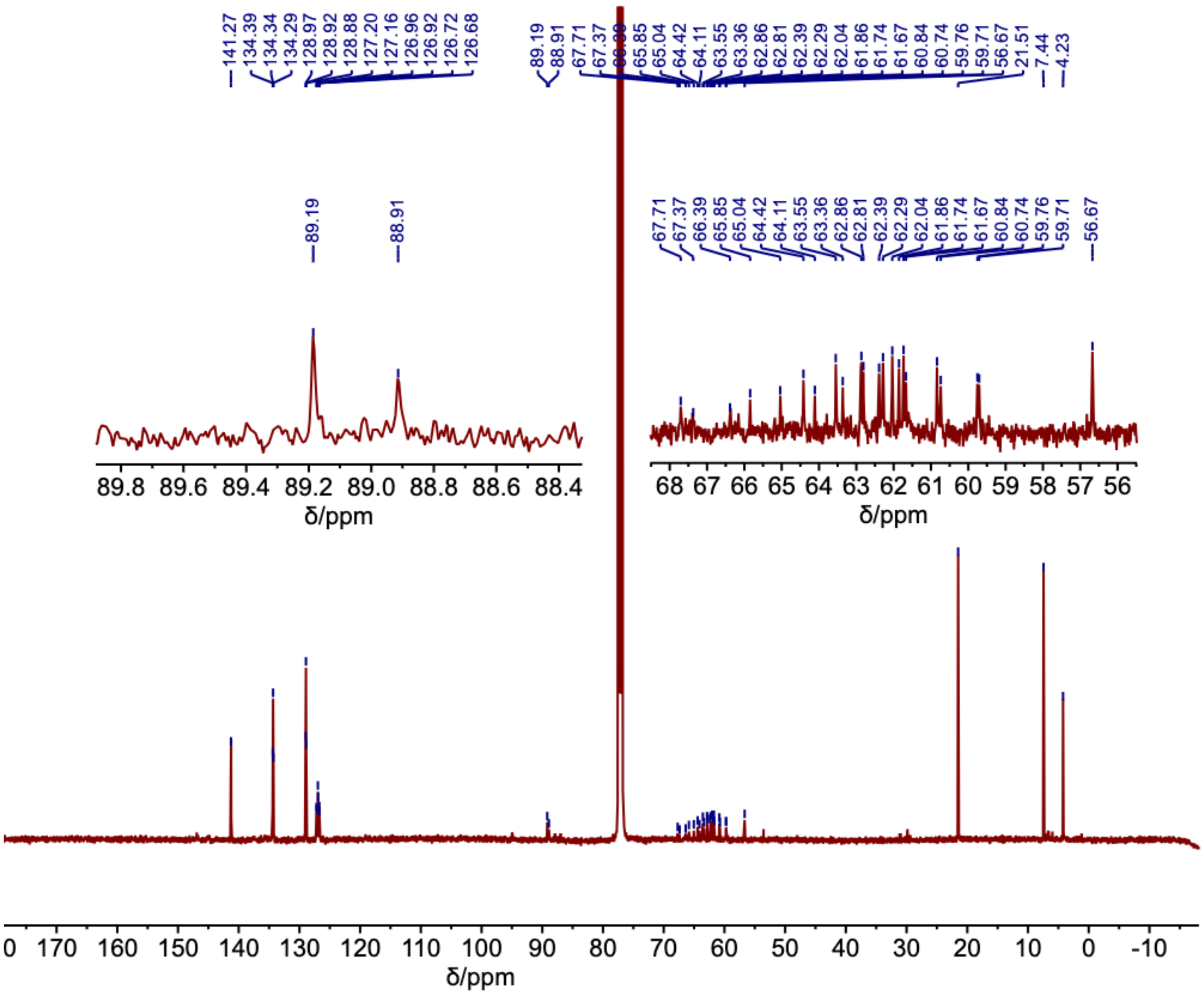

Figure s22. $^{13}C\{^{1}H\}$ NMR spectrum of $\mathbf{PtC_{32}Si}$ ($CDCl_3$, 126 MHz).

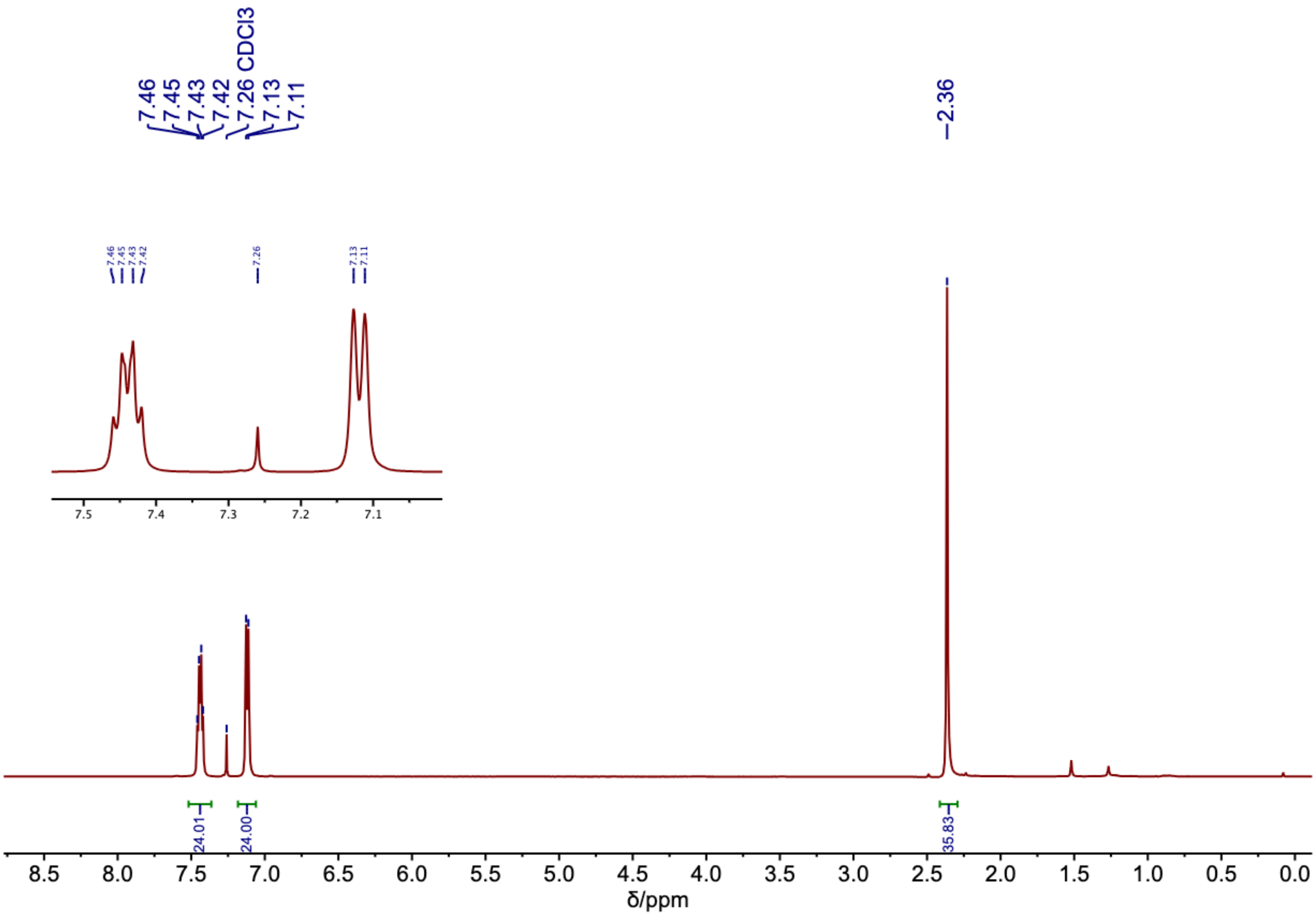

Figure s23. ^{1}H NMR spectrum of **PtC$_{20}$Pt** (CDCl$_3$, 500 MHz).

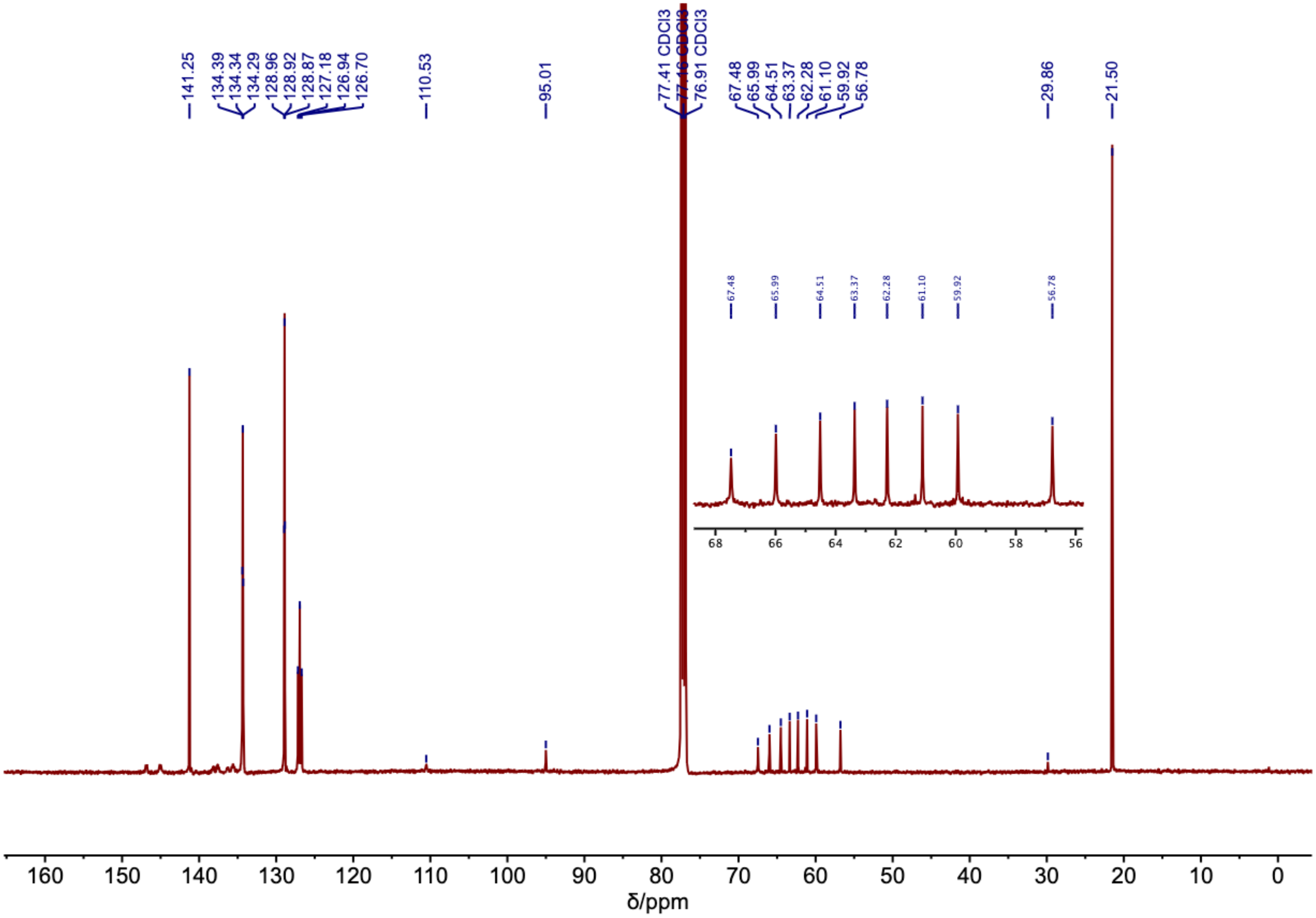

Figure s24. $^{13}C\{^1H\}$ NMR spectrum of $\mathbf{PtC_{20}Pt}$ ($CDCl_3$, 126 MHz).

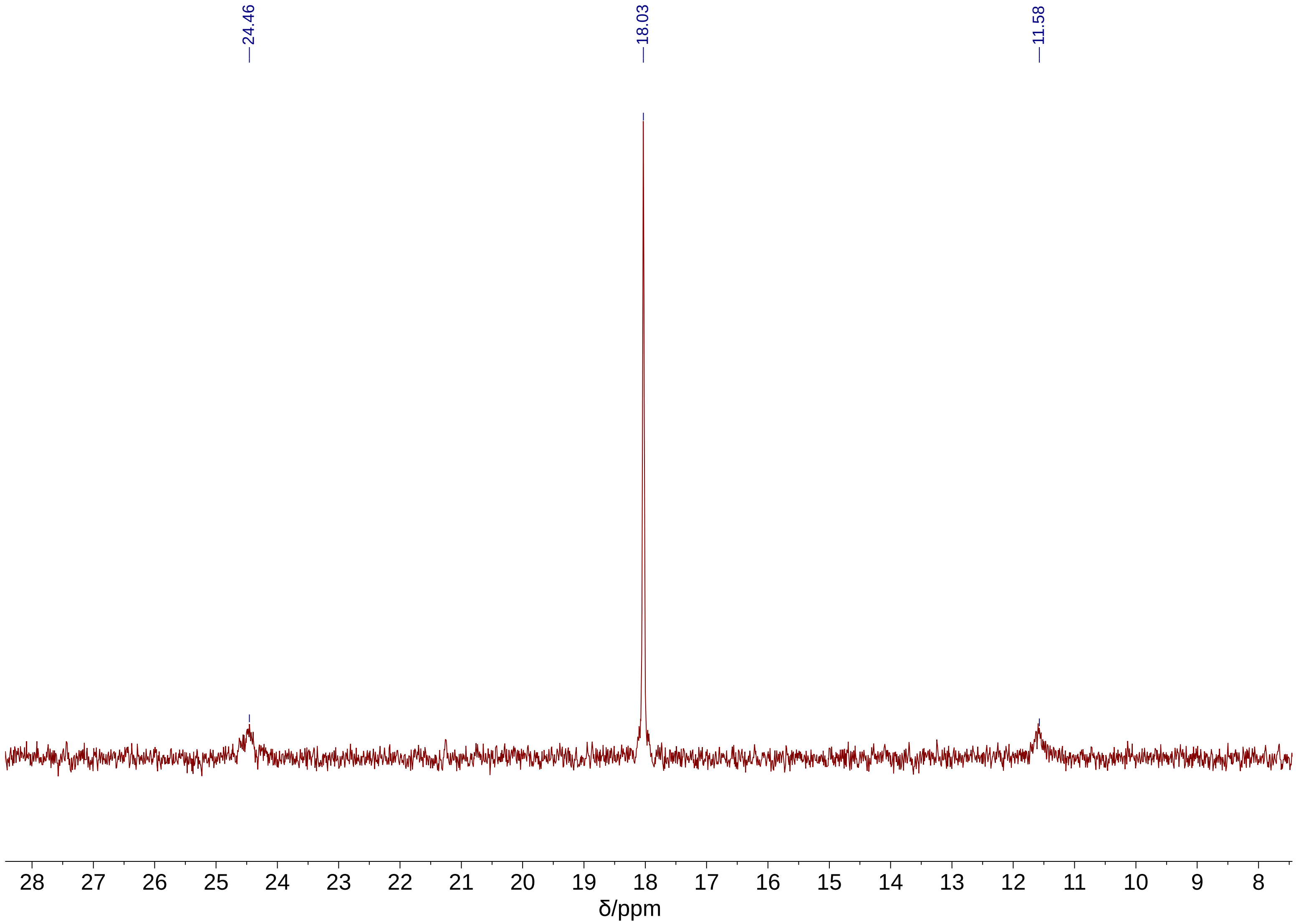

Figure s25. $^{31}P\{^1H\}$ NMR spectrum of **$PtC_{20}Pt$** ($CDCl_3$, 202 MHz).

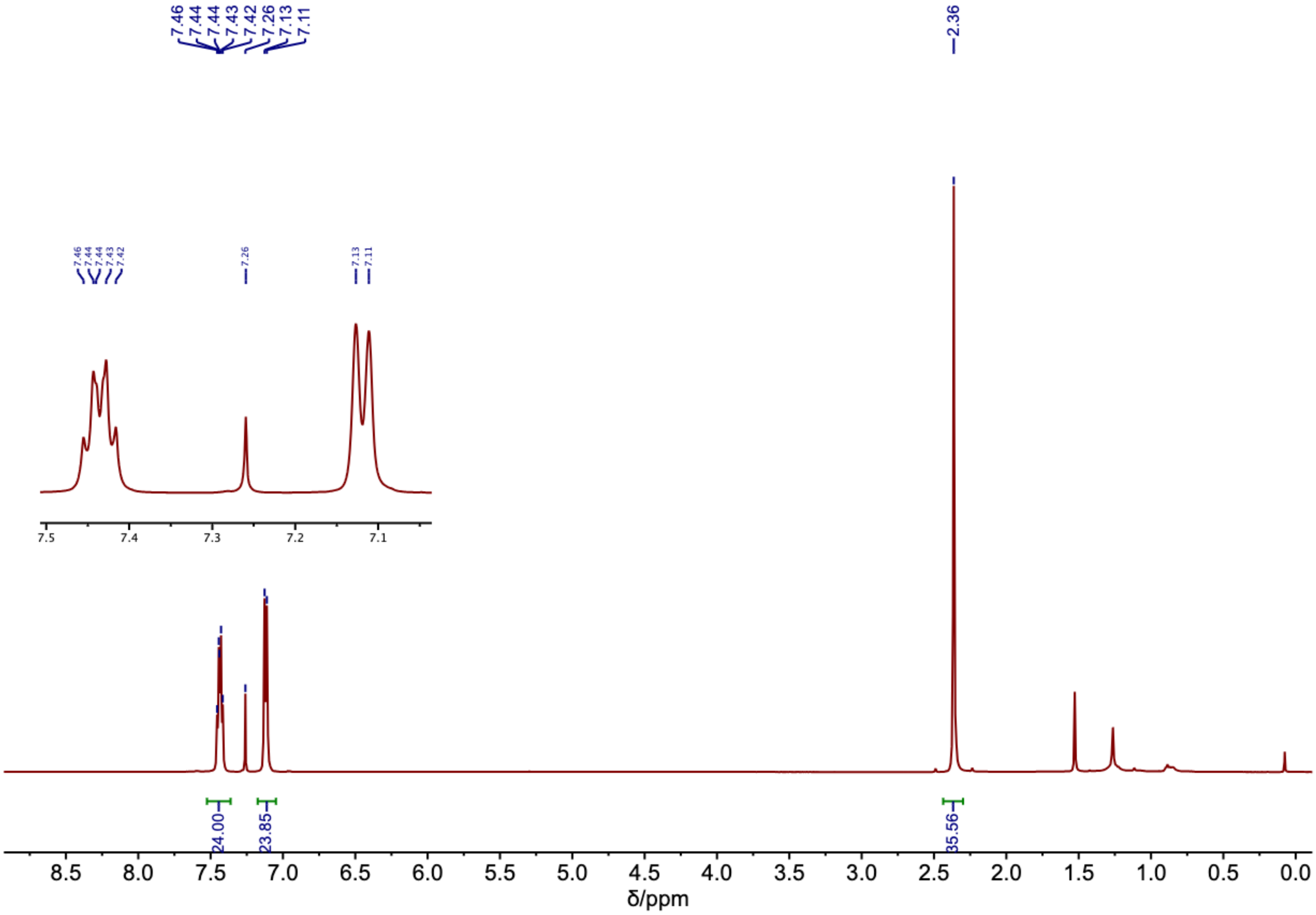

Figure s26. ^{1}H NMR spectrum of **$PtC_{24}Pt$** ($CDCl_3$, 500 MHz).

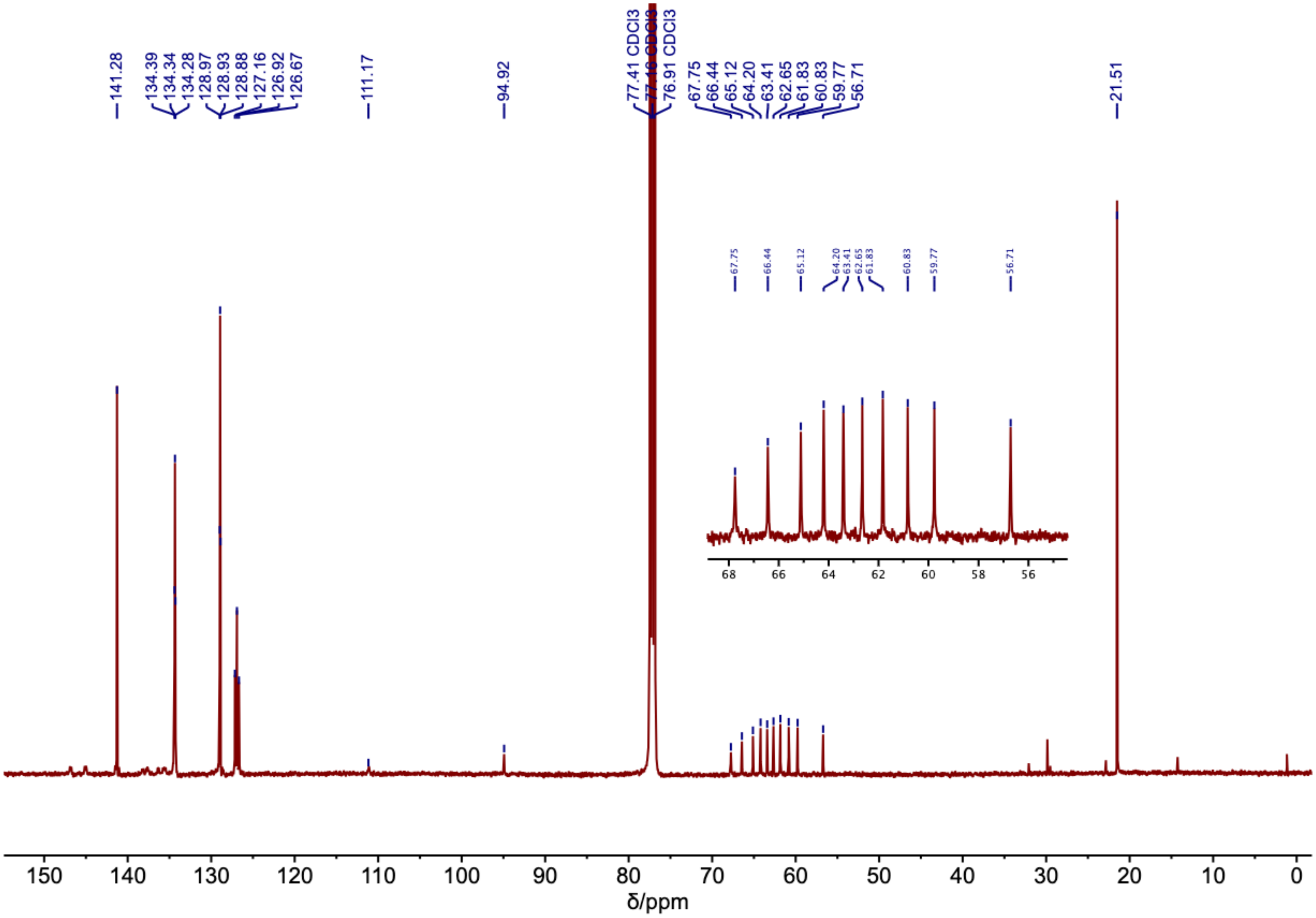

Figure s27. $^{13}C\{^1H\}$ NMR spectrum of **$PtC_{24}Pt$** ($CDCl_3$, 126 MHz).

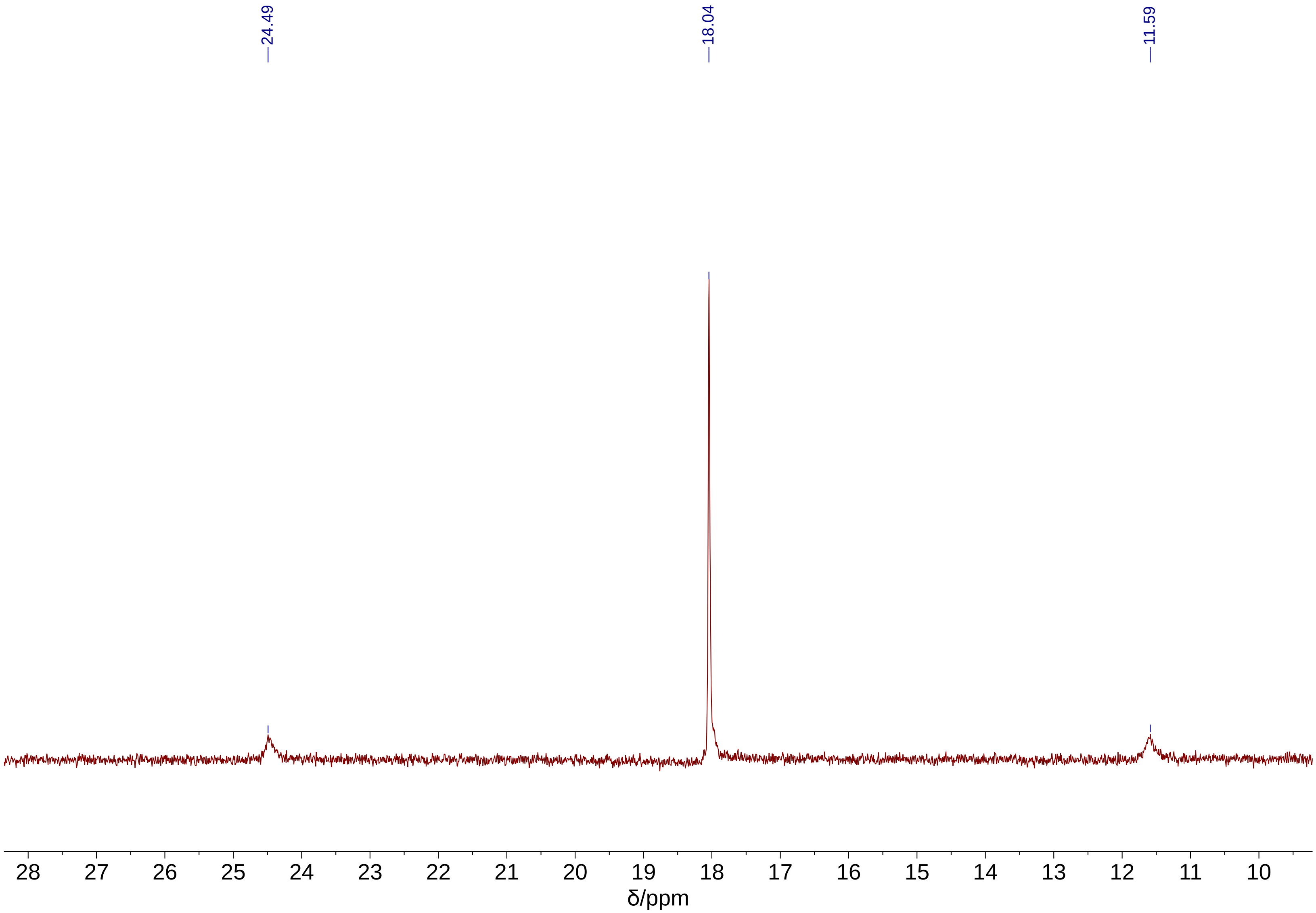

Figure s28. $^{31}P\{^1H\}$ NMR spectrum of **$PtC_{24}Pt$** ($CDCl_3$, 202 MHz).

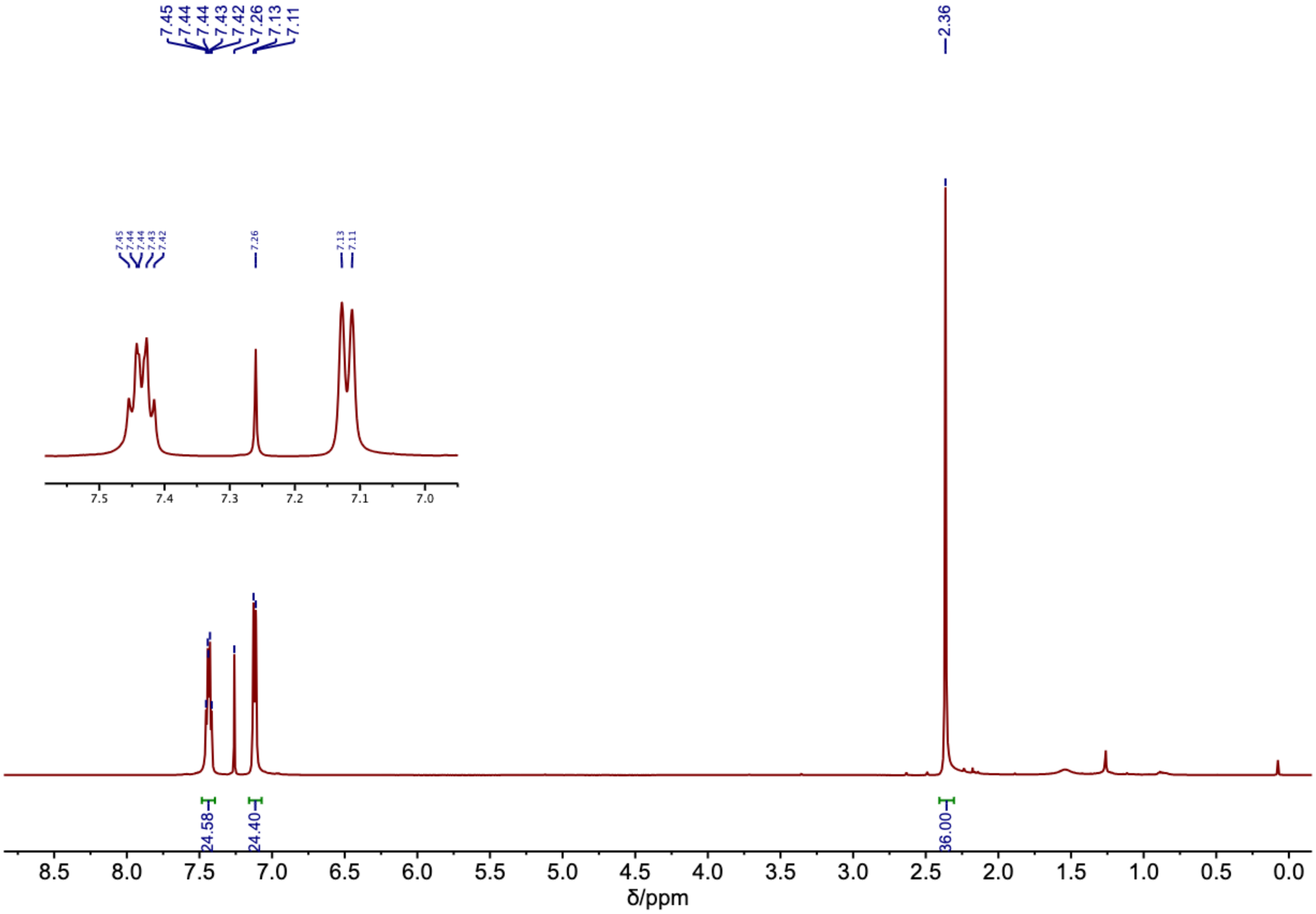

Figure s29. ^{1}H NMR spectrum of **$PtC_{28}Pt$** ($CDCl_3$, 500 MHz).

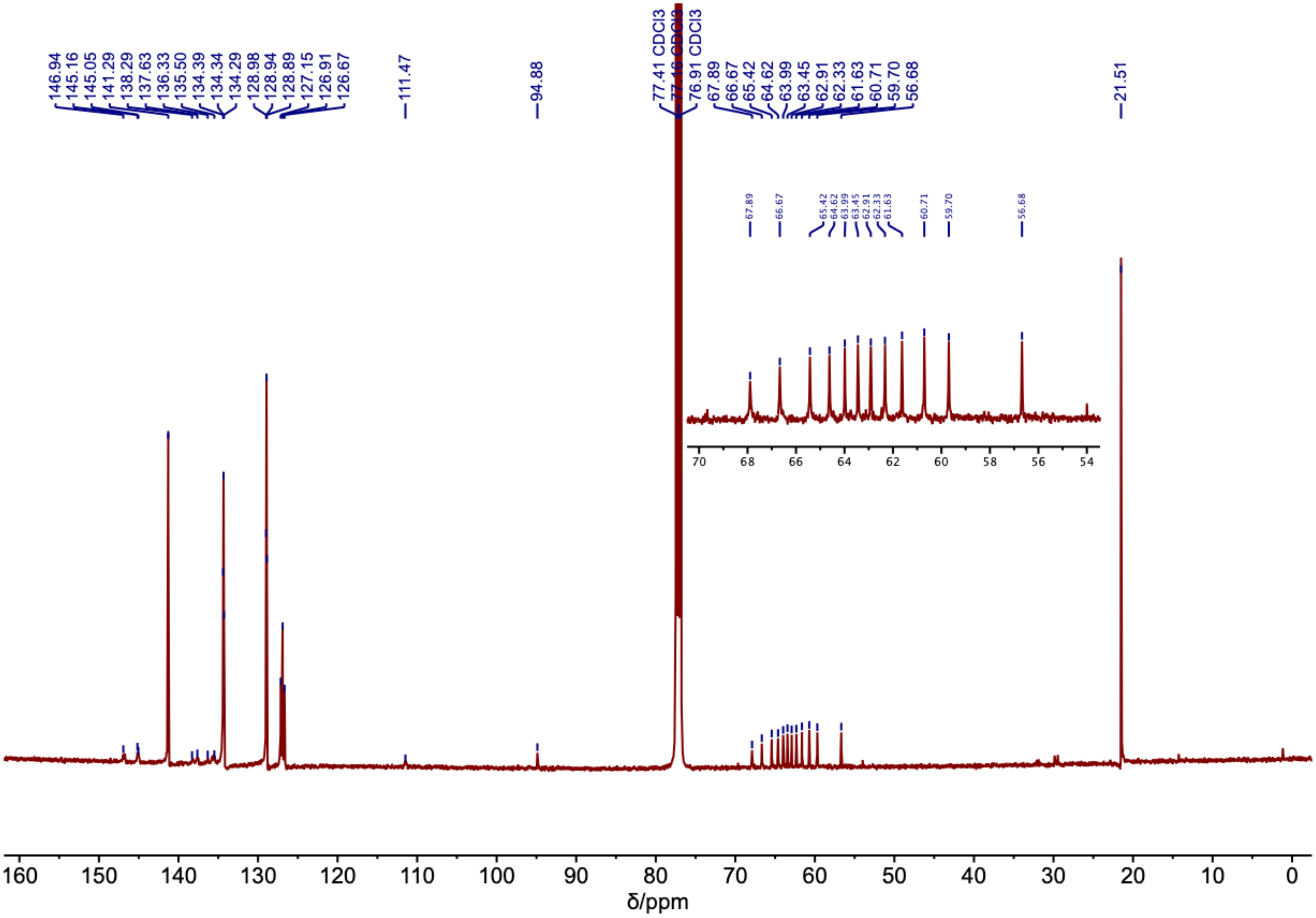

Figure s30. $^{13}C\{^{1}H\}$ NMR spectrum of **$PtC_{28}Pt$** ($CDCl_3$, 126 MHz).

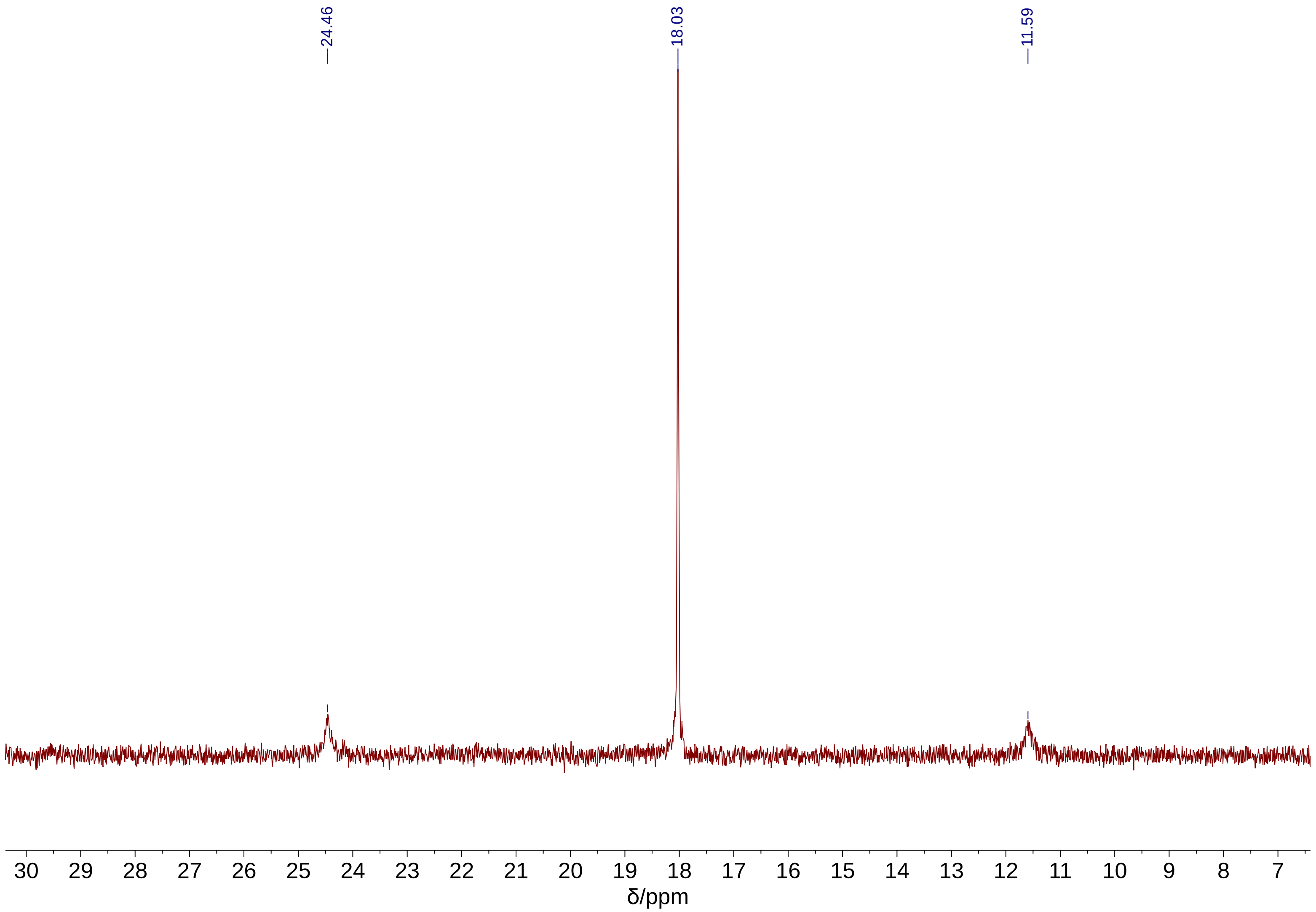

Figure s31. $^{31}P\{^1H\}$ NMR spectrum of **$PtC_{28}Pt$** ($CDCl_3$, 202 MHz).

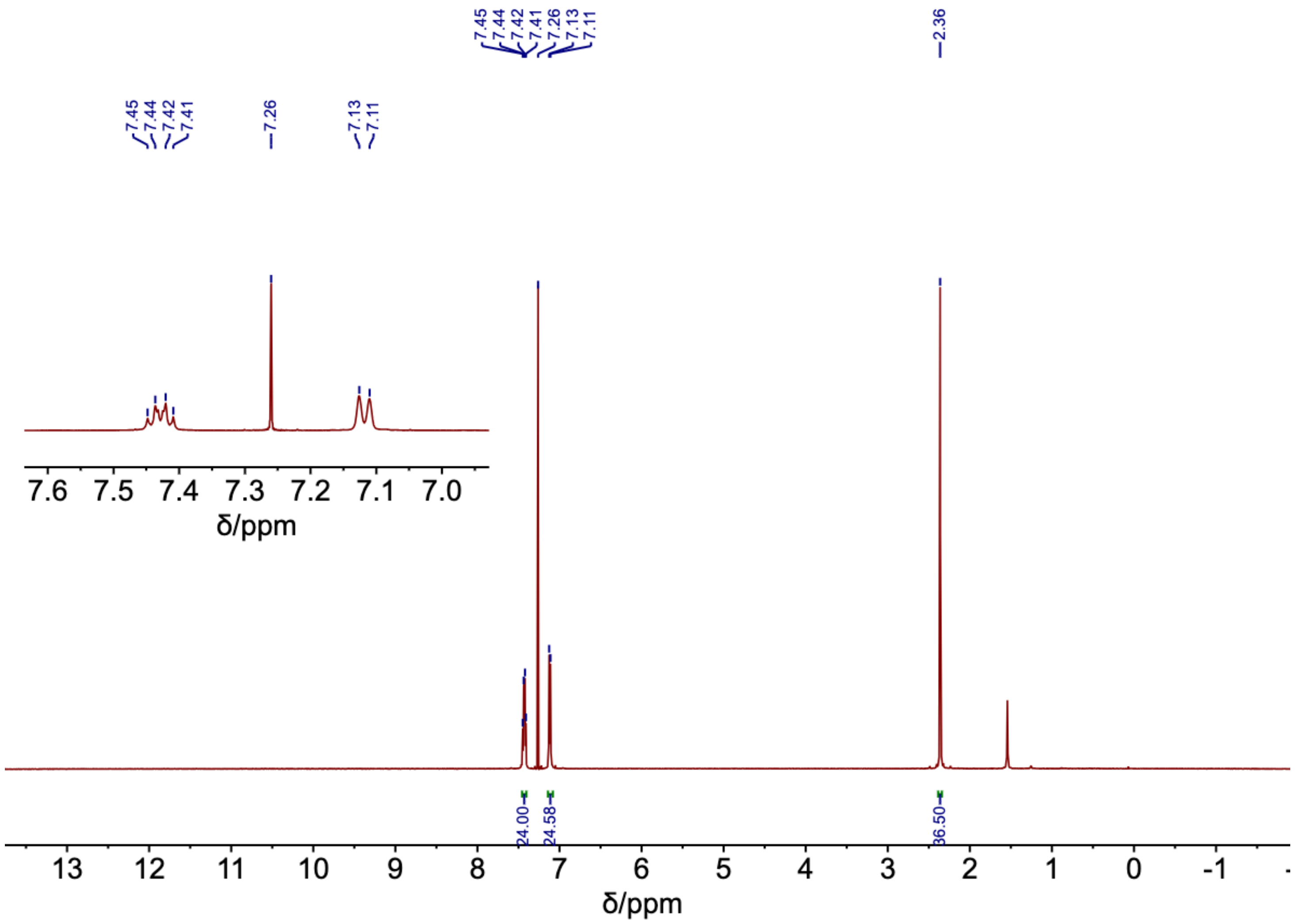

Figure s32. ^{1}H NMR spectrum of **$PtC_{32}Pt$** ($CDCl_3$, 500 MHz).

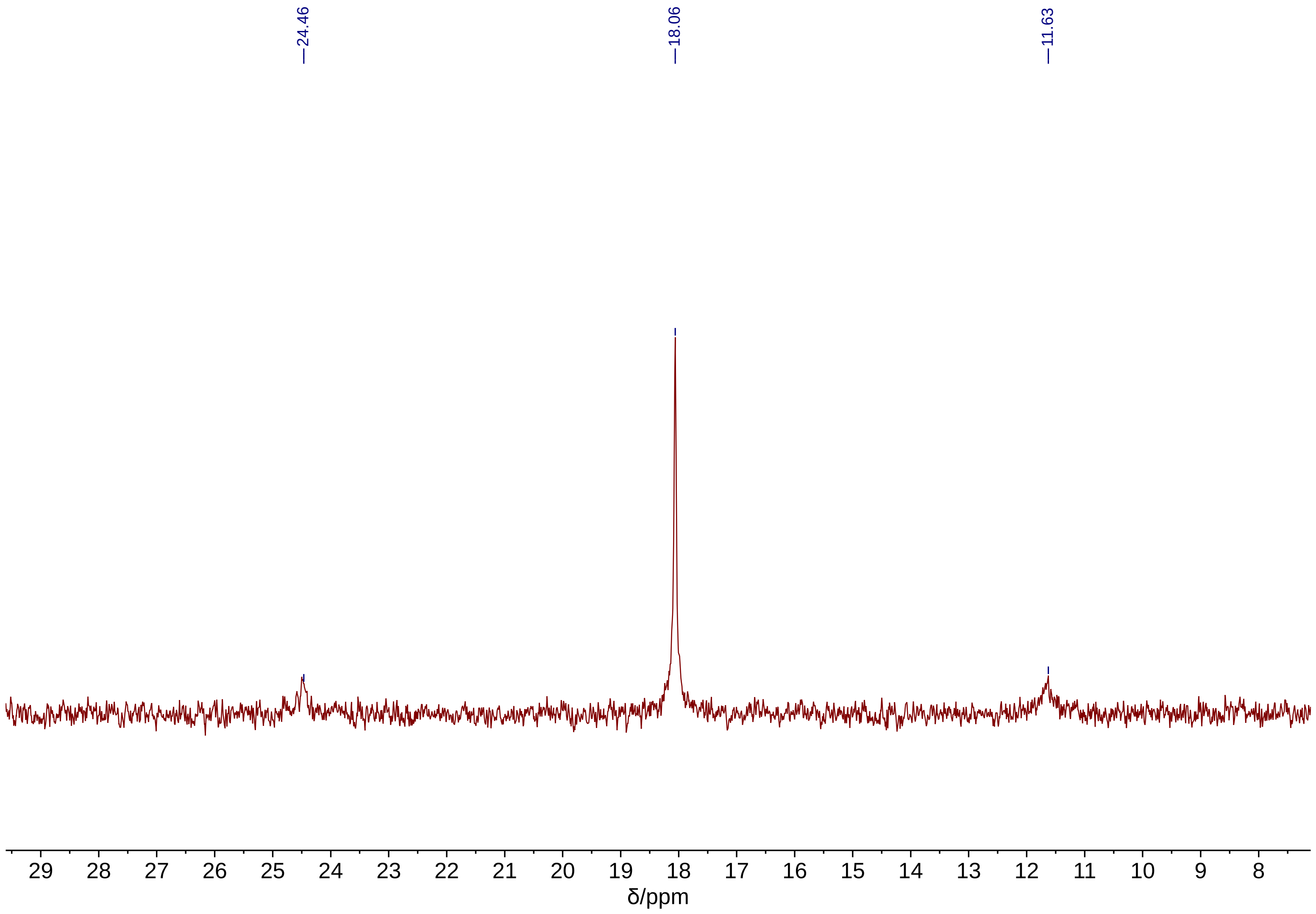

Figure s33. $^{31}P\{^1H\}$ NMR spectrum of **$PtC_{32}Pt$** ($CDCl_3$, 202 MHz).

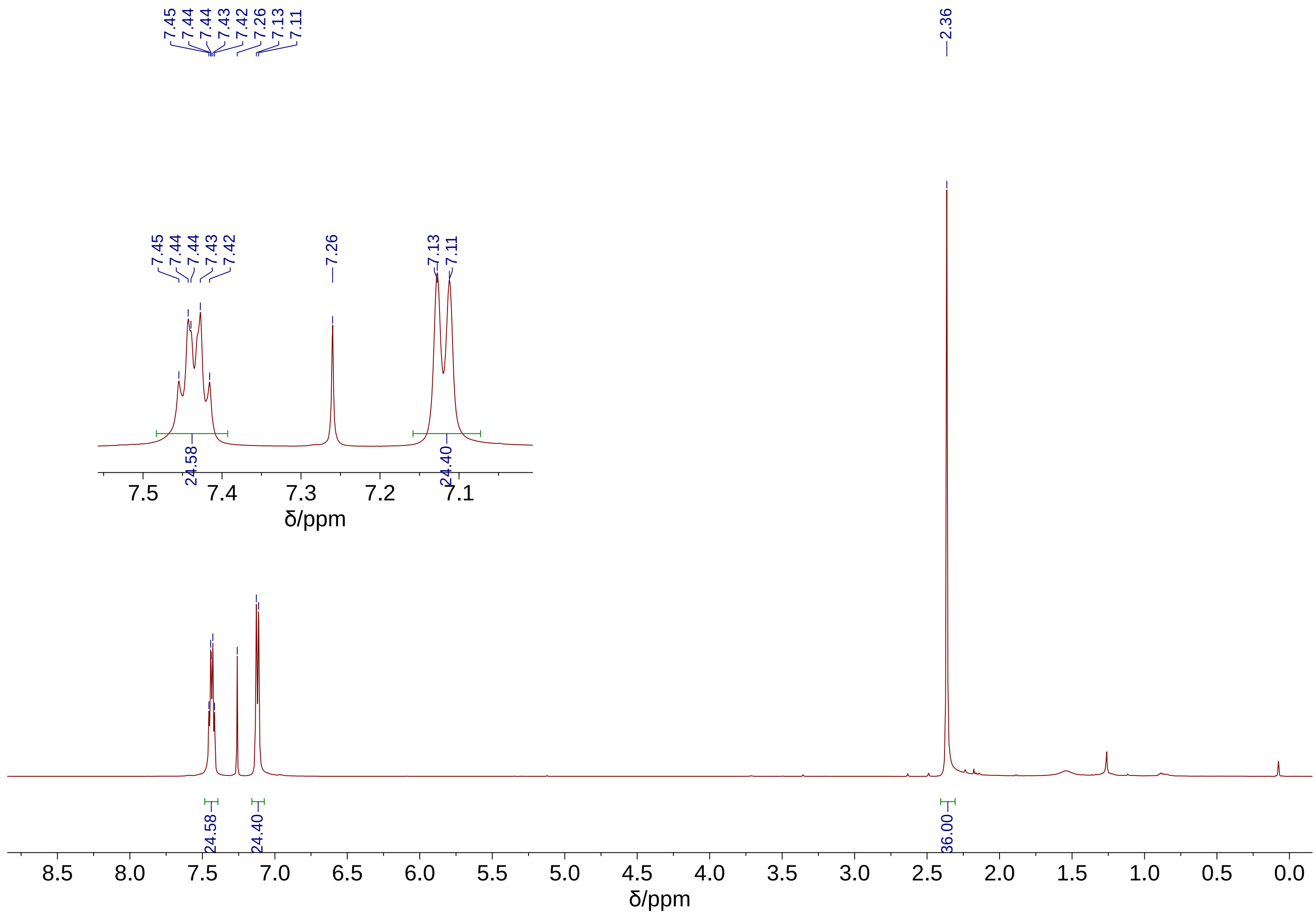

Figure s34. ^{1}H NMR spectrum of **$PtC_{36}Pt$** ($CDCl_3$, 500 MHz).

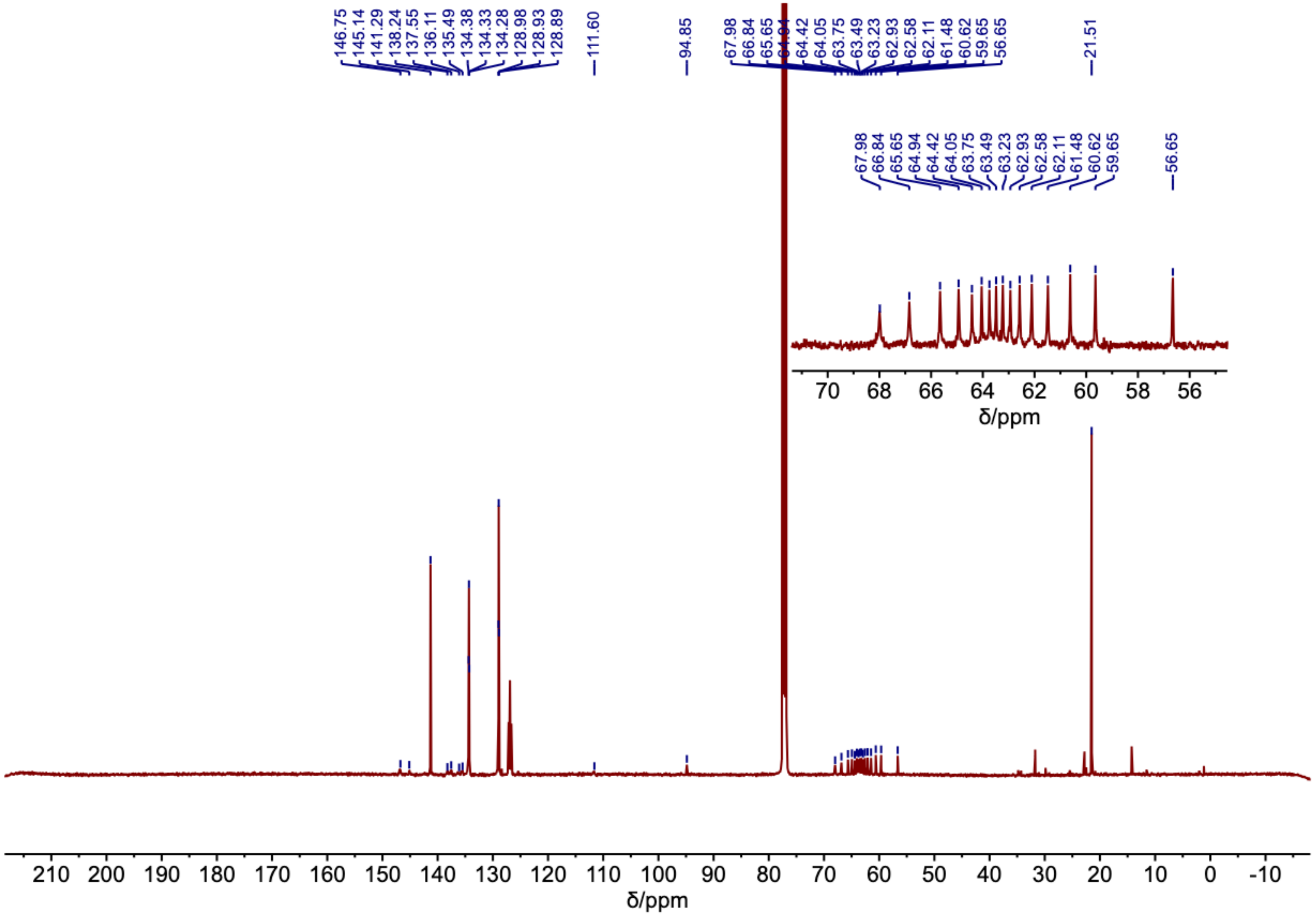

Figure s35. $^{13}C\{^{1}H\}$ NMR spectrum of **$PtC_{36}Pt$** ($CDCl_3$, 126 MHz).

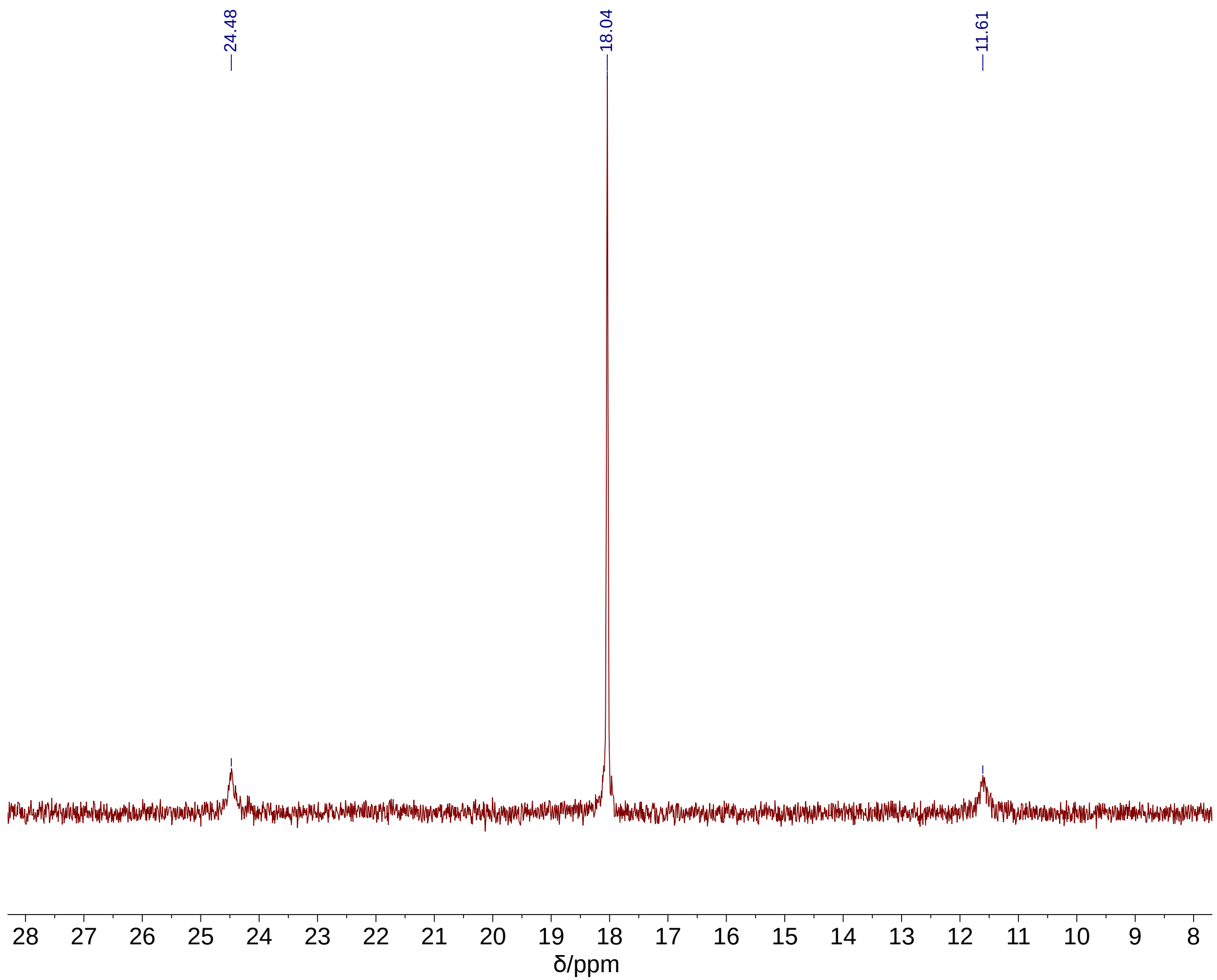

Figure s36. $^{31}P\{^{1}H\}$ NMR spectrum of **$PtC_{36}Pt$** ($CDCl_3$, 202 MHz).

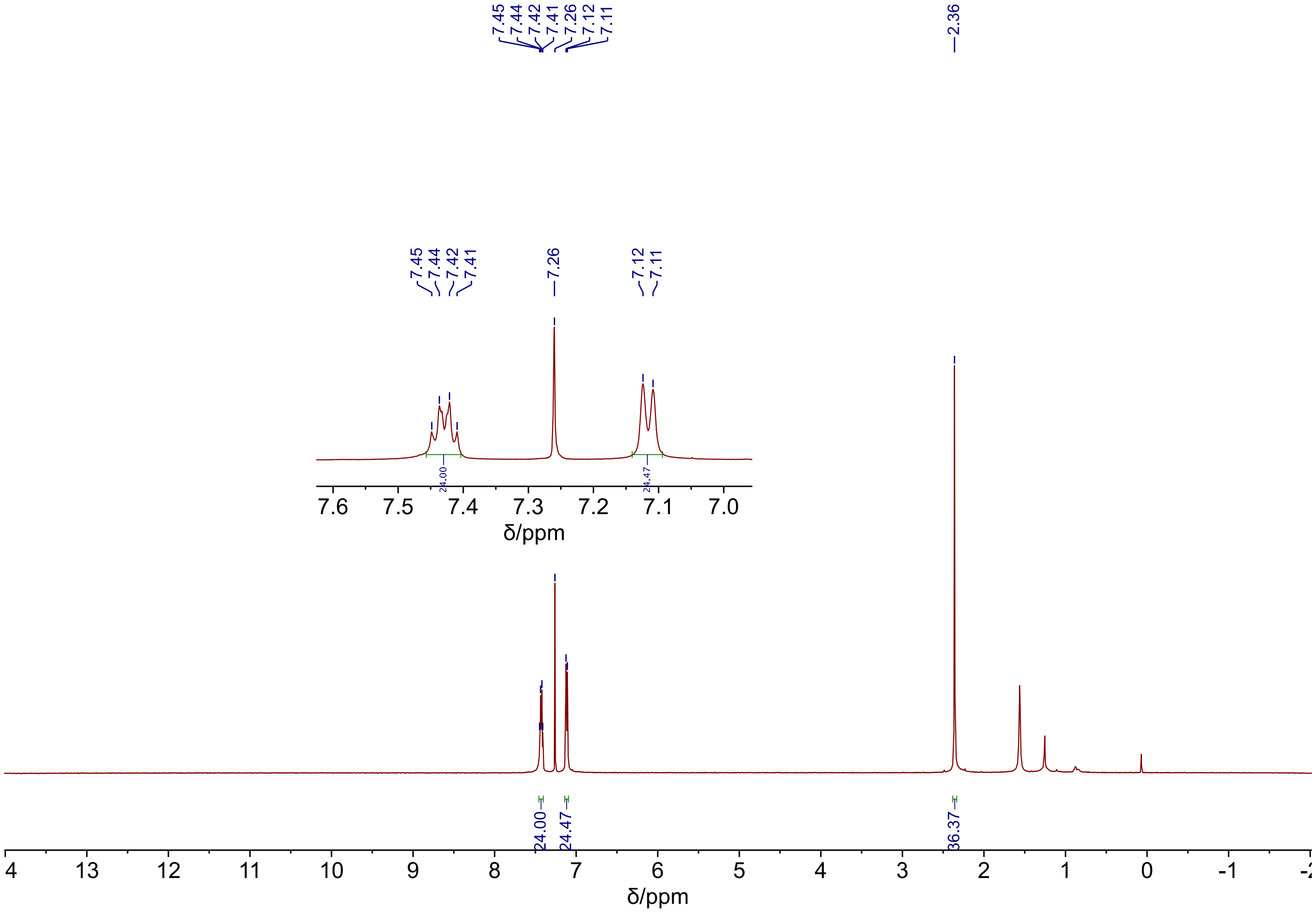

Figure s37. ^{1}H NMR spectrum of **$PtC_{40}Pt$** ($CDCl_3$, 500 MHz).

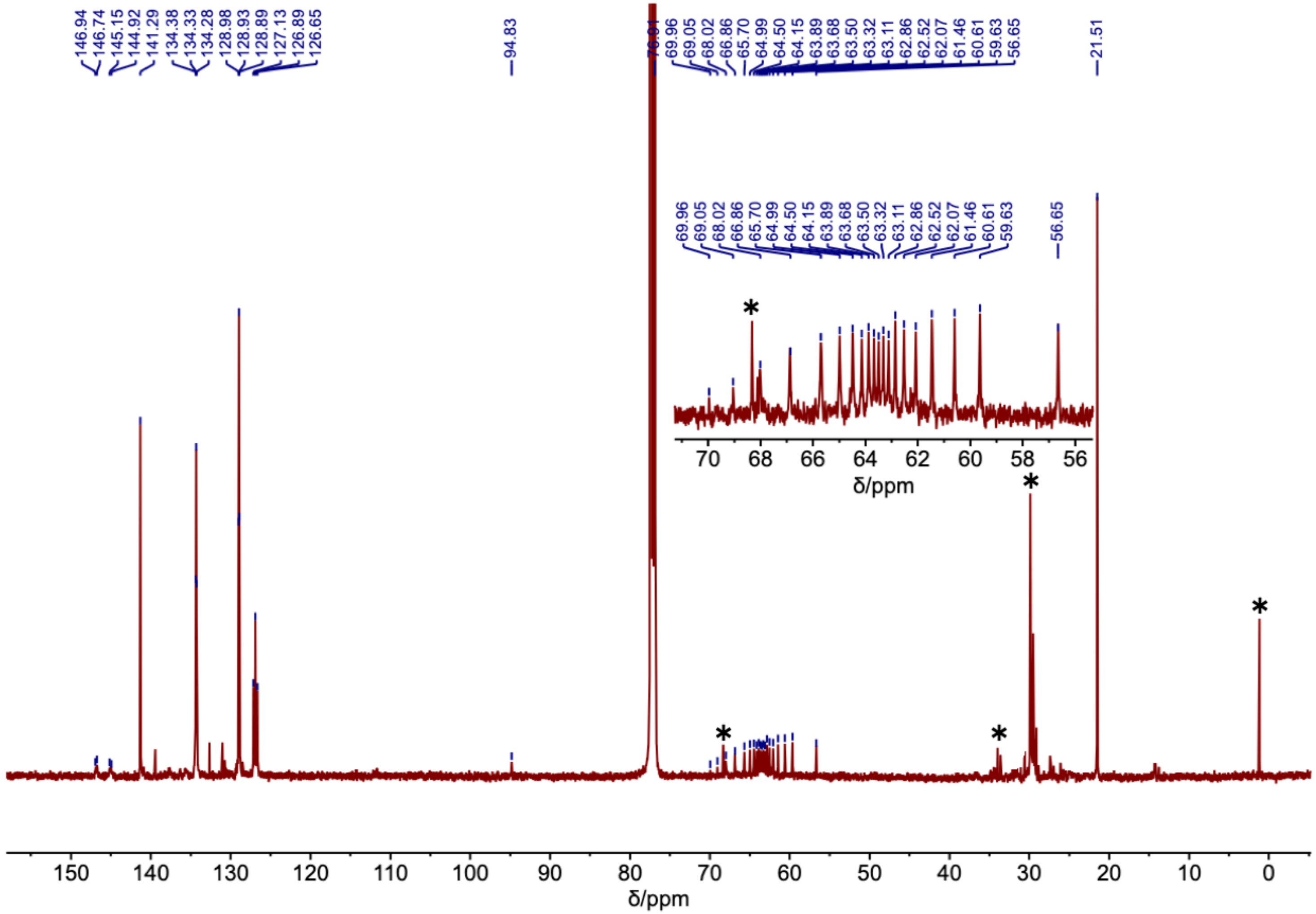

Figure s38. $^{13}C\{^{1}H\}$ NMR spectrum of **$PtC_{40}Pt$** ($CDCl_3$, 126 MHz). * denotes solvent-based impurities.

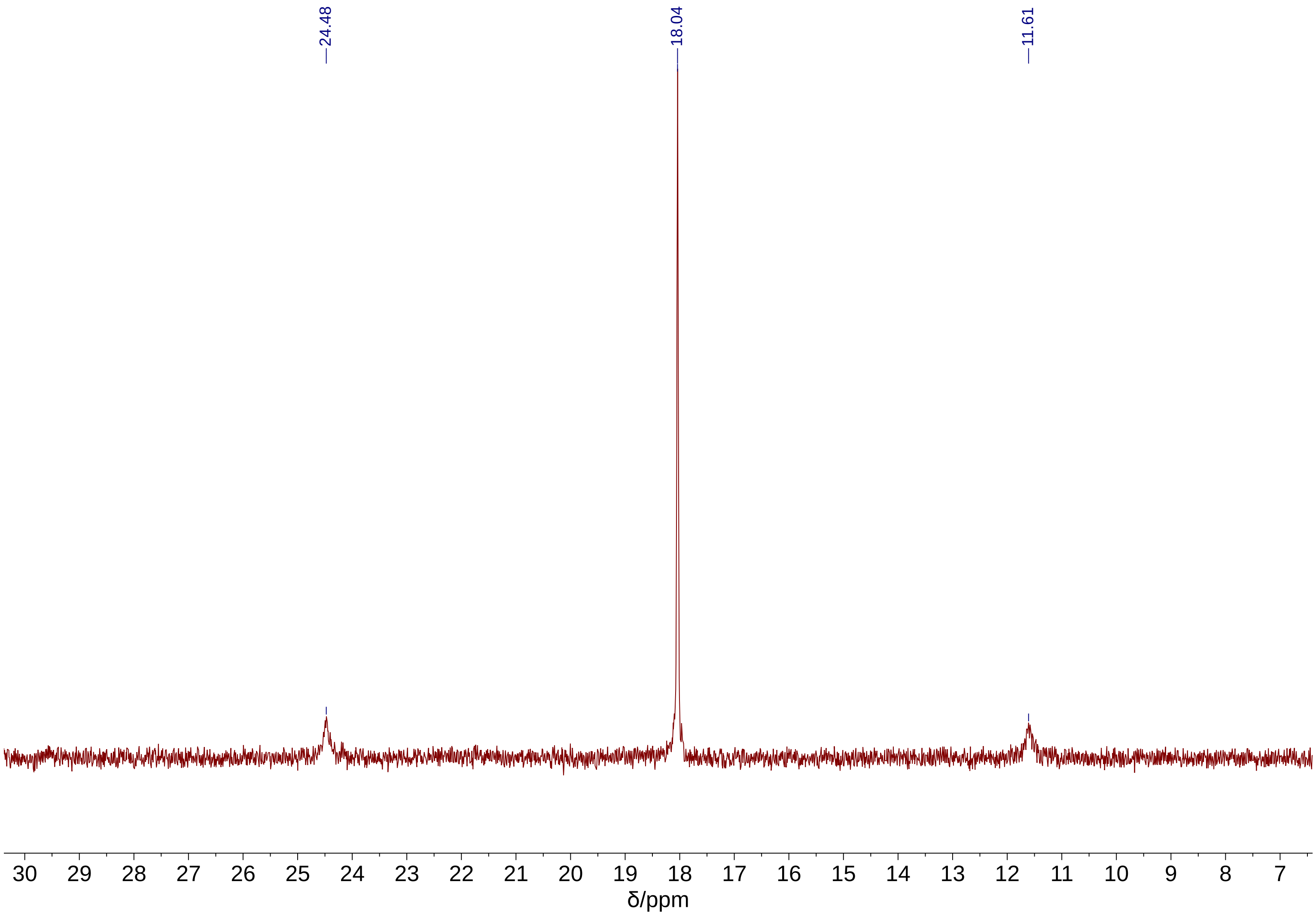

Figure s39. $^{31}P\{^{1}H\}$ NMR spectrum of **$PtC_{40}Pt$** ($CDCl_3$, 202 MHz).

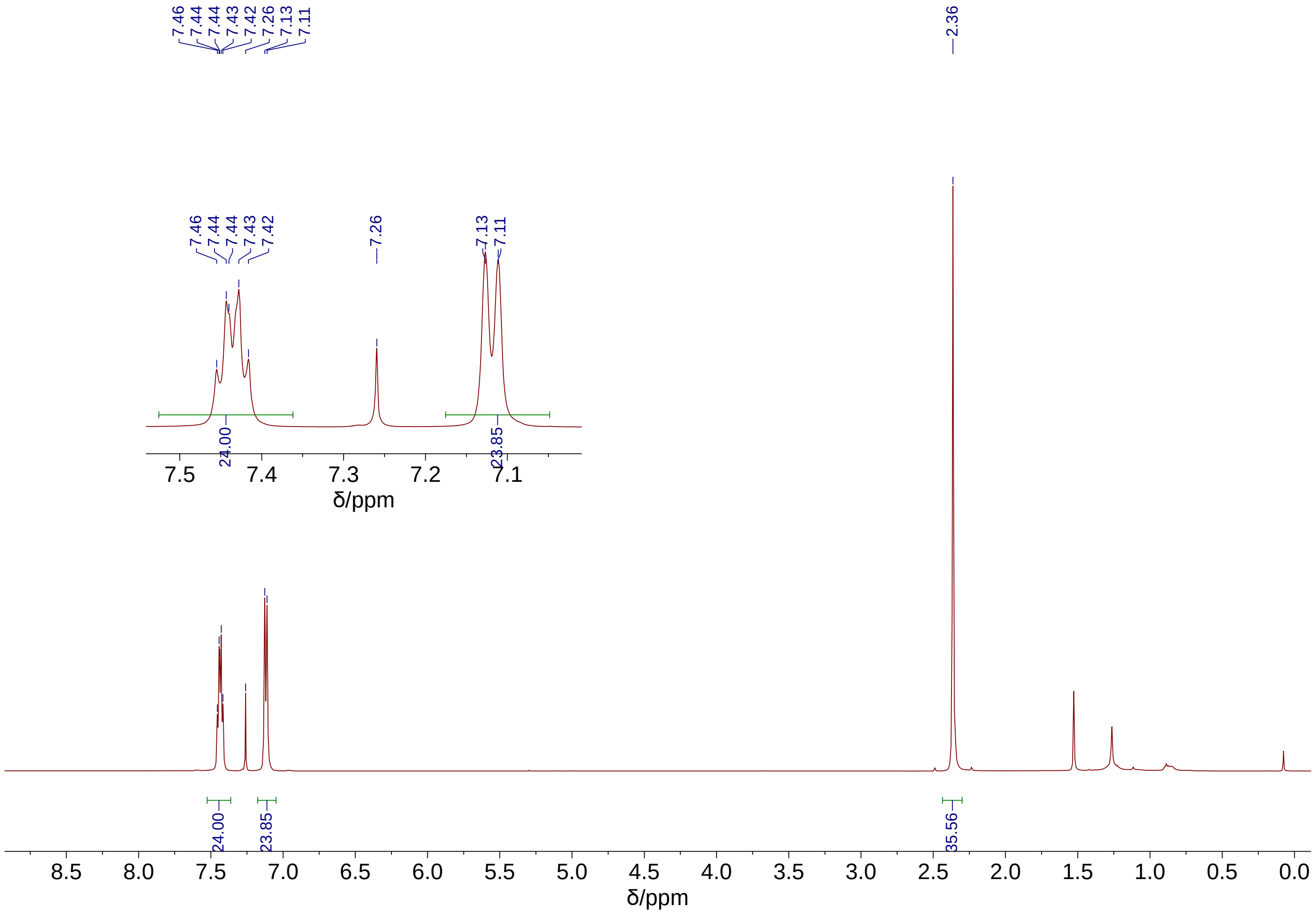

Figure s40. ^{1}H NMR spectrum of **$PtC_{44}Pt$** ($CDCl_3$, 500 MHz).

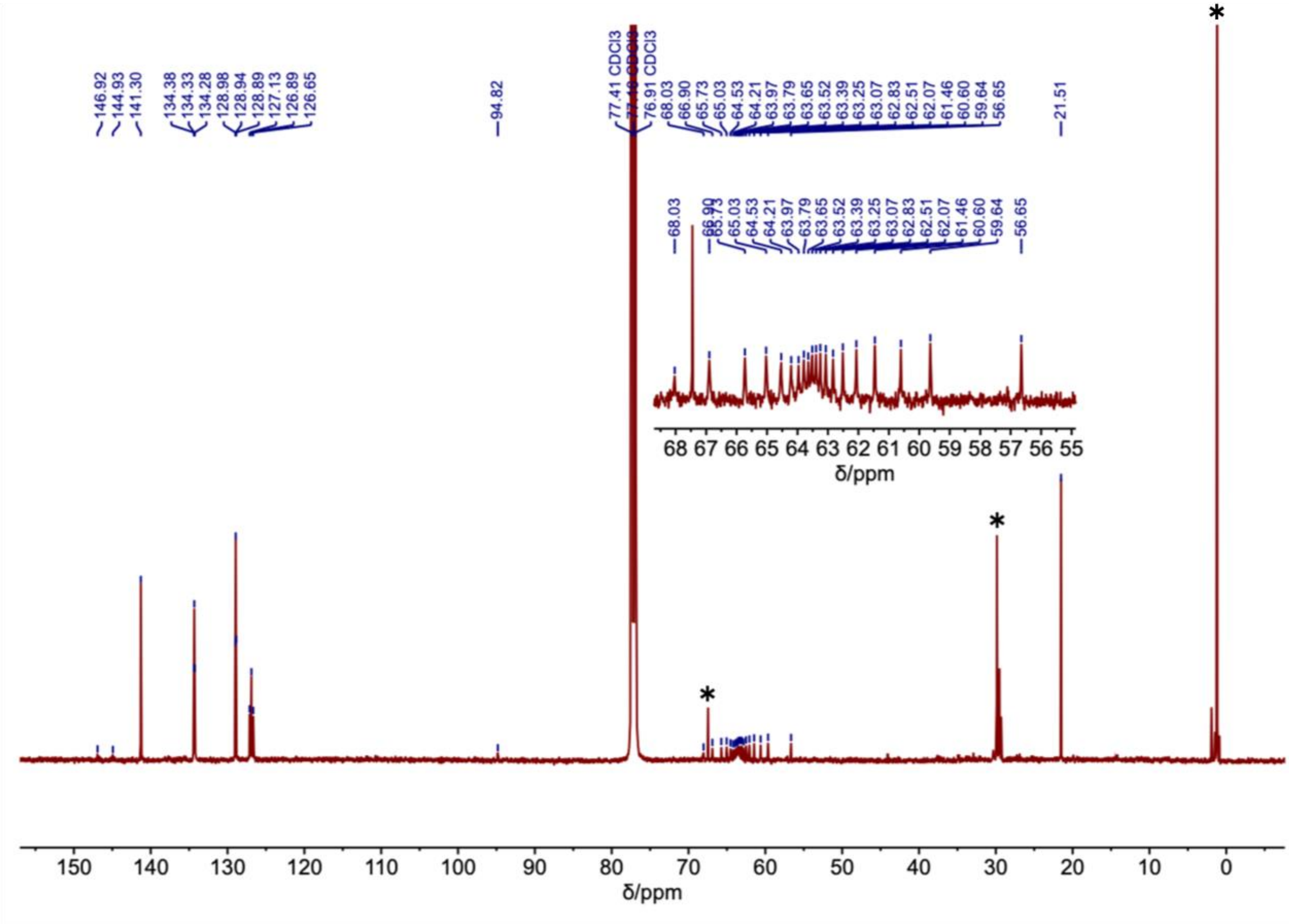

Figure s41. $^{13}C\{^1H\}$ NMR spectrum of **$PtC_{44}Pt$** ($CDCl_3$, 126 MHz). * denotes solvent-based impurities.

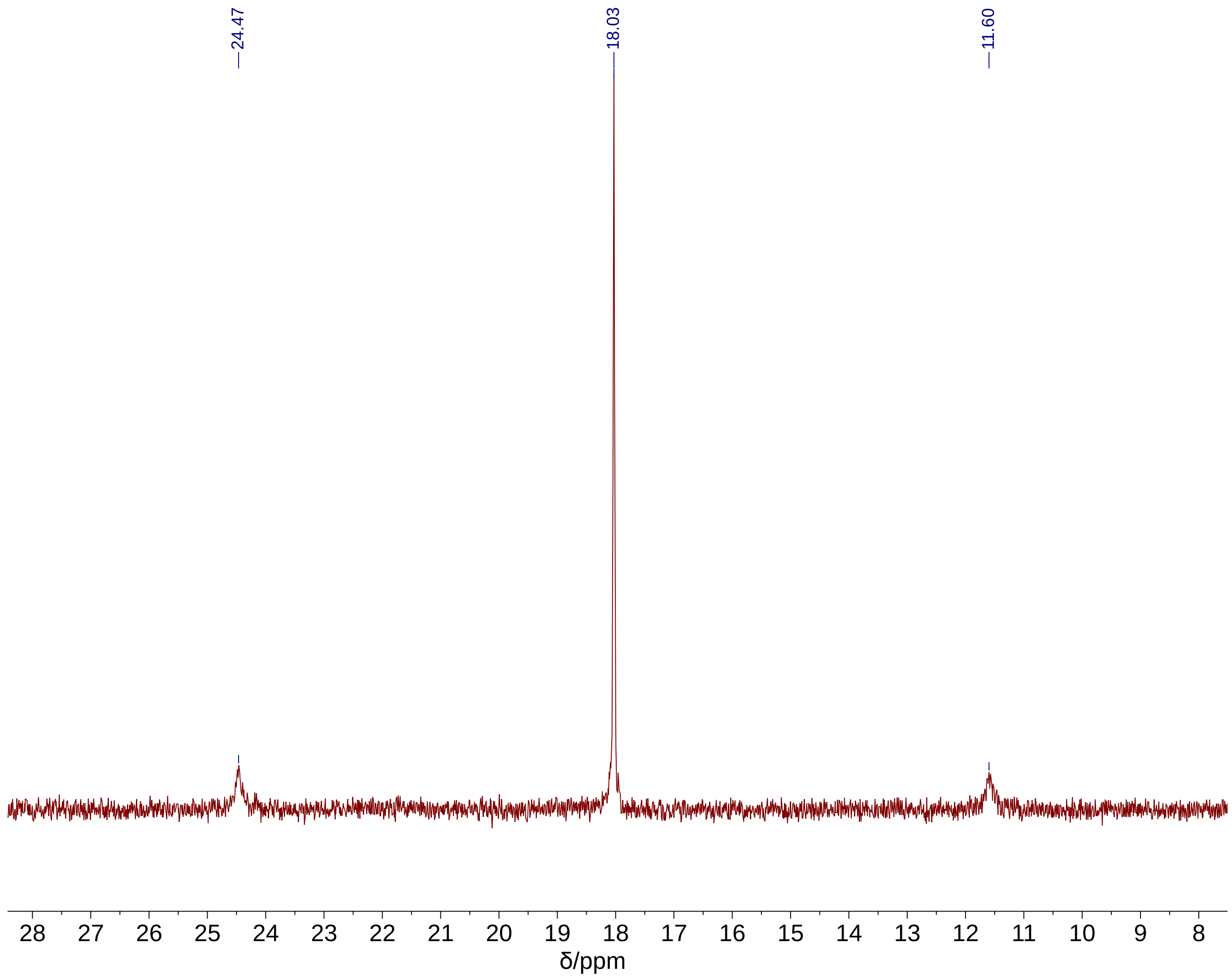

Figure s42. $^{31}P\{^1H\}$ NMR spectrum of **$PtC_{44}Pt$** ($CDCl_3$, 202 MHz).

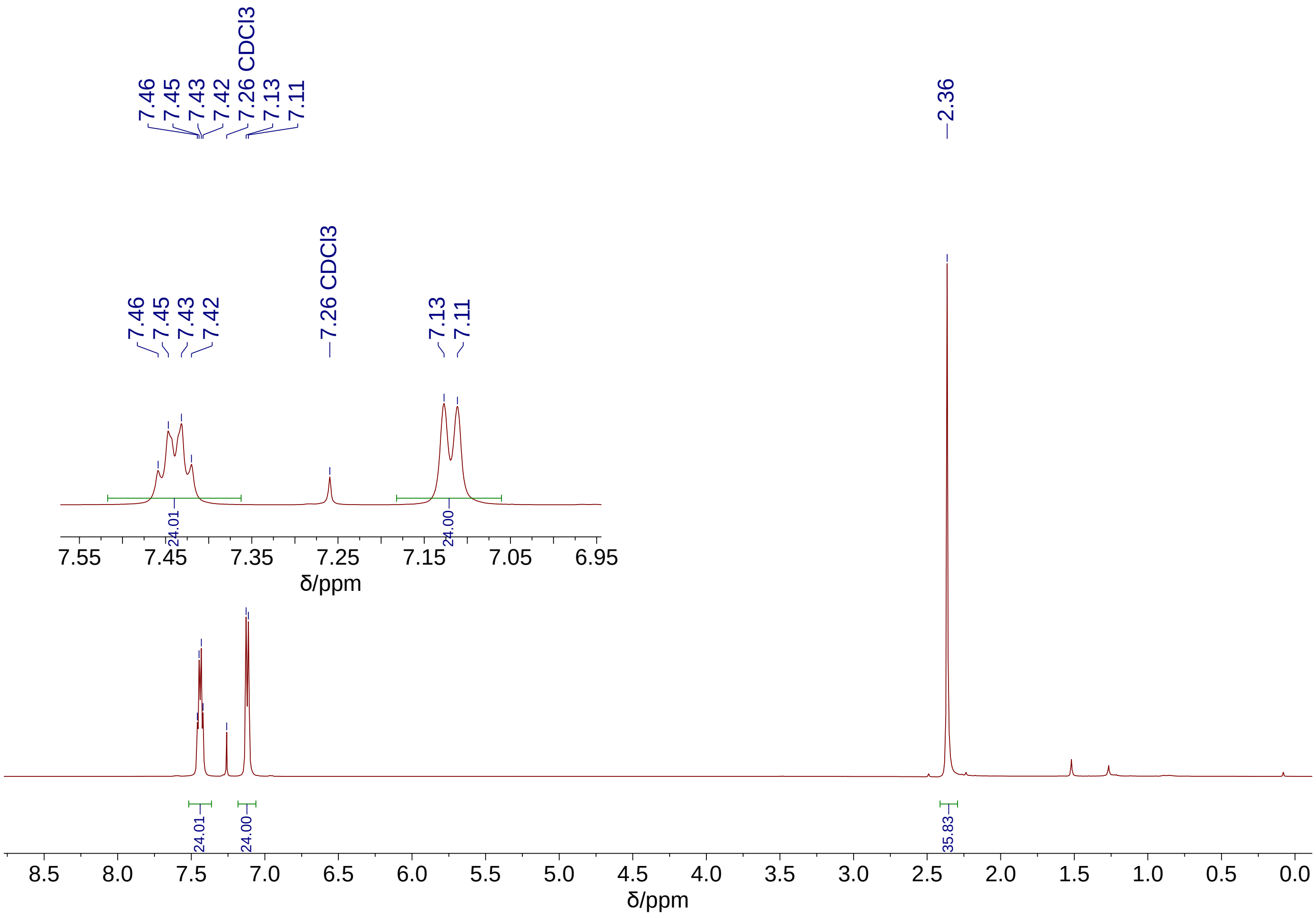

Figure s43. ^{1}H NMR spectrum of **$PtC_{48}Pt$** ($CDCl_3$, 500 MHz).

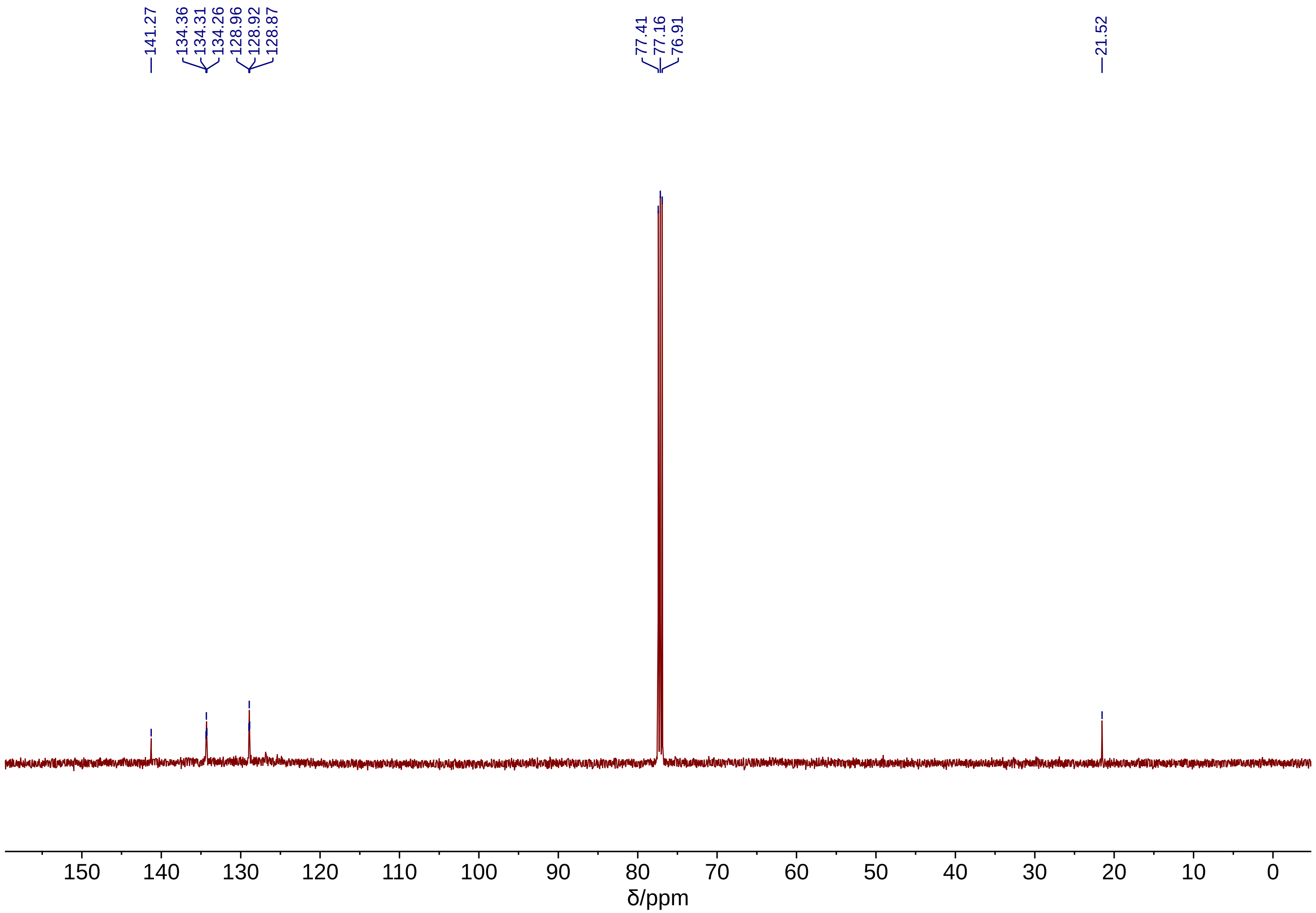

Figure s44. $^{13}C\{^{1}H\}$ NMR spectrum of **$PtC_{48}Pt$** ($CDCl_3$, 126 MHz).

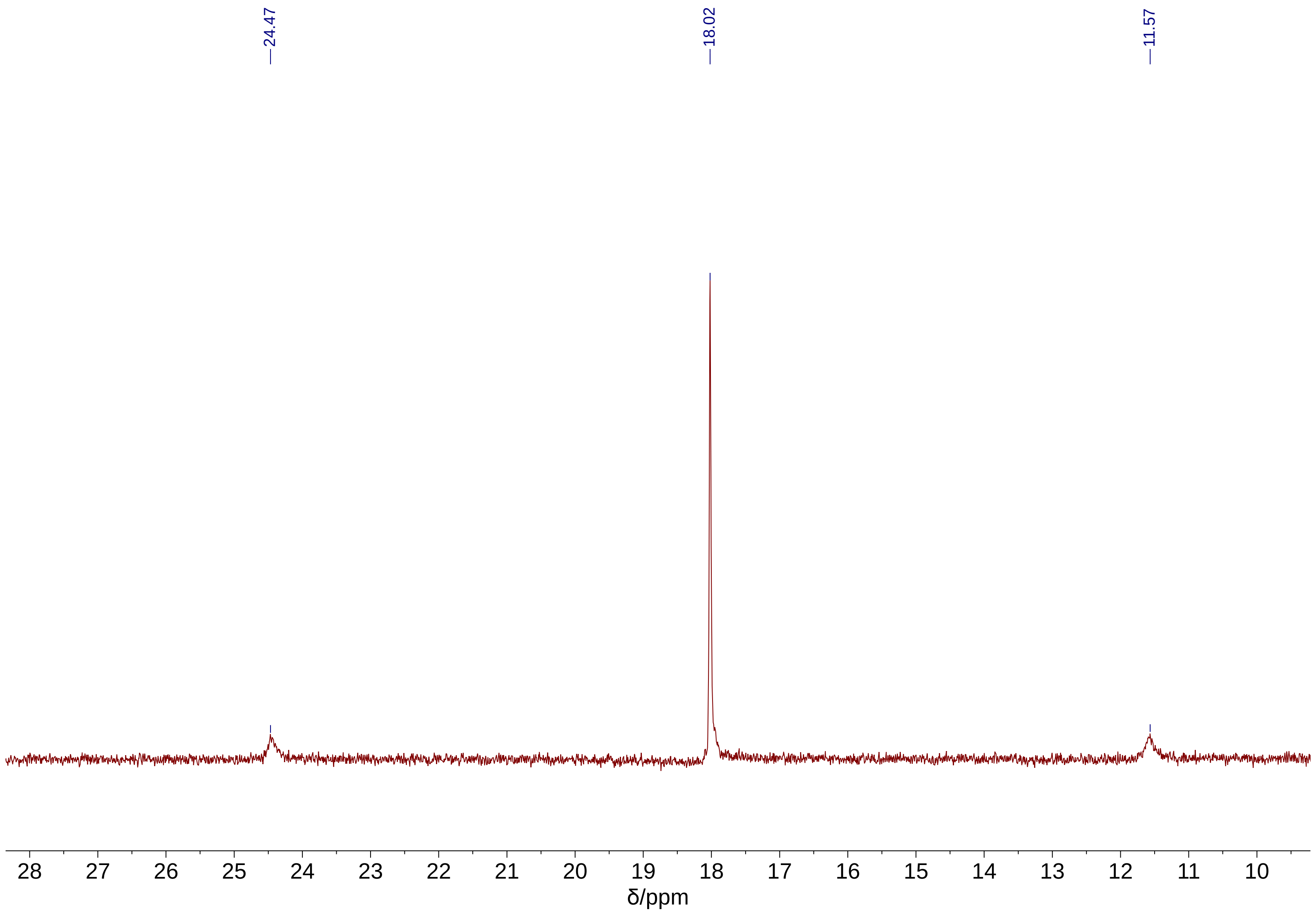

Figure s45. $^{31}P\{^{1}H\}$ NMR spectrum of **$PtC_{48}Pt$** ($CDCl_3$, 202 MHz).

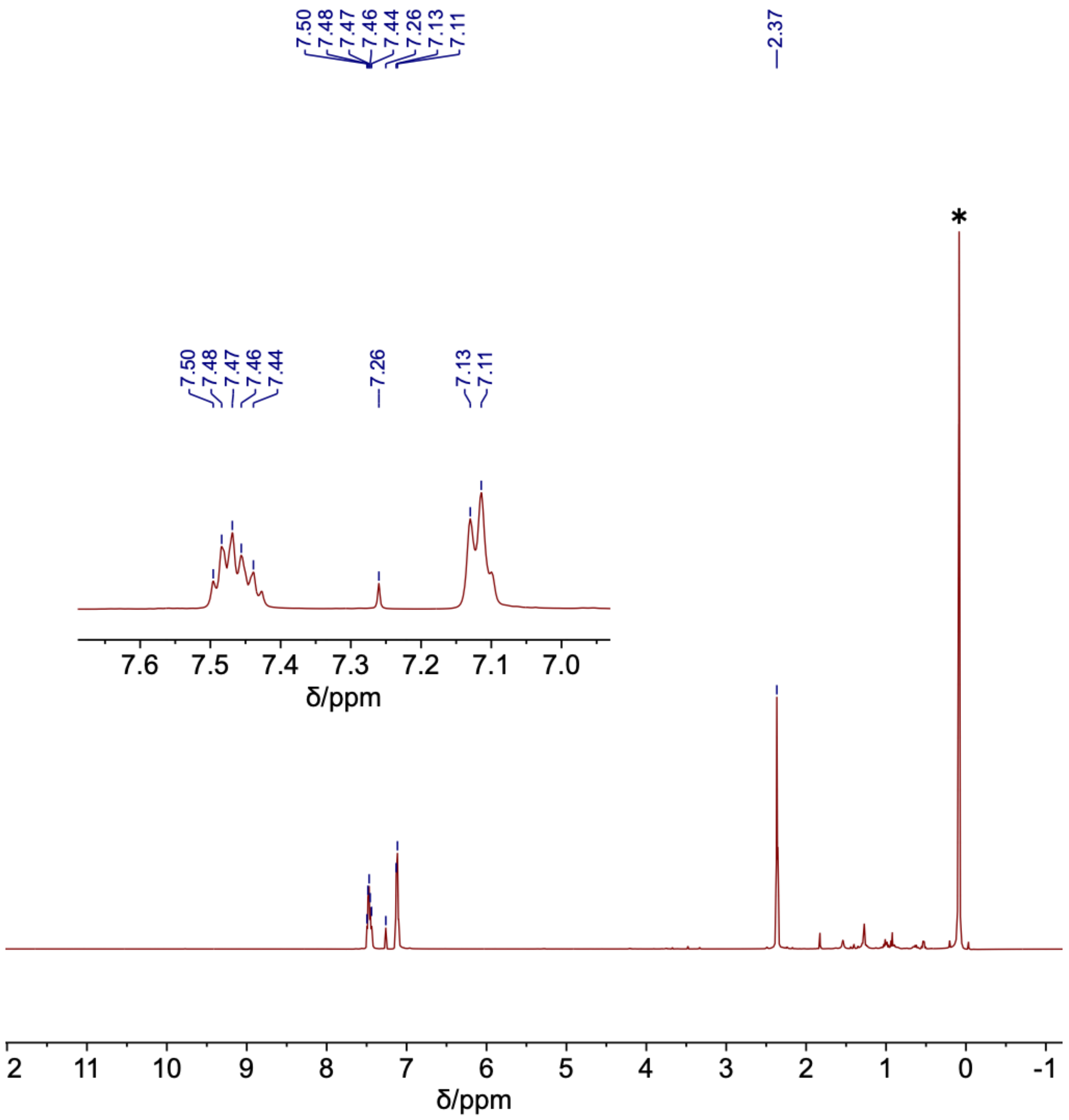

Figure s46. ^{1}H NMR spectrum of **PtC$_{52}$Pt** ($CDCl_3$, 500 MHz). * denotes solvent-based impurities.

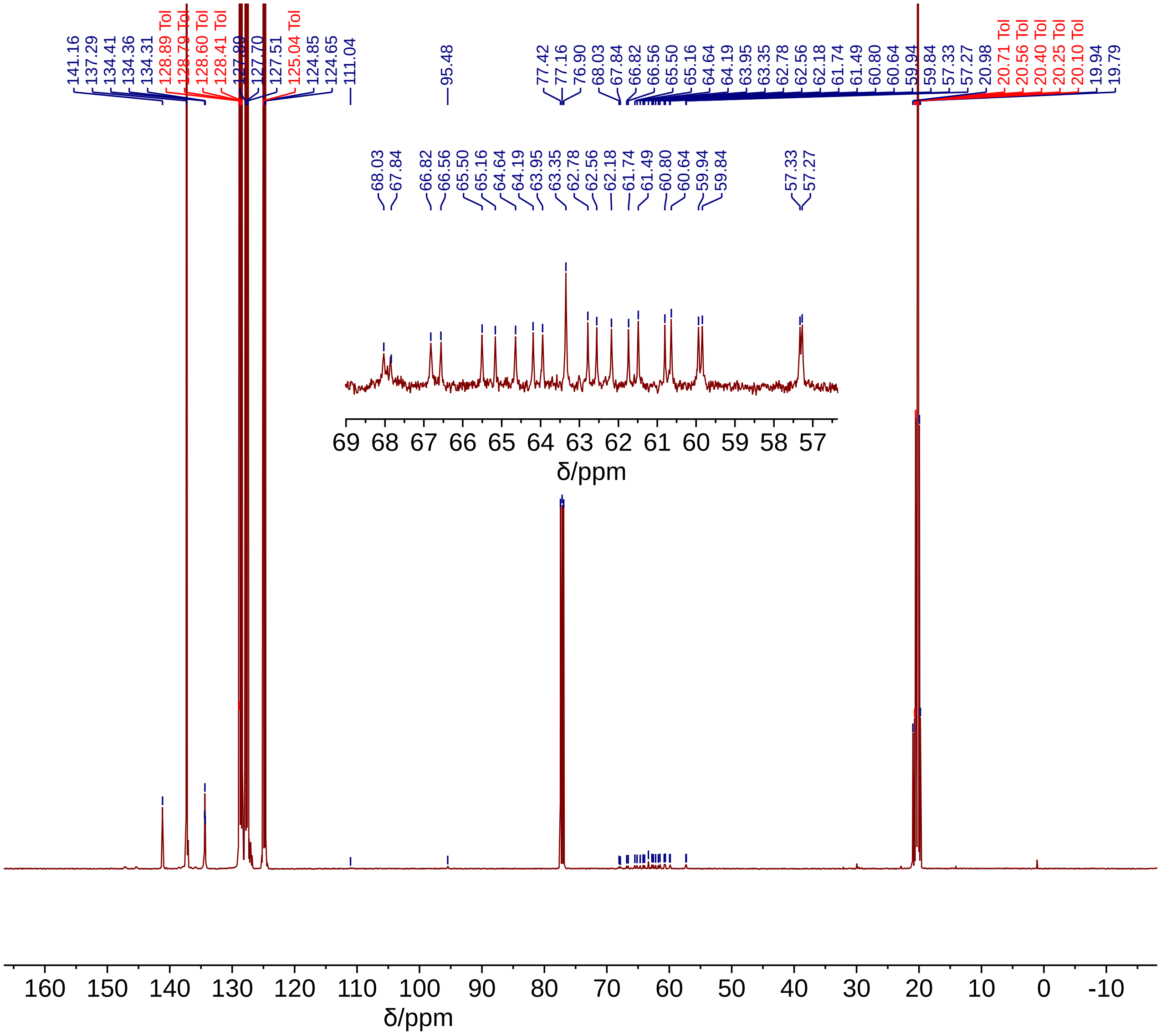

Figure s47. $^{13}C\{^{1}H\}$ NMR spectrum of **$PtC_{52}Pt$** ($CDCl_3$/ tol-d_8 (1:1 v/v), 126 MHz, solvent peaks have been truncated).

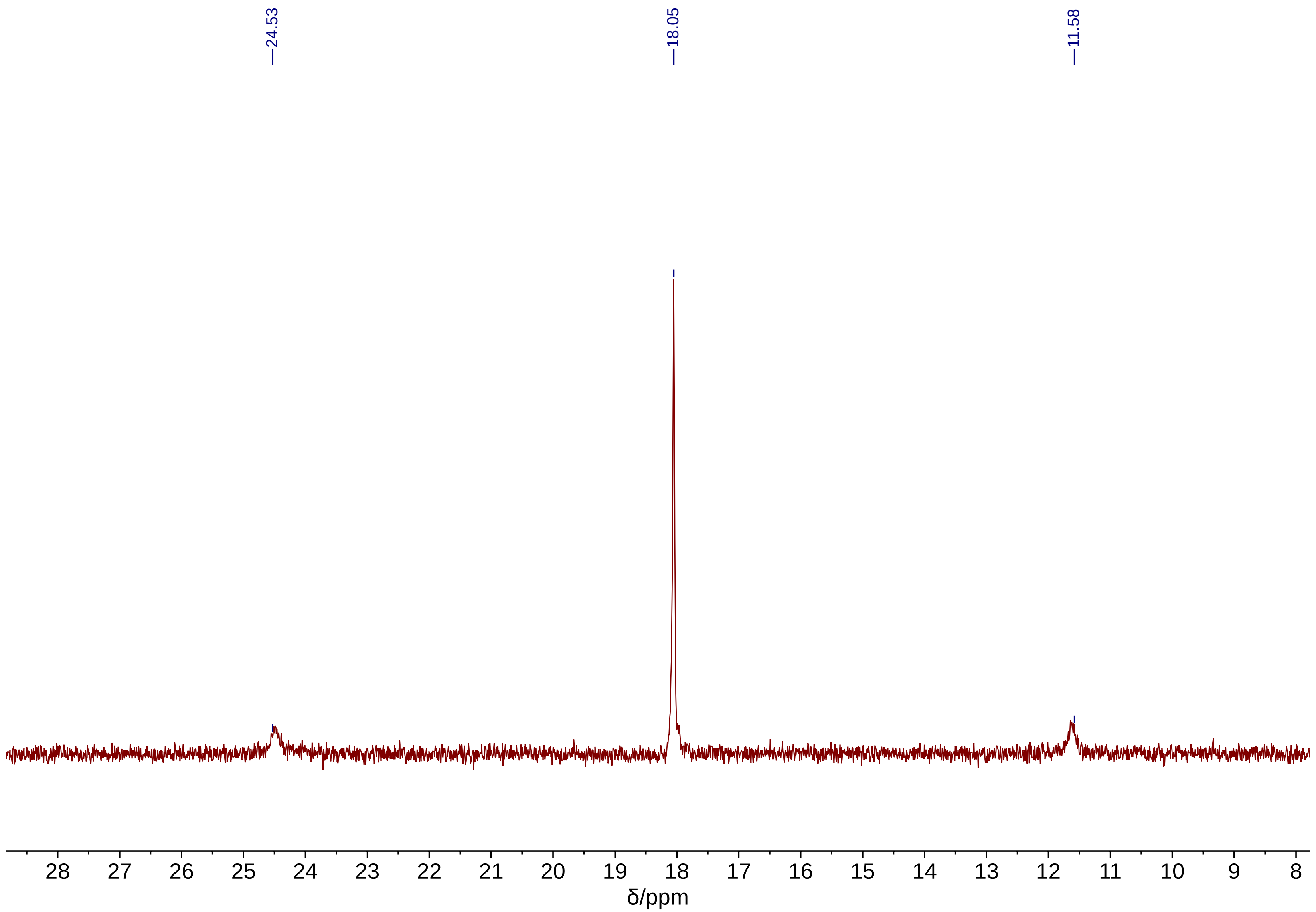

Figure s48. $^{31}P\{^{1}H\}$ NMR spectrum of **$PtC_{52}Pt$** ($CDCl_3$, 202 MHz).
